# CO to CO_2_ Conversion at a Dinickel(II) Complex with Bridging Hydroxide—Emulating a Key Step of [Ni,Fe]-CO Dehydrogenase

**DOI:** 10.1021/acs.inorgchem.6c01897

**Published:** 2026-08-20

**Authors:** Sara I. Mozzi, Martin Diefenbach, Chung-Wei Fu, Sebastian Dechert, Michael John, Vera Krewald, Franc Meyer

**Affiliations:** † 9375 University of Göttingen, Institute of Inorganic Chemistry, Tammannstraße 4, 37077 Göttingen, Germany; ‡ 26536 TU Darmstadt, Department of Chemistry, Quantum Chemistry, 64287 Darmstadt, Germany; § University of Göttingen, International Center for Advanced Studies of Energy Conversion (ICASEC), Tammannstraße 6, 37077 Göttingen, Germany

## Abstract

Late transition metal hydroxides are important intermediates in many catalytic processes. In [Ni,Fe] carbon monoxide dehydrogenase enzymes (CODHs), which catalyze the reversible CO/CO_2_ interconversion, CO insertion in the Ni–(μ–OH) bond of a Ni/Fe-bridging hydroxide is the proposed C–O bond-forming step during CO oxidation. Here, we report that CO reacts with the dinickel­(II) complex LNi_2_(μ–OH) (L^3–^ is a pyrazolato-centered ligand with two {N_3_} compartments), finally leading to HLNi^II^(CO); this is coupled to the formation of CO_2_ and Ni­(CO)_4_, which is the driving force of the overall reaction. Mechanistic details have been investigated by ^13^CO labeling experiments and DFT calculations, and the product derivative HLNi^II^(CNBn) has been crystallographically characterized. The findings demonstrate that a nickel­(II)-bound μ–OH can serve as a nucleophile toward CO and promote its oxidation to CO_2_, which is suggested to proceed via a metal-bridging O-protonated carbonite intermediate, emulating a key step of [Ni,Fe]-CODH reactivity. For CO_2_ release, in the present system, one of the nickel ions serves as a terminal electron sink and the corresponding ligand {N_3_} site of L^3–^ as a proton acceptor.

## Graphical abstract

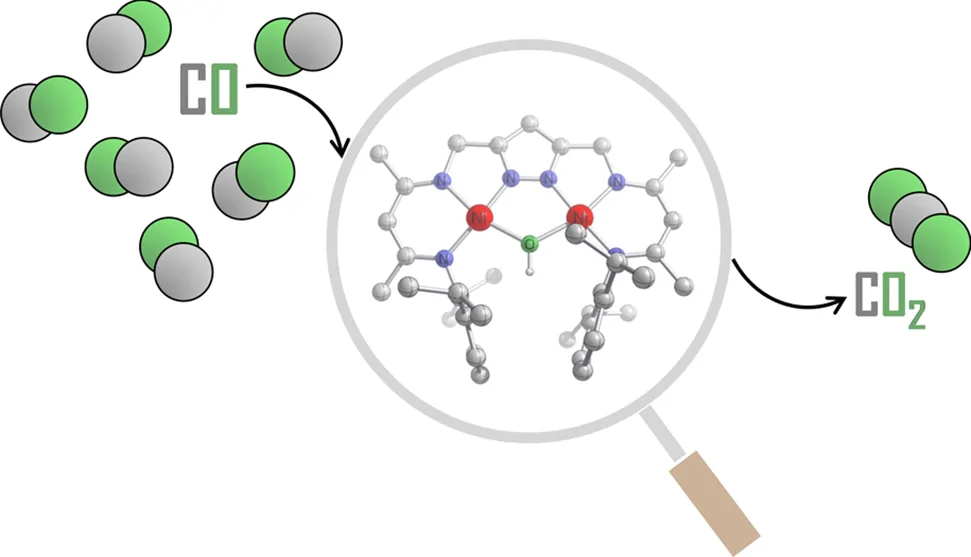

## Introduction

Late transition-metal hydroxido complexes are often considered by organometallic chemists as undesired products and the results of hydrolysis or careless work. However, the isolation of monomeric terminal hydroxido complexes of group 10 transition metals is challenged by their tendency to associate into higher nuclearity systems.^1–8^ Relatively few examples of square-planar Ni or Pd complexes with a terminal hydroxido ligand were successfully isolated by using sterically demanding tridentate pincer ligands, which prevent the formation of multimetallic clusters.^9–15^

Over the last decades, discrete M–OH species, together with M–alkoxide species, have gained interest as reactive intermediates in many catalytic processes.^16–18^ In particular, late transition M–OH bonds play an important role in the Wacker process for the oxidation of olefins to carbonyl compounds, and in the homogeneous water-gas shift reaction (WGSR), where carbon monoxide (CO) and water form carbon dioxide (CO_2_) and molecular hydrogen (H_2_) (eq 1). In nature, the carbon monoxide dehydrogenase (CODH) enzymes reversibly catalyze the CO/CO_2_ interconversion which, in contrast to the WGSR, produces/consumes protons and electrons (eq 2).^19^

***WGSR***

1 $${\mathrm{CO}+H}_{2}O⇄{\mathrm{CO}}_{2}+H_{2}$$

***CODH Activity***

2 $${\mathrm{CO}+H}_{2}O⇄{\mathrm{CO}}_{2}+{2H}^{+}+{2e}^{-}$$

The active site of several [Ni,Fe]-CODHs consists of the so-called C-cluster, which features a coordinatively unsaturated nickel ion and a unique iron site kept in close proximity by an Fe_3_S_4_ cluster fragment.^20,21^ CO oxidation (or CO_2_ reduction) occurs at this cooperative bimetallic subsite (Scheme 1).^22–24^ While details of the mechanism including the relevant metal oxidation states have been controversial, the coordination of CO to the nickel center (C_red1_ → C_red1_-CO) is generally assumed to be followed by the nucleophilic attack of a metal-bound hydroxide.^22^ In a recent scenario based on atomic resolution crystallography of all catalytically relevant states of a [Ni,Fe]-CODH with and without substrates,^25^ the hydroxide in C_red1_ adopts a bridging position between the Ni^II^ and Fe^II^ ions. Via the short-lived C_red1_-CO state and subsequent deprotonation of the COOH ligand in C_red2_-H^+^, which results from nucleophilic attack, a metal-bridging carbonite intermediate (C_red2_) is formed.^26^ Release of CO_2_ and sequential loss of two electrons to the B- and D-clusters, which are [Fe_4_S_4_] clusters serving as electron relays from/to the C-cluster, regenerates C_red1_ via the proposed Ni^I^ intermediate C_int,o_.^21,25^ Hydrogen bonding interactions with amino acid residues surrounding the C-cluster play an important role in stabilizing several intermediates and enabling proton transfer. Hence, in the proposed key C–O bond-forming step during CO oxidation by [Ni,Fe]-CODHs, the CO substrate reacts by inserting into the Ni–(μ–OH) bond, forming the Ni–C­(O)­O­(H)–Fe unit, which readily deprotonates. Furthermore, the nickel ion is the redox-active element in the catalytic cycle.

**Scheme 1:**
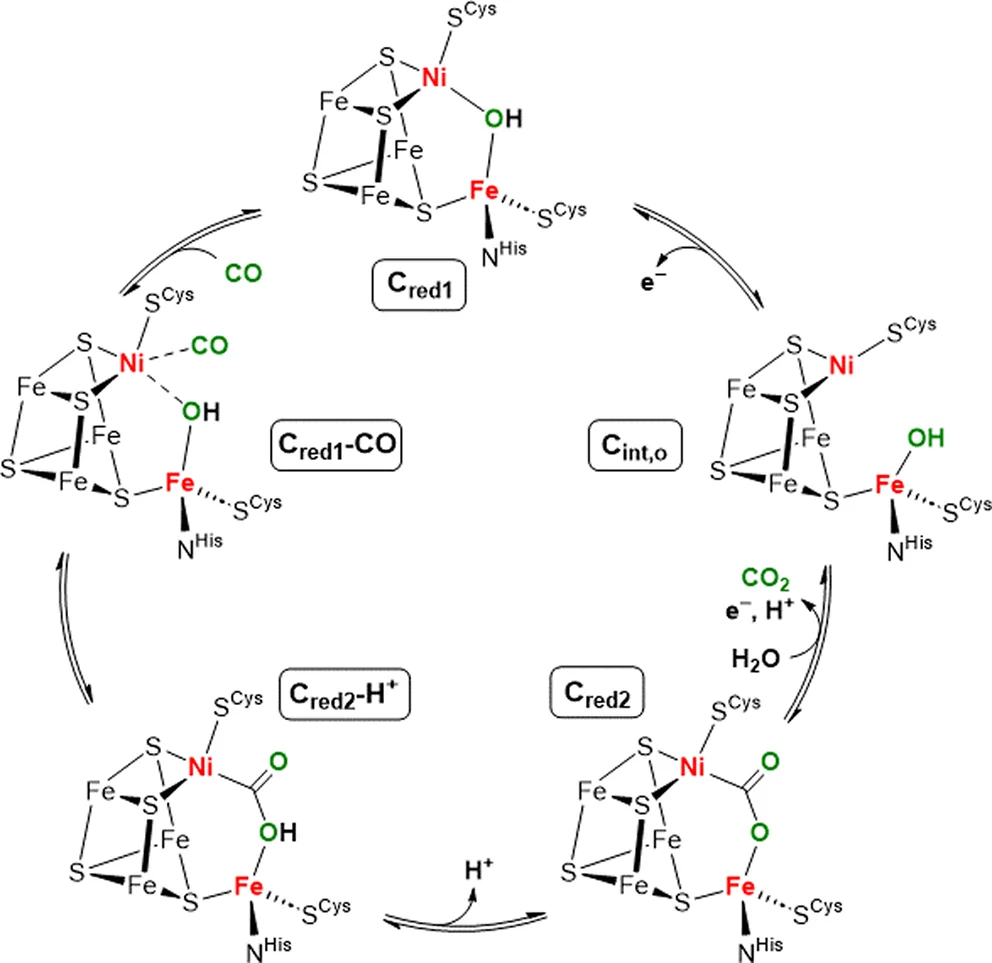
Proposed Catalytic Cycle of the Ni–Fe CODH at the C-Cluster Active Center

In the present work, we emulate the metal–metal cooperative C–O bond-forming and CO_2_-releasing steps of [Ni,Fe]-CODHs, using a highly preorganized hydroxido-bridged dinickel­(II) site. This system is based on a pyrazolato/bis-β-diketiminato ligand scaffold L^3–^ recently developed in our group, which can host two metal ions (i.e., Ni^II^ ions) in close proximity to create a substrate-binding pocket and exploit metal–metal cooperativity for the activation and transformation of small molecules (Scheme 2). For example, the dinickel­(II) dihydrido complex **I** acts as a masked dinickel­(I) complex **II** and, upon H_2_ release, can reduce a variety of substrates by 2e^–^ within the bimetallic pocket, as schematically shown in complex **III**.^27–31^ **I** also reacts with H_2_O, hence studying the reactivity of complexes **I** and **II** requires strictly anhydrous conditions to prevent the formation of the μ-hydroxido complex **1**. Indeed, controlled addition of a small amount of H_2_O initially results in the unusual hydrido/hydroxido dinickel­(II) complex **IV**.^32^

**Scheme 2:**
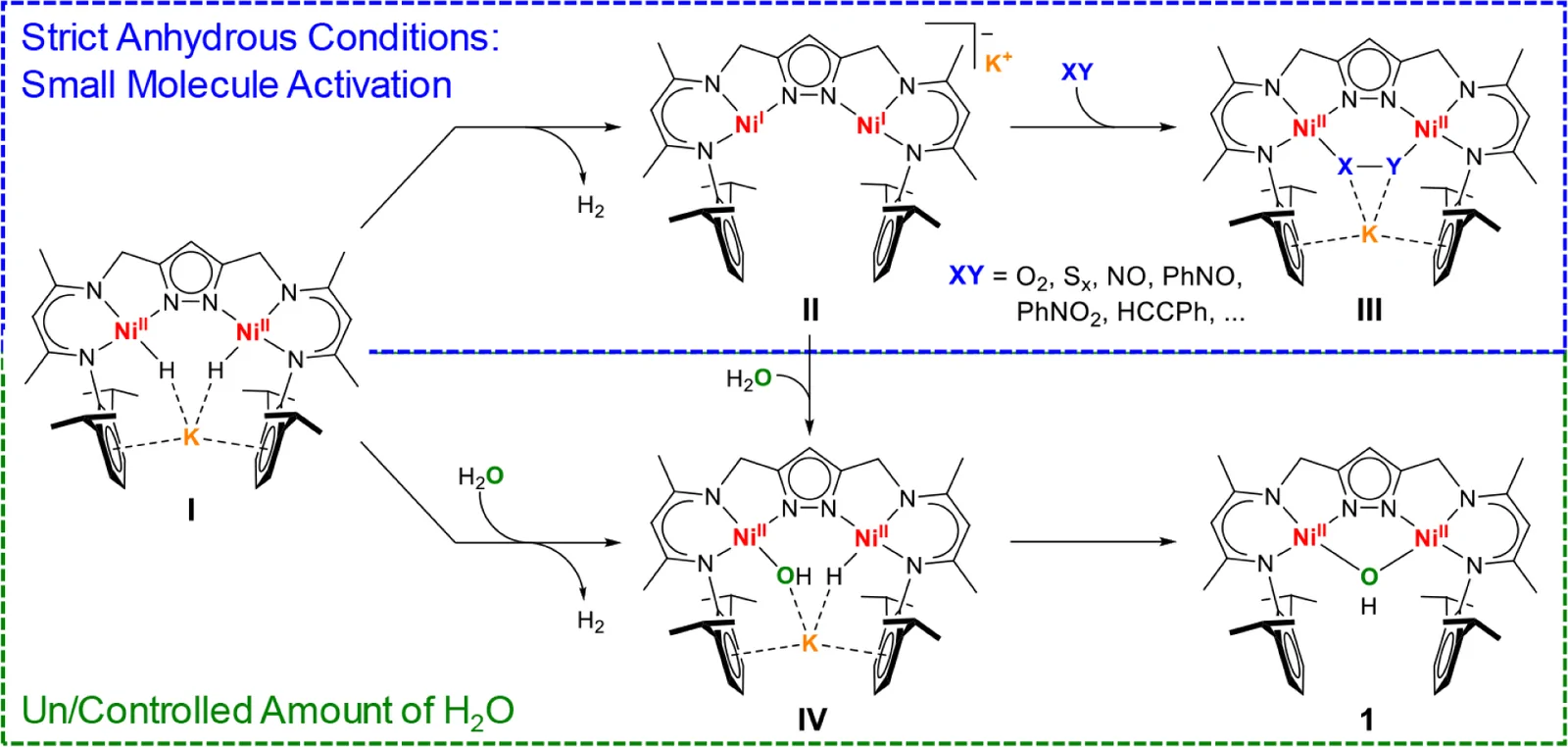
Two-Fold Reductive Small Molecule (XY) Activation by the Dinickel­(II) Dihydride Complex I and Competitive Reaction with Water

Complex **IV** evolves in the presence of excess water or undergoes thermal decomposition at temperatures above −10 °C to finally give complex **1**, which has usually been considered a thermodynamic sink in the chemistry of this dinickel pyrazolato/bis-β-diketiminato platform. However, when studying the reactivity of dinickel complexes **I** and **II** toward CO, where adventitious H_2_O is very challenging to avoid, only a minor amount of **1** was detected. This unexpected observation prompted us to study the reaction between complex **1** and an excess of dry CO with the perspective of exploring, for the first time, a follow-up reactivity of complex **1**.

## Results and Discussion

### Synthesis and Characterization

Since **1** is the starting complex of the present study, a targeted high-yielding synthesis has been developed to circumvent the extra step of preparing the highly reactive complex **I**. To that end, complex **1** is now synthesized by the reaction of the previously reported LNi_2_(μ-Br) with aqueous KOH in THF.^27^ Addition of dry CO to a toluene-d_8_ solution of **1** leads to a color change from intense olive-green to a faded sage-green within several days. ^1^H NMR spectroscopy shows the clean conversion to **2** ^**CO**^ with a spectroscopic yield close to 100% (Scheme 3; for spectroscopic yield determination, see SI Section 2.2). The C*H* ^nacnac^ resonances of the two β-diketiminato subunits at 4.81 and 4.69 ppm, the two signals for the C*H*_2_ bridges at 4.43 and 3.87 ppm, as well as the two signals for the C*H* ^dipp^ resonances of the ^*i*^Pr group units at 3.46 and 3.17 ppm indicate C_*s*_ symmetry where the two {N_3_} compartments of L^3–^ are not equivalent, which is compatible with the loss of one nickel ion. Protonation at the central N atom of the side arm at the vacant binding site is confirmed by ^1^H–^15^N HMBC spectroscopy, where a correlation between a nitrogen atom resonating at 274.2 ppm and the N–*H* signal at 11.31 ppm is observed (Figure 1a); the chemical shift of the latter is in line with intramolecular H-bonding within the β-diketiminato subunit (vide infra). In addition, the N–*H* triplet signal and the C*H*_2_ doublet at 4.43 ppm show a mutual ^3^ *J*_HH_ coupling constant of 5.8 Hz, further confirmed by a ^1^H–^1^H COSY experiment (Figure 1b).

**Scheme 3:**
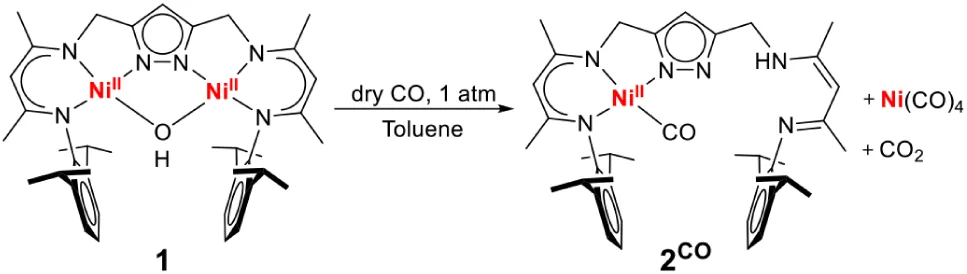
Reaction of 1 with CO Leading to the Formation of CO_2_, Ni­(CO)_4_ and 2^CO^

**Figure 1:**
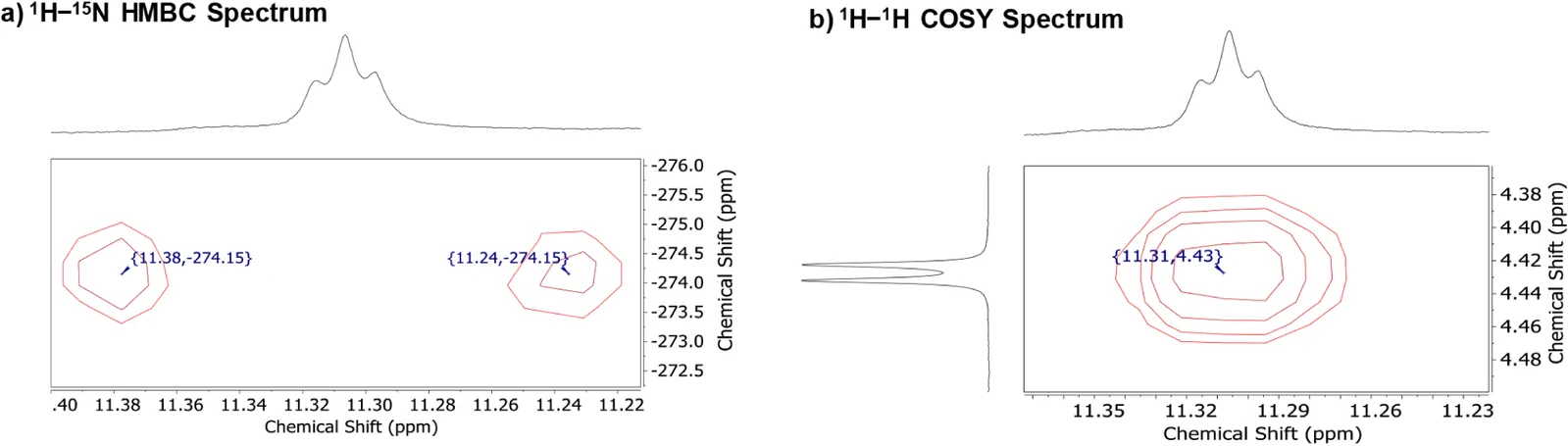
(a) Section of the ^1^H–^15^N HMBC NMR spectrum of **2^CO^** in toluene-d_8_ showing the one-bond *H*–N correlation (^1^ *J*_HN_ = −88 Hz). (b) Section of the ^1^H–^1^H COSY NMR spectrum of **2^CO^** in toluene-d_8_ showing the correlation between the N–*H* triplet and the C*H*_2_ doublet signals.

^13^C­{^1^H} NMR spectroscopic analysis of the reaction mixture of **1** and ^13^CO reveals the formation of Ni­(CO)_4_ at 192.2 ppm, with a satellite quartet (*J* = 120 Hz) due to coupling with ^61^Ni (*I* = 3/2, 1% abundance), and the signal for CO_2_ at 125.2 ppm (Figure 2).^33,34^ The resonance at 176.4 ppm is assigned to the CO ligand in **2** ^**CO**^ by ^1^H–^13^C HMBC spectroscopy, which shows correlations of the CO ligand to the pyrazolate-*H* ^4^ at 6.07 ppm, as well as to the C*H*_2_ protons at 3.87 ppm and one methyl group at 1.42 ppm (Figure S10). ^13^C-DOSY NMR spectroscopy further supports the assignment of the resonances in the ^13^C­{^1^H} NMR spectrum (Figure 2). In accordance with their size, CO and CO_2_ have the highest diffusion coefficients (4.77 ± 0.05·10^–9^ m^2^s^–1^ and 4.0 ± 0.2·10^–9^ m^2^s^–1^ respectively), followed by toluene (1.86 ± 0.02·10^–9^ m^2^s^–1^), Ni­(CO)_4_ (1.75 ± 0.02·10^–9^ m^2^s^–1^), and **2** ^**CO**^ (5.5 ± 0.1·10^–10^ m^2^s^–1^). The NMR experiments collectively support the formation of **2** ^**CO**^, which hosts one d^8^ nickel­(II) ion in a square planar geometry within a tridentate {N_3_} pocket and with CO completing the coordination sphere.

**Figure 2:**
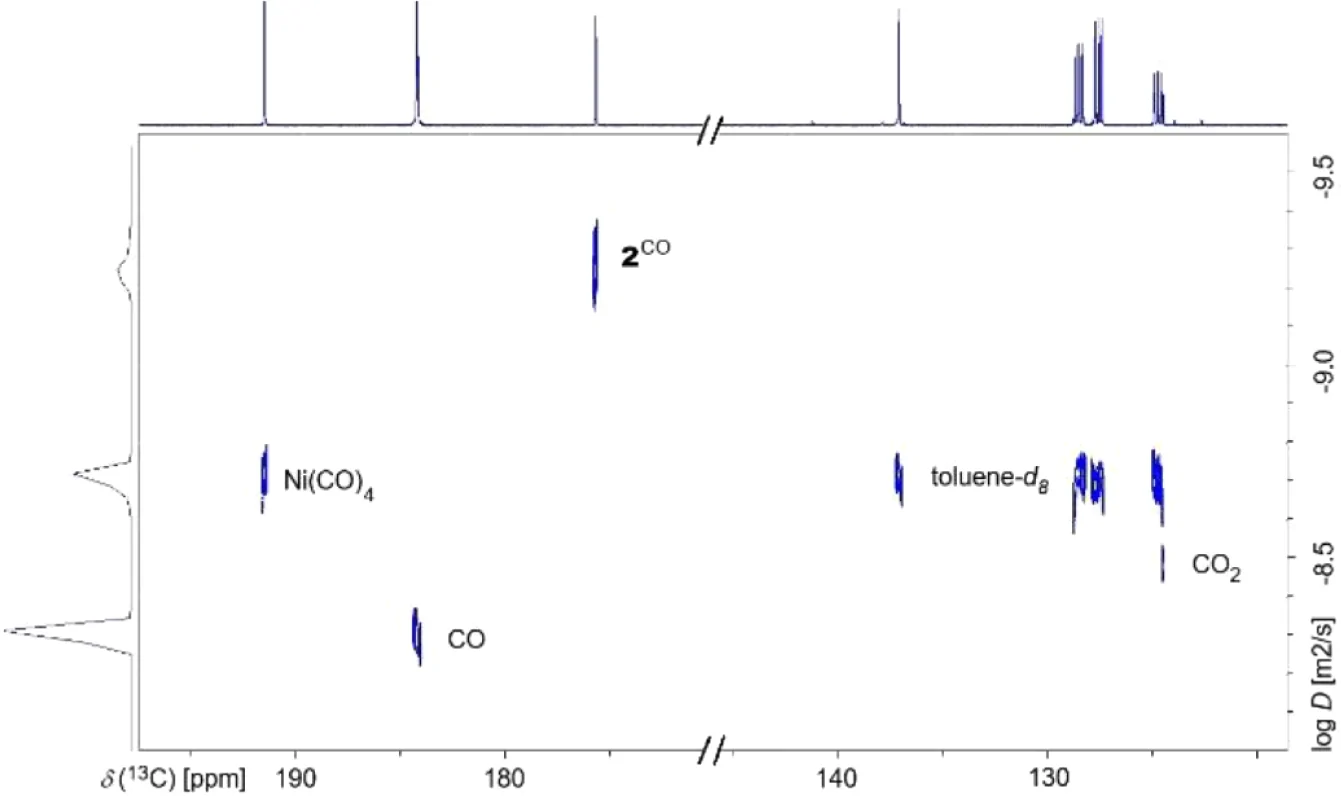
^13^C­{^1^H} DOSY NMR spectrum of the reaction mixture of **1** with ^13^CO in toluene-d_8_.

The products of the reaction of **1** and CO were further characterized by IR spectroscopy. An IR spectrum recorded in the gas phase shows the typical CO_2_ asymmetric stretching at 2359 cm^–1^ and bending at 729 cm^–1^. Labeling experiments reacting **1** with ^13^CO result in a shift of the CO_2_ stretching to 2239 cm^–1^, confirming that the CO_2_ originates from CO under these reaction conditions. Furthermore, the formation of CO_2_ was supported by gas chromatography (GC) analysis of the headspace of the reaction mixture (Figures S28–S29). Ni­(CO)_4_ was isolated via vacuum distillation of the reaction mixture. An IR spectrum of the colorless solution displays the signature stretching of Ni­(CO)_4_ at 2043 cm^–1^ (Δ*ṽ*(^13^CO–^12^CO) = −47 cm^–1^, Figure S30). The presence of a CO ligand in **2** ^**CO**^ is confirmed by the C–O stretching at 2118 cm^–1^ which shifts to 2070 cm^–1^ upon labeling (Figure S13). This value is only slightly lower than the value of free CO (2143 cm^–1^), indicating weak π-backdonation from the Ni^II^ ion to the empty π*­(CO), and it is comparable to *ṽ*_CO_ found for other neutral and cationic square planar Ni^II^ complexes.^35–37^ Protonation of an N atom of one β-diketiminato side arm leads to localized C=N and C=C units (viz., an α,β-unsaturated imine moiety), which is reflected in a *ṽ*_C=N_ of 1622 cm^–1^, lower compared to *ṽ*_C=N_ found for the β-diimine fragment in {LNi_2_} complexes with a C-protonated ligand backbone.^38–40^

The loss of one nickel ion can also be observed in the electrospray ionization (ESI) mass spectrum of a solution of **2** ^**CO**^, where the peak at *m*/*z* = 665.3 corresponds to the ion [LNi+2H]^+^ (Figure S12). Additional peaks at *m*/*z* = 609.5 for the metal-devoid ion [H_3_L+H]^+^ and at *m*/*z* = 1421.7 are observed, and the latter can be tentatively assigned to the ion [(HL)_2_Ni_3_(OH)_2_+H]^+^, raising a question about the importance of using dry CO during the reaction. When **1** was treated with CO directly from the gas cylinder, without prior drying, the green solution turns brown/yellow within a few hours. While CO_2_ and Ni­(CO)_4_ are detected via GC analysis and ^13^C­{^1^H} NMR spectroscopy, respectively, the ^1^H NMR spectrum of the reaction mixture does not allow identification of the final L^3−^-based species (Figures S31–33). The same ^1^H NMR spectrum is obtained if a sample of **2** ^**CO**^ is exposed to air or moisture. The resulting solution, analyzed by ESI-MS (Figure S35), confirms the presence of protonated free ligand (*m*/*z* = 609.4) and the trinuclear species (HL)_2_Ni_3_(OH)_2_ (**3**; indicated by the ion [(HL)_2_Ni_3_(OH)_2_+H]^+^ at *m*/*z* = 1421.7 in the ESI-MS). IR spectroscopic analysis supports the formation of **3**, displaying characteristic vibrations at *ṽ*_OH_ = 3609 cm^–1^ and *ṽ*_C=N_ = 1626 cm^–1^ (Figure S34) in good agreement with the values for the *ṽ*_OH_ and *ṽ*_C=N_ stretching in **1** and **2** ^**CO**^, respectively. It can thus be concluded that CO to CO_2_ oxidation by **1** proceeds in the presence of water, but that the product **2** ^**CO**^ is moisture sensitive. The identity and structure of the trinuclear product (HL)_2_Ni_3_(OH)_2_ is revealed by the isolation and crystallographic characterization of its adduct with triazabicyclodecene (TBD) (**3** ^**TBD**^; see Figures 3 and S27). ESI-MS of **3** ^**TBD**^ shows a minor signal for [(HL)_2_Ni_3_(OH)_2_(TBD)+H]^+^ at *m*/*z* = 1562.8 as well as a major signal for [(HL)_2_Ni_3_(OH)_2_+H]^+^ at *m*/*z* = 1421.7 (Figure S26).

**Figure 3:**
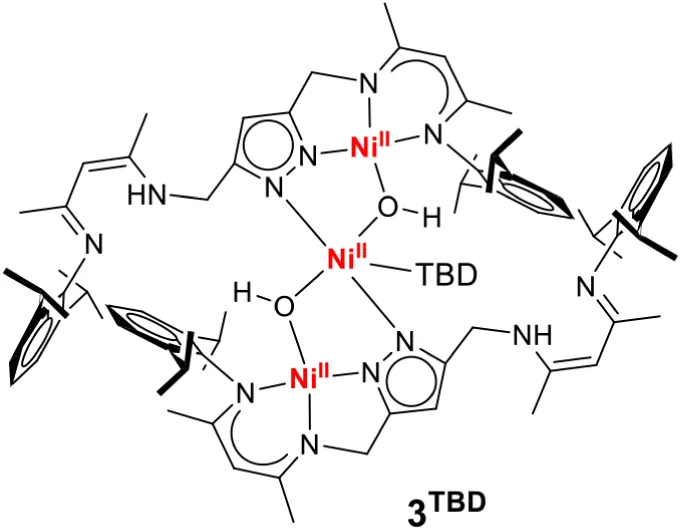
Structure of the TBD adduct (**3^TBD^**) of the trinuclear complex (HL)_2_Ni_3_(OH)_2_ (**3**) that is formed upon exposure of **2^CO^** to air or moisture.

The high solubility of **2** ^**CO**^ prevented the isolation of single crystals for X-ray crystallographic structure determination. Therefore, to provide further support for the molecular structure of **2** ^**CO**^, the CO ligand was exchanged with an isocyanide. Addition of 1 equiv of benzyl isocyanide (CNBn) to a toluene solution of **2** ^**CO**^ caused an immediate color change to bright orange and gas evolution. Pentane diffusion into the toluene solution over 2 weeks at −35 °C gave crystals of HLNi^II^(CNBn) (**2** ^**CNBn**^) suitable for X-ray diffraction analysis (XRD). The molecular structure of **2** ^**CNBn**^ shows the presence of a single Ni^II^ ion in square planar geometry and protonation of the N5 atom of the free β-diketiminato subunit (Figure 4), as was proposed for **2** ^**CO**^ on the basis of NMR spectroscopy (vide supra). The isocyanide binds linearly with a Ni1–C40–N7 angle of 179.4(7). The Ni1–C40 and the C40–N7 bond lengths of 1.831(7) Å and 1.165(9) Å, respectively, are in line with known Ni^II^–isocyanide complexes.^40–42^ The *ṽ*_C≡N_ stretching of 2218 cm^–1^ found in the IR spectrum of crystalline **2** ^**CNBn**^ is higher than the *ṽ*_C≡N_ stretch of free benzyl isocyanide (2190 cm^–1^),^43^ indicating strong σ-donation of the isocyanide to the Ni^II^ with only minor π-backdonation (Figure S23). The bond lengths within the metal-containing β-diketiminato arm involving N3 and N4 (1.332(9) and 1.337(9) Å) are in between those of the localized C–N and C=N bonds involving N5 and N6, respectively, within the dangling arm where N5 is protonated (1.352(8) and 1.311(7) Å). Just as **2** ^**CO**^, complex **2** ^**CNBn**^ shows an IR band at 1622 cm^–1^ assigned to *ṽ*_C=N_ of the localized C=N bond. In addition, a band at 3059 cm^–1^ which is in good agreement with the *ṽ*_N–H_ stretching calculated for the corresponding conformer of **2** ^**CO**^, could be observed (see below for the calculated frequencies).

**Figure 4:**
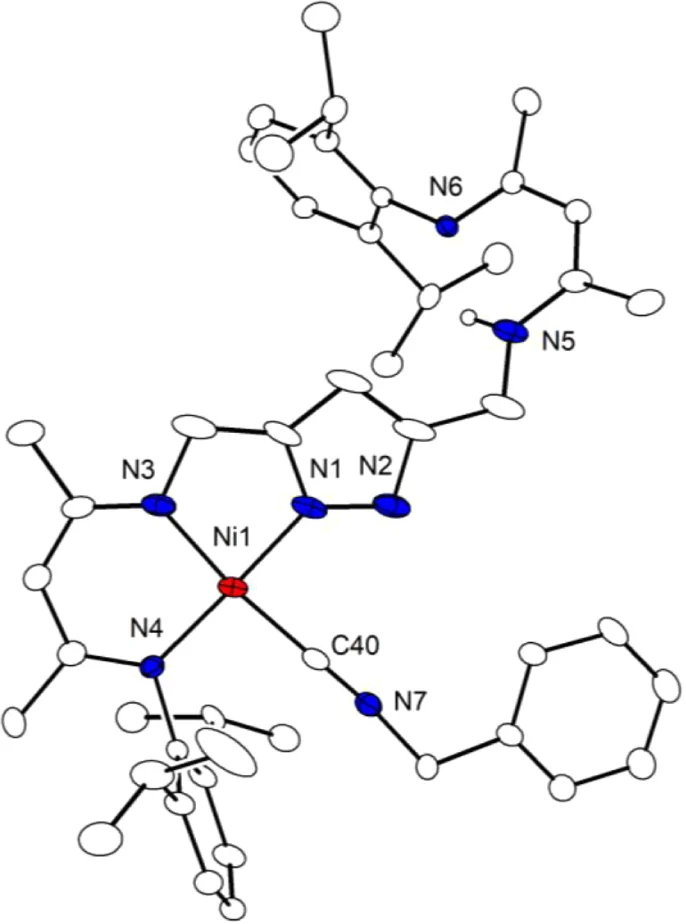
Plot (30% probability thermal ellipsoids) of the molecular structure of **2^CNBn^** (most hydrogen atoms and disorder omitted for clarity). Selected bond lengths [Å] and angles [°]: Ni1–C40 1.831(7), Ni1–N1 1.845(5), Ni1–N3 1.870(6), Ni1–N4 1.896(5), C40–N7 1.165(9), N5···N6 2.682(7); C40–Ni1–N1 88.9(3), C40–Ni1–N3 172.4(3), N1–Ni1–N3 83.8(3), C40–Ni1–N4 92.5(2), N1–Ni1–N4 177.6(2), N3–Ni1–N4 94.9(2), N7–C40–Ni1 179.4(7), N5–H5···N6 134.3.

### Mechanistic Investigations

Thermochemical aspects of the key step of the title reaction were investigated using correlated ab initio theory via coupled-cluster theory including singles and doubles excitation with perturbative triples, CCSD­(T), within the domain-based local pair natural orbital (DLPNO) approximation on DFT-optimized molecular geometries (r^2^SCAN-3c level). The addition of CO to **1** to form **Int­(A)** with a very long Ni–CO distance (2.512 Å) proceeds without a barrier and is slightly exothermic with Δ_r_ *H* = −2.0 kcal mol^–1^. Due to entropy, however, this step is endergonic by Δ_*r*_ *G* = +7.6 kcal mol^–1^ (Figure 5). We computationally probed alternative structures for a CO adduct of **1**, including ones with bridging μ-CO coordination between the two Ni centers, but none corresponds to a local minimum structure (they all will relax to either **Int­(A)** or **Int­(B)**). The barrier for insertion of CO at Δ^‡^ *G* = +22.5 kcal mol^–1^ via **TS­(A,B)** is within reach at room temperature, and it leads to nucleophilic attack of OH toward the formation of a CO insertion intermediate **Int­(B)** at Δ_*r*_ *G* = +11.4 kcal mol^–1^. A 1,3-hydrogen regioisomer **Int­(C)** was also computed and is located at Δ_*r*_ *G* = +8.5 kcal mol^–1^ (for possible transition state structures connecting **Int­(B)** and **Int­(C),** see SI, Figure S38). **Int­(C)** is reminiscent of the intermediate C_red2_-H^+^ in the [Ni,Fe]-CODH catalytic cycle (Scheme 1) and features an O-protonated metal-bridging carbonite (a two-electron reduced CO_2_ ligand, CO_2_^2–^). Intermediate C_red2_ with a Ni,Fe-bridging carbonite that is C-bound to a four-coordinate (square-planar) nickel ion has indeed been crystallographically characterized.^20,23^ Synthetic nickel carbonite complexes are usually produced via reactions of gaseous CO_2_ with reduced nickel(0) precursors^44–46^ or by deprotonation of formate complexes.^47,48^ Attempts to isolate the isocyanate-derived analogue of **Int­(B)** were performed by reaction of **1** with 1 equiv of CNBn. ^1^H NMR spectroscopy analysis revealed partial conversion and low selectivity, while in the ESI mass spectrum two main peaks at *m*/*z* = 856.4 and *m*/*z* = 973.4 can be assigned to ions [**1**+CNBn+H]^+^ and [**1**+2CNBn+H]^+^ (Figures S36–37). Despite all efforts, the isolation of such a product as crystalline material was not successful.

**Figure 5:**
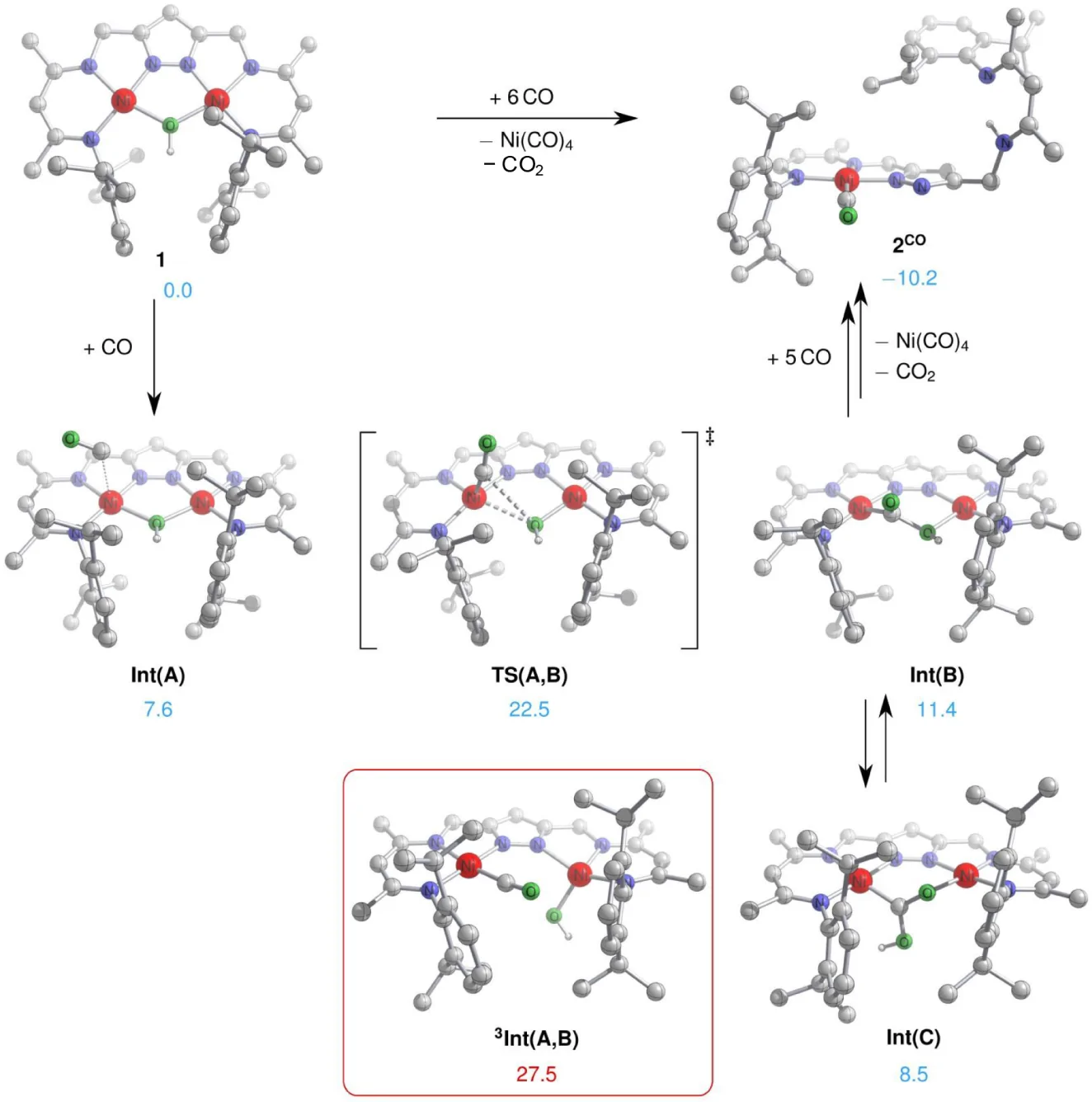
Selected intermediates for the reaction of **1** with CO. Relative Gibbs energies computed at the KS-DLPNO–CCSD­(T1)//r^2^SCAN-3c level are given in kcal mol^–1^.

All of the above species derived from **1** and CO have a singlet ground state (Table S2), and no intermediates with separate Ni1–OH and Ni2–CO subunits are located on the singlet surface. Instead, a corresponding open-shell species, ^**3**^ **Int­(A,B)**, was identified on the triplet surface, which is 5 kcal mol^–1^ above the related singlet **TS­(A,B)**.

The irreversible formation of **2** ^**CO**^ from **Int­(B)** coupled with the release of CO_2_ and Ni­(CO)_4_ is the driving force of the overall reaction from **1** to **2** ^**CO**^, which is clearly exothermic (Δ_*r*_ *H* = −46.4 kcal mol^–1^) and also exergonic at room temperature (Δ_*r*_ *G* = −10.2 kcal mol^–1^). This finding is supported by the fact that the time required for the completion of the reaction varied according to the pressure of CO in the reaction vessel and that no intermediates such as **Int­(B)** or **Int­(C)** could be observed.

Another quasi-planar conformer **2a** ^**CO**^ having the two {N_3_} compartments of L^3–^ oriented as in the bimetallic structure of **1**, which is de facto isoenergetic to the twisted conformer **2** ^**CO**^ that is also found in **2** ^**CNBn**^, was also located on the potential energy surface (Figure 6). The computed frequencies (unscaled) for the C≡O stretching mode of 2153 cm^–1^ and 2141 cm^–1^ as well as for the C=N stretching mode of 1539 cm^–1^ and 1537 cm^–1^ for **2** ^**CO**^ and **2a** ^**CO**^, respectively, are in good agreement with the experimental values recorded for a toluene solution of **2** ^**CO**^. Variable-temperature ^1^H–^1^H NOESY experiments indicate the presence, in solution, of multiple **2** ^**CO**^ conformers. In the NOESY spectrum recorded at 600 MHz at room temperature, all short-distance correlations appear as negative cross-peaks, in agreement with the rapid tumbling of the entire molecule on the Larmor time scale (ω_0_τ_c_ < 1). Notably, NOE correlations of the central pyrazolate-H^4^ proton extend further into the N-protonated side arm (including the dipp substituent), suggesting substantial dangling and bending of this arm (Figure S5). If the temperature is lowered to −25 °C, the NOE correlations in the Ni-bound {N_3_} compartment of L^3–^ become positive due to slowed molecular tumbling (ω_0_τ_c_ > 1); however, the corresponding correlations in the N-protonated side arm remain negative or become weak, indicating enhanced local dynamics (Figure S6). All NMR resonances remain sharp at all temperatures, indicating full conformational flexibility.

**Figure 6:**
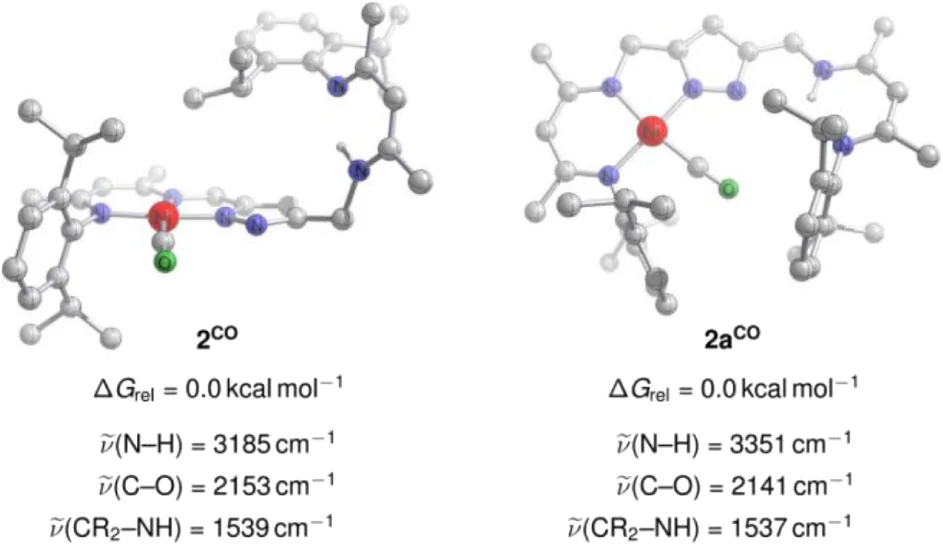
Molecular structures for (isoenergetic) conformers **2^CO^** and **2a^CO^**. Relative Gibbs energies in kcal mol^–1^ and selected stretch mode eigenvalues in cm^–1^ are also given.

## Conclusions

In the present contribution, we demonstrate that in μ-hydroxido dinickel­(II) complex **1**, similar to the crucial C–O bond-forming step at the C-cluster of the [Ni,Fe]-CODH enzymes, two metal ions, one being redox active, cooperate to oxidize CO to CO_2_ via insertion of CO into a Ni–(μ–OH) bond. Moreover, DFT calculations suggest **Int­(A)** and **Int­(B)**, the latter featuring an O-protonated metal-bridging carbonite, as key species for the C–O bond formation and CO_2_ release steps, which mirror the C_red1_-CO and C_red2_-H^+^ states of CODHs, respectively. CO insertion into terminal Ni–OH bonds to give C-bound hydroxycarbonyl species,^9,14,15^ in some cases followed by dimerization yielding C,O-bridging carbonite complexes,^9,14^ has been previously reported and proposed to emulate the [Ni,Fe]-CODH mode of reactivity. At the time, however, it was mostly assumed that the enzyme’s C_red1_ state features a terminal Fe–OH next to a three-coordinate nickel­(II) ion.^21,24^ In light of the revised enzyme mechanism that proposes a metal-bridging hydroxido nucleophile in C_red1_ (Scheme 1),^25^ the present work now shows that CO insertion into a rugged Ni^II^–(μ–OH)–Ni^II^ is also possible.

Oxidation of CO to CO_2_ by **1** is coupled to the loss of one nickel ion as Ni­(CO)_4_ and the formation of a monometallic Ni^II^ complex **2** ^**CO**^. Hence, different from [Ni,Fe]-CODH, where the Ni,Fe pair is amalgamated with a Fe_3_S_4_ cluster fragment and this entire C cluster serves as a robust redox reservoir, one of the Ni^II^ ions is reduced, and the formed Ni­(CO)_4_ serves as an electron sink in the case of **1**. All products of the reaction have been thoroughly characterized by IR and NMR spectroscopies, and the proposed molecular structure of **2** ^**CO**^ was indirectly supported by replacing the CO ligand with CNBn, leading to the formation of **2** ^**CNBn**^. Mechanistic insights were obtained via DFT calculations, which revealed that the formation of **Int­(A)** and **Int­(B)** is energetically uphill, and that the thermodynamic thrust is provided by the irreversible formation of the final products, CO_2_ and Ni­(CO)_4_. This study highlights the cooperativity of two metal ions in mediating the key C–O bond-forming step for the CO to CO_2_ conversion and in hosting key intermediates relevant to the [Ni,Fe]-CODH mechanism within a bimetallic cleft.

## Experimental Section

### General Considerations and Materials

Manipulations involving air- and moisture-sensitive compounds were conducted under an atmosphere of dried (phosphorus pentoxide on solid support [Sicapent, Merck]) argon or nitrogen using standard Schlenk techniques or in an Ar-filled GS glovebox. Toluene was dried with an MBraun Solvent Purification System (SPS) and stored over molecular sieves (3 Å). Pentane was dried over sodium, distilled, and stored over molecular sieves (3 Å). Toluene-d_8_ was dried over potassium-sodium alloy and vacuum distilled. Carbon monoxide and [^13^C] carbon monoxide were purchased from Linde and dried by storage over molecular sieves (3 Å) for at least 1 week prior to usage. All reagents used were purchased from commercial suppliers. If not mentioned otherwise, experiments were done at room temperature.

### NMR Spectroscopy

All NMR spectra were recorded on a Bruker Avance HD III 300, Bruker Avance Neo 400, Bruker Avance HD III 500 NMR spectrometer, or an Avance Neo 600. The ^1^H and ^13^C­{^1^H} NMR spectra were calibrated against the residual ^1^H and natural-abundance ^13^C resonances of the deuterated solvents (toluene-d_8_ δ_H_ = 2.09 ppm; δ_C_ = 137.86 ppm), while ^15^N chemical shifts were indirectly referenced to nitromethane. If not otherwise stated, the spectra were recorded at 25 °C. NMR experiments of air- and moisture-sensitive compounds were conducted in J. Young NMR tubes.

### IR Spectroscopy

IR spectra of solids were recorded on an Agilent Technologies Cary 630 FTIR spectrometer with Dial Path Technology and analyzed with FTIR MicroLab software. Solution IR spectra were acquired on a Bruker Vertex 70 spectrometer using an OTTLE cell with KBr windows and evaluated with the software OPUS 7.8. IR gas-phase spectra were recorded using a custom-made Schlenk gas-phase IR cell (10 cm length, 3.4 cm diameter) with KBr windows. The positions of the bands are given in wavenumbers (cm^–1^), and the following abbreviations were applied for the intensity of bands: s = strong, m = medium, w = weak.

### Mass Spectrometry

HR-ESI-MS were measured with Bruker maXis and Bruker micrOTOF instruments by the Zentrale Massenabteilung at the Georg-August-Universität Göttingen.

### Gas Chromatography

Gas chromatography data were collected on a Shimadzu GC-2014 with a Thermal Conductivity Detector, a molecular sieves column (5 Å 80/100), and He carrier gas.

### UV/Vis Spectroscopy

UV–vis spectra were recorded with an Agilent Cary 60 spectrometer in 1 cm path quartz Schlenk cuvette.

### Elemental Analyses

Elemental analyses were performed by the Analytical Laboratory of the Institute of Inorganic Chemistry at the Georg-August University of Göttingen, using an Elementar Vario EL III instrument.

### Synthetic Procedures

*LNi*_2_ *OH (* **1** *).* LNi_2_Br (475.2 mg, 0.59 mmol, 1.0 equiv) was suspended in THF (30 mL). An aqueous solution of KOH (76.3 mg, 1.36 mmol, 2.3 eq, dissolved in 1 mL of water) was added to the suspension. Stirring the mixture for 4 days at room temperature yielded a dark green solution, which was filtered. The solvent was removed, and the residue was transferred in a glovebox. Crystallization by layering hexanes onto a THF solution of the redissolved residue at room temperature yielded dark green cubes of **1** (256.4 mg, 0.35 mmol, yield 58%). Spectroscopic and analytical data match those reported previously.^28 1^H NMR (400 MHz, toluene-d_8_) δ 6.93–6.89 (m, 2H, arom-H), 6.83–6.81 (m, 4H, arom-H), 5.34 (s, 1H, 1-H), 4.65 (s, 2H, 4-H), 3.65 (sept, *J* = 7.0 Hz, 4H; 6-H), 3.49 (s, 4H, 2-H), 1.75 (d, *J* = 7.0 Hz, 12H, 7-H), 1.40 (s, 6H, 3-H), 1.20–1.17 (s+d, 6H+12H, 5-H+7-H). Elemental analysis calcd. for C_39_H_54_N_6_Ni_2_O: C, 63.28; H, 7.35; N, 11.35. Found: C, 63.25; H, 7.40; N, 11.36.

*HLNiCO (* **2** ^***CO***^). A Schlenk flask was charged with **1** (20.6 mg, 27.8 μmol, 1 equiv) and a stirring bar in a glovebox. The crystalline material was dissolved in dry toluene (5 mL). The flask was transferred outside the glovebox and connected via a three-way valve to a Schlenk line and to a flask containing dry CO. The solution was frozen, and the flasks were evacuated. An excess of dry CO (1 atm) was added to the frozen solution of **1** via vacuum transfer. The flask was brought to room temperature, and the solution was stirred until the color changed from olive green to sage green. The reaction time depends on the CO pressure and can vary from overnight up to generally 2 days and even up to 7 days in the case of very low CO pressure in the flask. Due to the high solubility of **2** ^**CO**^, isolation as solid material was not possible. A spectroscopic yield of 100% was calculated by performing the reaction of **1** with CO in a J. Young NMR tube in the presence of hexamethyldisiloxane as an internal standard (see SI for more details). ^1^H NMR (600 MHz, toluene-d_8_) δ 11.31 (t, *J* = 5.8 Hz, 1H, 12-H), 7.15 (d, *J* = 7.8 Hz, 2H, *m*-H), 7.07–7.05 (m, 1H, *p*-H), 7.00–6.99 (m, 1H, *p*′-H), 6.92 (d, *J* = 7.7 Hz, 2H, *m*′-H), 6.07 (s, 1H, 1-H), 4.81 (s, 1H, 6′-H), 4.69 (s, 1H, 6-H), 4.43 (d, *J* = 5.8 Hz, 2H, 3-H), 3.87 (s, 2H, 3′-H), 3.46 (sept, *J* = 6.9 Hz, 2H, 10′-H), 3.18 (sept, *J* = 6.9 Hz, 2H; 10-H), 1.79 (s, 3H, 5-H), 1.67 (s, 3H, 8H), 1.51 (s, 3H, 5′-H), 1.44–1.40 (m, 9H, 8́-H+11′-H), 1.24 (d, *J* = 6.9 Hz, 6H, 11-H), 1.21 (d, *J* = 6.9 Hz, 6H, 11-H), 1.05 (d, *J* = 7.0 Hz, 6H, 11′-H). ^13^C­{^1^H} NMR (126 MHz, toluene-d_8_) δ 176.35 (13-C), 166.62 (7-C), 162.24 (4′-C), 159.93 (7′-C), 156.50 (4-C), 155.13 (9′-C), 154.62 (2-C), 151.83 (2′-C), 148.43 (9-C), 141.98 (*o*′-C), 138.64 (*o*-C), 124.68 (*m*′-C), 123.44 (*m*-C), 98.56 (6′-C), 95.91 (1-C), 94.17 (6-C), 55.04 (3′-C), 42.84 (3-C), 28.93 (10-C), 28.70 (10′-C), 24.65 (11-C), 24.40 (11′-C), 24.21 (11′-C), 23.45 (11-C), 22.06 (8-C), 21.99 (8′-C), 21.94 (5′-C), 19.66 (5-C). ^15^N NMR (60 MHz, toluene-d8) δ −67.2 (2C–N), −103.2 (7C–N), −164.9 (2’C–N), −210.0 (4′C–N), −233.5 (7′C–N), −274.3 (4C–N). ESI-MS (toluene, *m*/*z*): 665.4 [**2**+H]^+^, 609.5 [^**H3**^ **L**+H]^+^. IR in toluene [cm^–1^]: *ṽ* 2118 (s), 1622 (s), 1555 (s), 1396 (s), 1319 (m), 1256 (w), 1225 (w), 1182 (w), 1101 (w), 935 (w), 804 (m), 787 (m), 760 (s). UV–vis (*c* = 1.7 × 10^–3^ M in THF) [nm, (L·mol^–1^·cm^–1^)]: λ, (*ε*) 500 (222), 580 (167), 655 (85).

*HLNi­(CNBn) (* **2** ^***CNBn***^). To a solution of **2** ^**CO**^ (26.4 mg, 38.0 μmol, 1.0 equiv), neat CNBn was added (4.6 μL, 38.0 μmol, 1.0 equiv); note that the amount of **2** ^**CO**^ was estimated by the weighing difference of an empty vial and the same vial with in situ prepared **2** ^**CO**^ after removing the solvent. The solution immediately turned from dark green/red to bright orange/red, and gas evolution was observed. The solvent was removed, and the residue was redissolved in toluene. Crystallization by diffusing pentane into the toluene solution at −35 °C gave crystals suitable for XRD analysis over 2 weeks (10.4 mg, 13.3 μmol, yield = 35.0%). ^1^H NMR (600 MHz, toluene-d_8_) δ 11.38 (t, *J* = 6.0 Hz, 1H, 12-H), 7.24–7.21 (m, 2H, 16-H), 7.15 (d, *J* = 7.6 Hz, 2H, Ar–H), 7.09–7.05 (m, 3H, Ar–H), 7.00–6.98 (m, 1H, Ar–H), 6.90 (m, 3H, 17-H+18-H), 6.07 (s, 1H, 1-H), 4.86 (s, 1H, 6′-H), 4.72 (d, *J* = 0.7 Hz, 1H, 6-H), 4.54 (d, *J* = 6.0 Hz, 2H, 3-H), 3.97 (s, 2H, 3′-H), 3.75 (s, 2H, 14-H), 3.65 (sept, *J* = 6.9 Hz, 2H, 10′-H), 3.18 (sept, *J* = 6.9 Hz, 2H, 10-H), 1.89 (d, *J* = 0.6 Hz, 3H, 5-H), 1.69 (s, 3H, 8-H), 1.59 (s, 3H, 5′-H), 1.48 (s, 3H, 8′-H), 1.36 (d, *J* = 6.9 Hz, 6H, 11′-H), 1.23 (d, *J* = 6.9 Hz, 6H, 11-H), 1.19 (d, *J* = 6.9 Hz, 6H, 11-H), 1.11 (d, *J* = 6.9 Hz, 6H, 11′-H). ^13^C­{^1^H} NMR (126 MHz, toluene-d_8_) δ 166.57 (7-C), 161.83 (4′-C), 159.15 (7′-C), 156.43 (4-C), 154.25 (9′-C), 153.16 (2-C), 152.17 (2′-C), 148.49 (9-C), 142.61 (*o*’-C), 138.70 (o-C), 135.25 (13-C), 132.19 (15-C), 127.91 (16-C), 126.45 (17-C/18-C), 123.95 (17-C/18-C), 123.47 (*m*’-C), 123.41 (*m*-C), 98.27 (6′-C), 95.35 (1-C), 94.06 (6-C), 54.14 (3′-C), 47.06 (14-C), 43.20 (3-C), 28.95 (10-C), 28.65 (10′-C), 24.63 (11-C), 24.40 (11′-C), 24.32 (11′-C), 23.48 (11-C), 22.95 (8-C), 22.10 (8-C), 21.95 (5′-C), 19.82 (5-C). ^**15**^ **N NMR** (50 MHz, toluene-d_8_) δ −272.6 (4C–N), −235.9 (7́C–N), −215.1 (4′C–N), −209.9 (CNBn), −163.1 (2’C–N), −103.0 (7C–N), −67.6 (2C–N). ESI-MS (toluene, *m*/*z*): 782.4 [**2** ^**CNBn**^+H]^+^, 665.5 [**2**+H]^+^. IR [cm^–1^]: *ṽ* 3061 (w), 3029 (w), 2956 (w), 2923 (w), 1319 (m), 2865 (w), 2218 (s), 1622 (s), 1555 (s), 1529 (s), 1497 (w), 1463 (m), 1456 (m), 1435 (s), 1398 (s), 1380 (m), 1361 (m), 1350 (w), 1318 (s), 1294 (w), 1279 (w), 1251 (w), 1212 (w), 1178 (w), 1096 (w), 1055 (w), 1033 (w), 1020 (w), 986 (w), 958 (w), 934 (w), 904 (w), 803 (m), 790 (m), 766 (m), 738 (s), 697 (s). UV–vis (c = 2.5 × 10^–3^ M in THF) [nm, (L·mol^–1^·cm^–1^)]: λ, (*ε*) 526 (123).

*(HL)*_2_ *Ni*_3_ *(OH)*_2_ *(TBD) (* **3** ^***TBD***^). A solution of **1** in toluene (6.9 mg, 9.3 μmol, 1.0 equiv) was added to TBD (1.3 mg, 9.3 μmol, 1.0 equiv). The solution was transferred into a Schlenk flask, and dry CO (1 atm) was added in the same manner as during the synthesis of **2** ^**CO**^. The reaction mixture was brought to room temperature, and a color change from olive green to brown/yellow is observed within a few minutes. The reaction mixture was stirred for 4 days at room temperature. Crystallization by diffusing pentane into the THF solution at room temperature gave a few crystals of **3** ^**TBD**^; because of the presence of the hydroxido bridges, we assume **3** ^**TBD**^ is formed by a side reaction with residual water (yield not determined). ESI (THF, *m*/*z*): 1560.8 [**3** ^**TBD**^+H]^+^, 1421.8 [**3**+H]^+^, 1405.7 [(**3**–H_2_O)+H]^+^, 710.9 [**3**+H]^2+^.

### X-ray Crystallography

Crystallographic details for **2** ^**CNBn**^ and **3** ^**TBD**^ can be found in Section 1 of the Supporting Information: crystal data and details of the data collections are given in Tables S1. The molecular structures are shown in Figures S25 and S27. X-ray data were collected on a BRUKER D8-QUEST diffractometer (monochromated Mo-Kα radiation, λ = 0.71073 Å) using ω and φ scans at low temperature. The structure was solved with SHELXT and refined on *F* ^2^ using all reflections with SHELXL.^49,50^ Non-hydrogen atoms were refined anisotropically. Hydrogen atoms were placed in calculated positions and assigned an isotropic displacement parameter of 1.5/1.2 *U*_eq_(C/N). In **2** ^**CNBn**^, a pentane molecule was found to be disordered about a 2-fold rotation axis and was refined at 1/2 occupancy using DFIX (*d*(C–C) = 1.55 Å), SADI (*d*(C···C)) and RIGU restraints. A disordered ^i^Pr group (C19/20/21/A/B, occupancy factors 0.59(4)/0.41(4)) was refined using SAME and SADI restraints. In **3** ^**TBD**^, the oxygen-bound hydrogen atom in **3** ^**TBD**^ was refined freely with a fixed isotropic displacement parameter of 0.08 Å^2^. TBD in **3** ^**TBD**^ was found to be disordered about a 2-fold rotation axis and was refined at 1/2 occupancy using RIGU restraints. Absorption corrections were performed by the multi-scan method with SADABS.^51^

### Computational Details

Geometry optimizations and harmonic frequency calculations were performed with the ORCA^52,53^ program package, employing Grimme’s r^2^SCAN-3c^53^ composite scheme, which makes use of the meta-generalized-gradient approximation (mGGA) and a specifically tailored valence triple-ζ basis set (mTZVPP),^54^ the D4 London dispersion correction,^55^ and a geometrical counterpoise (gCP) correction^56^ to account for inter- and intramolecular basis set superposition errors (BSSE). The transition structure was verified by eigenvalue analysis of the Hessian, and connectivities between minima and the corresponding TS were validated by intrinsic reaction coordinate (IRC)^57^ following calculations.

Relative energies were produced using correlated coupled-cluster theory within the domain-based local-pair natural orbital framework, including single and double excitations with iterative perturbative triple contributions, i.e., DLPNO–CCSD­(T1).^58,59^ A DFT-based Kohn–Sham (KS) orbital reference wave function using the PBE0 ^60,61^ functional was chosen. Restricted and unrestricted KS reference wave functions were used for closed-shell singlet and high-spin open-shell species, respectively. The NormalPNO setting was applied. The one-particle space on hydrogen atoms was described with the def2-SVP basis set, all other nonmetal atoms (C, N, O) with the def2-TZVP sets, and the Ni metal centers with the def2-TZVPP and def2-QZVPP sets. Two-point extrapolations to both the complete pair-natural-orbital (PNO) space (CPS) limit and the complete basis set (CBS) limit for Ni were performed, resulting in KS-DLPNO–CCSD­(T1)/CPS/CBS­(Ni = T,Q) energies according to the following extrapolation schemes:^62–64^

Two-point PNO extrapolation of correlation energies is computed for the def2-TZVPP basis set on Ni as

$$E_{\mathrm{corr}}^{\mathrm{PNO}}=E_{\mathrm{corr}}^{\mathrm{loose}}+F\times \left(E_{\mathrm{corr}}^{\mathrm{tight}}-E_{\mathrm{corr}}^{\mathrm{loose}}\right)$$

where *F* = 2.38 for loose (1.00 × 10^–6^) and *tight* (3.33 × 10^–7^) TCutPNO thresholds.

Two-point CBS extrapolations of SCF reference and correlation energies are computed as

$$E_{\mathrm{ref}}^{\mathrm{CBS}}=\frac{E_{\mathrm{ref}}^{X}\exp\left(-\alpha \sqrt{Y}\right)-E_{\mathrm{ref}}^{Y}\exp\left(-\alpha \sqrt{X}\right)}{\exp\left(-\alpha \sqrt{Y}\right)-\exp\left(-\alpha \sqrt{X}\right)}$$

$$E_{\mathrm{corr}}^{\mathrm{CBS}}=\frac{X^{\beta }E_{\mathrm{corr}}^{X}-Y^{\beta }E_{\mathrm{corr}}^{Y}}{X^{\beta }-Y^{\beta }}$$

with cardinal numbers X = 3 and Y = 4 for T and Q, and α = 7.88, β = 2.97 for the def2-family. The CBS extrapolation for the correlation energies is applied to results with the NormalPNO settings (TCutPNO = 3.33 × 10^–7^) to yield an additive correction term, $\delta E_{\mathrm{corr}}^{\mathrm{CBS}}$ , which is defined as

$$\delta E_{\mathrm{corr}}^{\mathrm{CBS}}=E_{\mathrm{corr}}^{\mathrm{CBS}}-E_{\mathrm{corr}}^{X}$$

The final extrapolated energies are obtained as

$$E^{\mathrm{CPS}/\mathrm{CBS}\left(\mathrm{Ni}=T,Q\right)}=E_{\mathrm{ref}}^{\mathrm{CBS}}+E_{\mathrm{corr}}^{\mathrm{PNO}}+\delta E_{\mathrm{corr}}^{\mathrm{CBS}}$$

Relative enthalpies and Gibbs energies are reported at the extrapolated KS-DLPNO–CCSD­(T1) level and include the corresponding thermal contributions from the r^2^SCAN-3c geometry optimizations, i.e., KS-DLPNO–CCSD­(T1)//r^2^SCAN-3c.

Additional information can be found in Section 4 of the Supporting Information.

## Supplementary Material



## Notes

General experimental details and materials, synthetic procedures, ESI mass spectra, UV/vis spectra, NMR spectra, IR spectra, crystallographic and computational details, including atom coordinates of computationally optimized geometries (PDF)

## #

Sorbonne Université, Institut Parisien de Chimie Moléculaire UMR 8232, 75005 Paris, France
