# Supplementary material for: CO to CO2 Conversion at a Dinickel(II) Complex with Bridging Hydroxide—Emulating a Key Step of [Ni,Fe]-CO Dehydrogenase
Source: Inorg Chem. 2026 Aug 20;65(34):19705–14. doi: 10.1021/acs.inorgchem.6c01897 (PMC13536883; doi:10.1021/acs.inorgchem.6c01897)
Supplement: Supplementary file 1 [file ic6c01897_si_001.pdf]

Supporting Information for

CO to CO_2 Conversion at a Dinickel(II) Complex with Bridging Hydroxide – Emulating a Key Step of [Ni,Fe]-CO Dehydrogenase

Sara I. Mozzi,[a] Martin Diefenbach,[b] Chung-Wei Fu,[a] Sebastian Dechert,[a] Michael John,[a] Vera Krewald,[b] and Franc Meyer[a,c]*

[a] University of Göttingen, Institute of Inorganic Chemistry, Tammannstraße 4, 37077 Göttingen, Germany

[b] TU Darmstadt, Department of Chemistry, Quantum Chemistry, 64287 Darmstadt, Germany

[c] University of Göttingen, International Center for Advanced Studies of Energy Conversion (ICASEC), Tammannstraße 6, 37077 Göttingen, Germany

* Corresponding Author:
E-Mail: franc.meyer@chemie.uni-goettingen.de

Table of Contents

1. X-Ray Crystallography S2
2. Spectroscopic/Analytical Data of Complexes S3
3. Additional Data S22
4. Computational Details S29
5. References S79

1. X-Ray Crystallography

Crystal data and details of the data collections are given in Table S1. The molecular structures are shown in Figure S25 and S27.

Table S1. Crystal data and refinement details for $\mathbf{2^{CNBn}}$ and $\mathbf{3^{TBD}}$.

**compound**	$\mathbf{2^{CNBn}}$ **(sim24_23)**	$\mathbf{3^{TBD}}$ **(sim40_23)**
empirical formula	$C_{49.50}H_{67}N_7Ni$	$C_{85}H_{123}N_{15}Ni_3O_2$
moiety formula	$C_{47}H_{61}N_7Ni$, 0.5(C_5H_{12})	$C_{85}H_{123}N_{15}Ni_3O_2$
formula weight	818.81	1563.11
T [K]	100(2)	100(2)
crystal size [mm³]	0.318 x 0.303 x 0.081	0.197 x 0.170 x 0.080
crystal system	monoclinic	monoclinic
space group	*C*2/*c* (No. 15)	*C*2/*c* (No. 15)
a [Å]	36.277(3)	37.9243(10)
b [Å]	15.8039(12)	9.2518(3)
c [Å]	17.1194(14)	23.4950(7)
α [°]	90	90
β [°]	111.353(2)	97.2180(10)
γ [°]	90	90
V [Å³]	9141.2(12)	8178.3(4)
Z	8	4
ρ [g·cm^{-3}]	1.190	1.270
F(000)	3528	3352
μ [mm^{-1}]	0.465	0.740
T_{min} / T_{max}	0.87 / 0.96	0.89 / 0.94
Θ–range [°]	2.053 – 25.350	2.267 – 27.942
hkl–range	±43, –17 to 19, ±20	±49, ±12, ±30
measured refl.	48604	89748
unique refl. [R_{int}]	8300 [0.0756]	9785 [0.0380]
observed refl. ($I > 2\sigma(I)$)	6402	8870
data / restr. / param.	8300 / 38 / 586	9785 / 75 / 534
goodness–of–fit (F^2)	1.196	1.194
*R*1, *wR*2 ($I > 2\sigma(I)$)	0.1044 / 0.2049	0.0512 / 0.1044
*R*1, *wR*2 (all data)	0.1338 / 0.2184	0.0570 / 0.1074
res. el. dens. [e·Å^{-3}]	–1.346 / 0.900	–0.880 / 0.774

2. Spectroscopic/Analytical Data of Complexes

2.1 Complex $LNi_2(\mu\text{-}OH)$ (1)

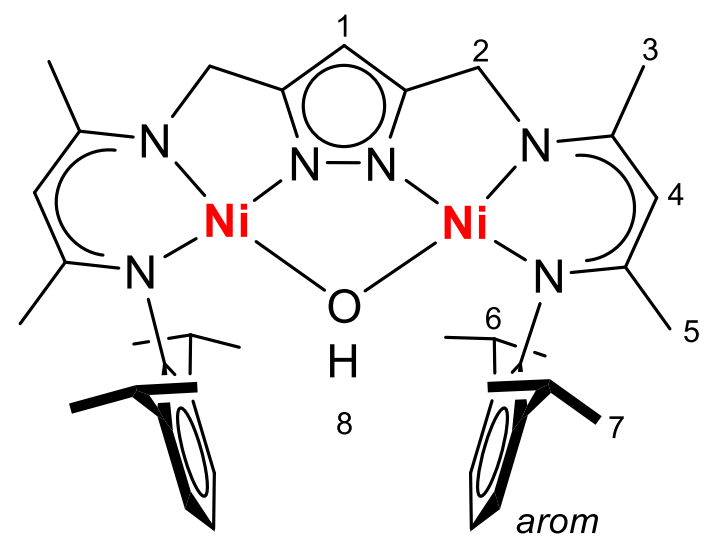

1

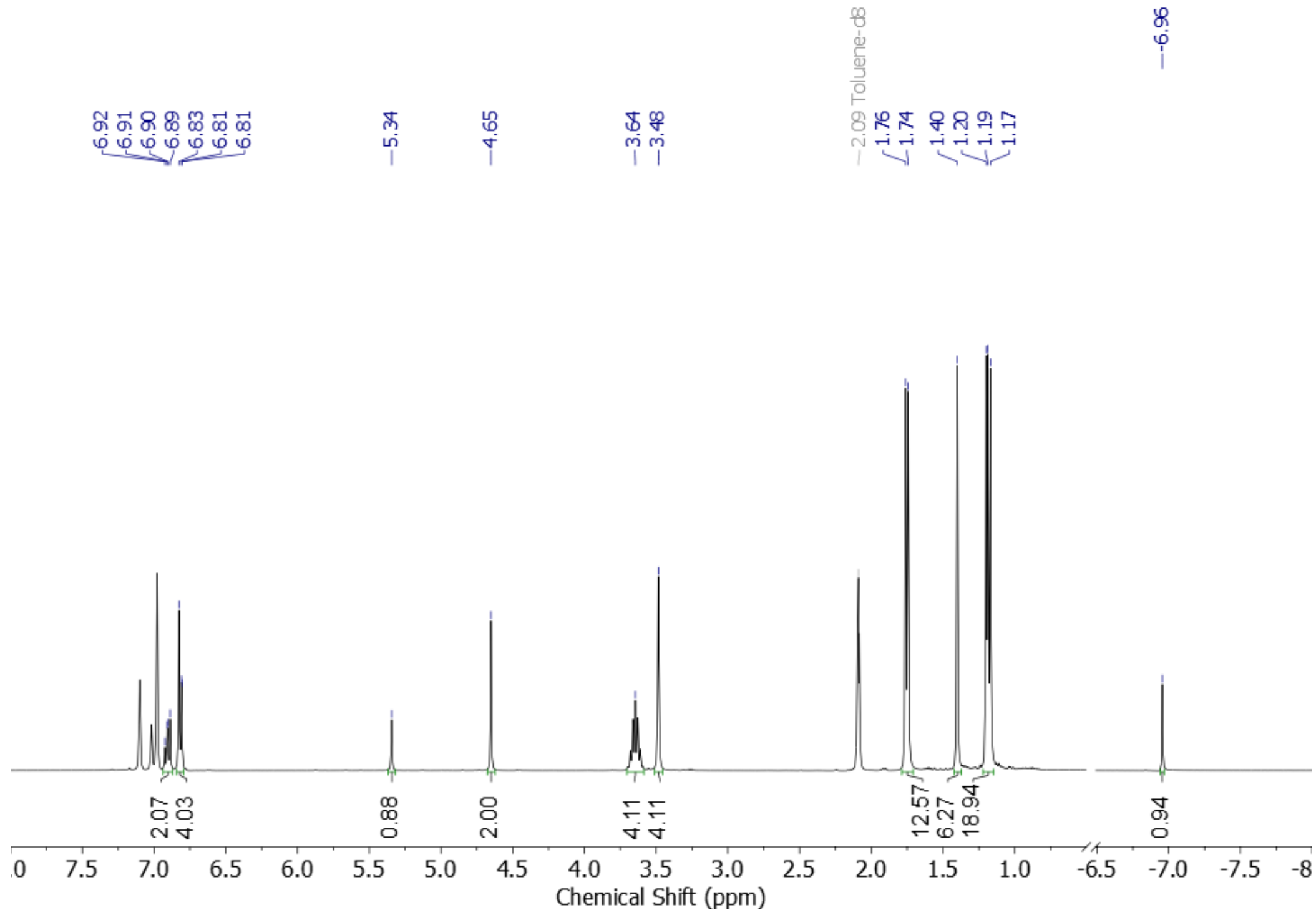

Figure S1: ^{1}H NMR spectrum (400 MHz) of **1** in toluene-d8.

2.2 Complex HLNi(CO) (2^{CO})

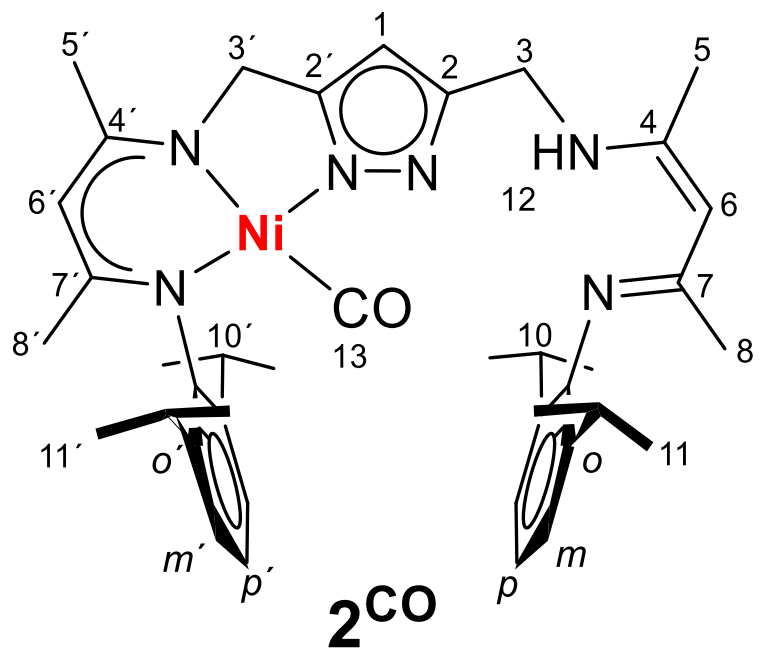

Spectroscopic yield determination of the reaction of 1 with CO

Hexamethyldisiloxane was used as internal standard: a 0.165 M stock solution in toluene-d$_8$ was prepared. 20 µL (3.3 µmol, 1 eq) of the internal standard stock solution were added to a solution of **1** (5.0 mg, 6.8 µmol, 2 eq.) in toluene-d$_8$ in a J. Young NMR-tube. A ^{1}H-NMR spectrum was recorded (Figure S2, top). Dry CO was subsequently added as described in the experimental section. After overnight reaction, a ^{1}H-NMR spectrum was recorded confirming the completion of the reaction (Figure S2, bottom).

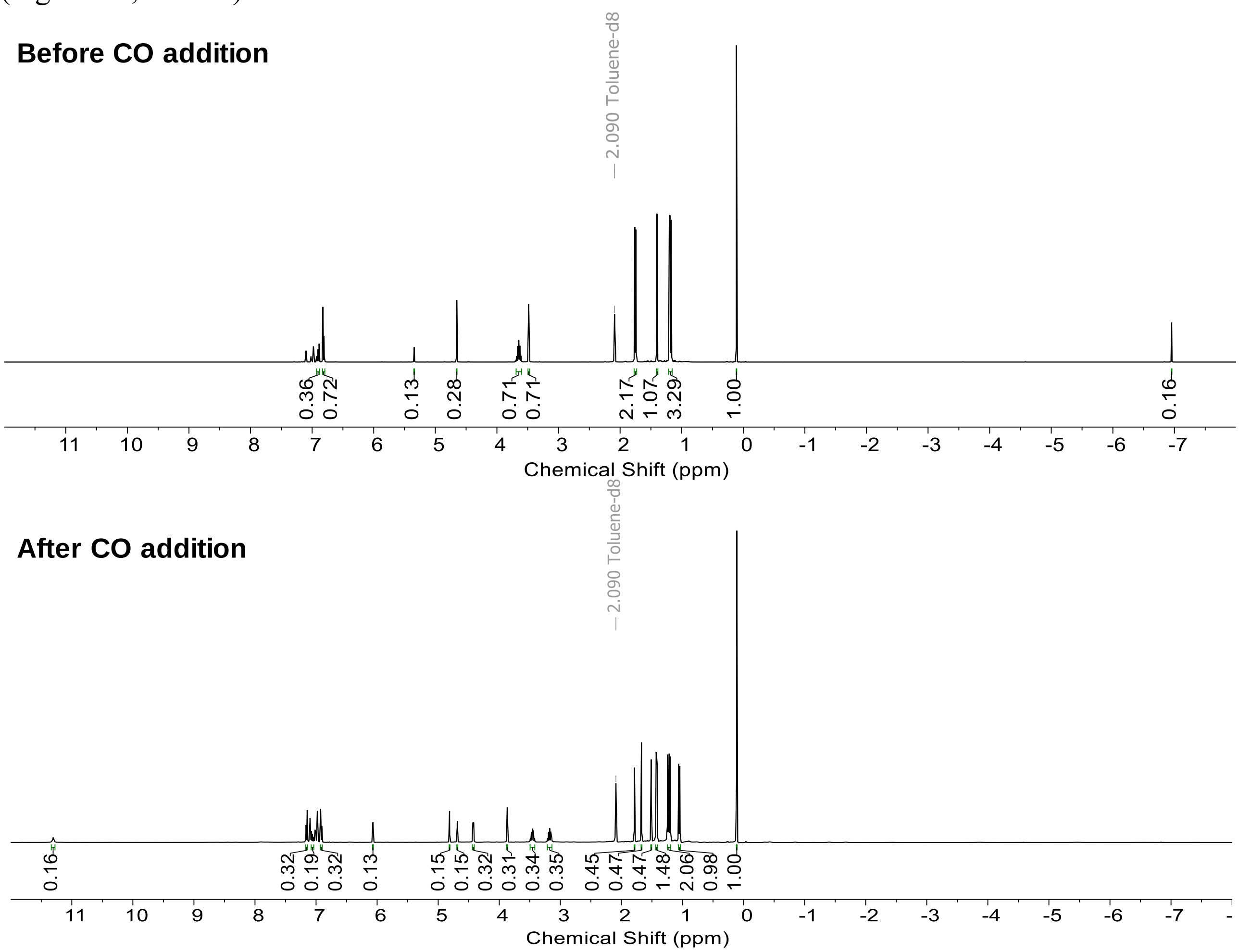

Figure S2: ^{1}H NMR spectrum (400 MHz) of: **1** and internal standard in toluene-d$_8$ (top), same reaction mixture after reaction with CO (**2**$^{\mathbf{CO}}$, bottom).

The integral of the internal standard was set to 1.00 proton in both experiments. The mmol of **1** were calculated according to the formula:

$$mmol_1 = \frac{\frac{I_1}{N_1} \cdot mmol_{std}}{\frac{I_{std}}{N_{std}}} = \frac{\frac{0.13}{1} \cdot 0.0033}{\frac{1}{18}} = 0.0077\ mmol_1$$

Where:

I_1 = integral corresponding to the pyrazolate-H^4 signal of complex **1**.

N_1 = number of the protons represented by the pyrazolate-H^4 signal of complex **1**, equal to 1.

I_{std} = integral corresponding to the internal standard signal.

N_{std} = number of the protons represented by signal of the internal standard, equal to 18.

The mmol of $\mathbf{2^{CO}}$ obtained at the end of the reaction were calculated according to the formula:

$$mmol_{2CO} = \frac{\frac{I_{2CO}}{N_{2CO}} \cdot mmol_{std}}{\frac{I_{std}}{N_{std}}} = \frac{\frac{0.13}{1} \cdot 0.0033}{\frac{1}{18}} = 0.0077\ mmol_{2CO}$$

Where:

I_{2CO} = integral corresponding to the pyrazolate-H^4 signal of complex $\mathbf{2^{CO}}$.

N_{2CO} = number of the protons represented by the pyrazolate-H^4 signal of complex $\mathbf{2^{CO}}$, equal to 1.

I_{std} = integral corresponding to the internal standard signal.

N_{std} = number of the protons represented by signal of the internal standard, equal to 18.

Assuming the theoretical amount of $\mathbf{2^{CO}}$ equal to the initial mmol of **1**, the spectroscopic yield is calculated as:

$$Yield\ \% = \frac{mmol_{2CO}}{mmol_1} \cdot 100 = \frac{0.0077}{0.0077} \cdot 100 = 100\%$$

NMR Spectra

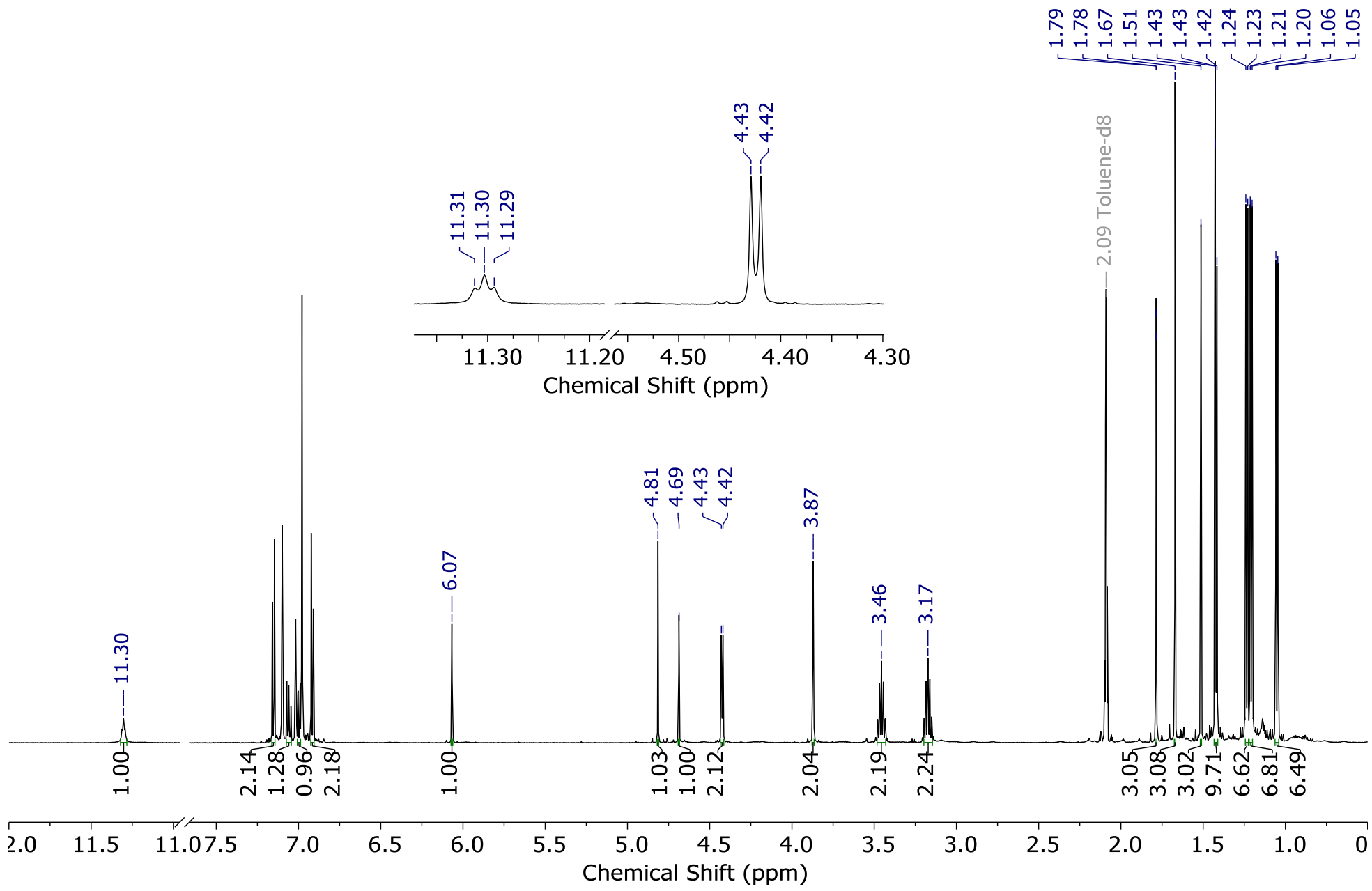

Figure S3: ^{1}H NMR spectrum of **2**$^{\mathbf{CO}}$ (^{13}CO labelled) in toluene-d$_8$. Characterization after reaction of **1** with ^{13}CO.

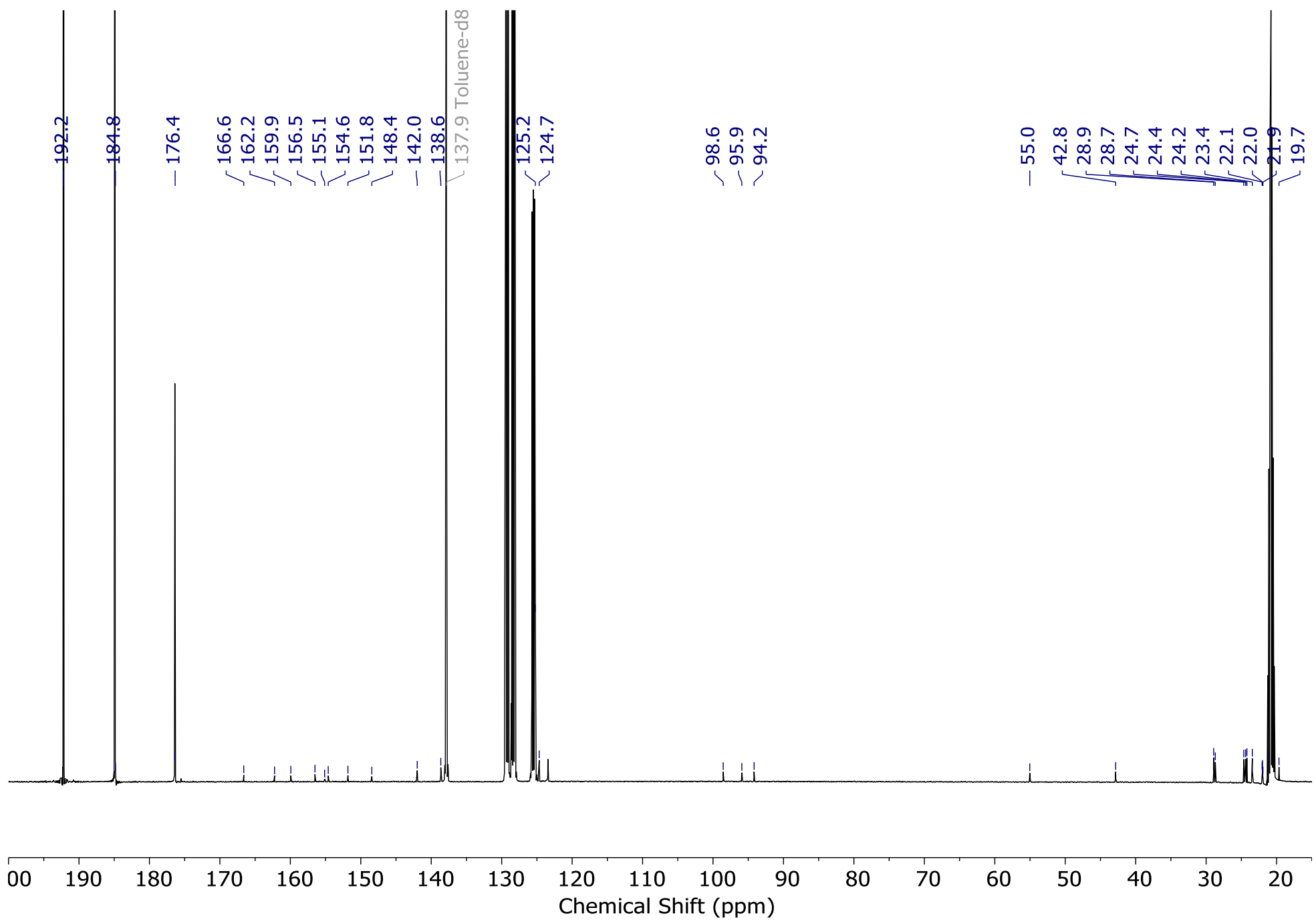

Figure S4: ^{13}C{^{1}H} NMR spectrum of **2**$^{\mathbf{CO}}$ (^{13}CO labelled) in toluene-d$_8$. Characterization after reaction of **1** with ^{13}CO: $Ni(CO)_4$ at 192.2 ppm, CO at 184.8 ppm and CO_2 at 124.7 ppm.

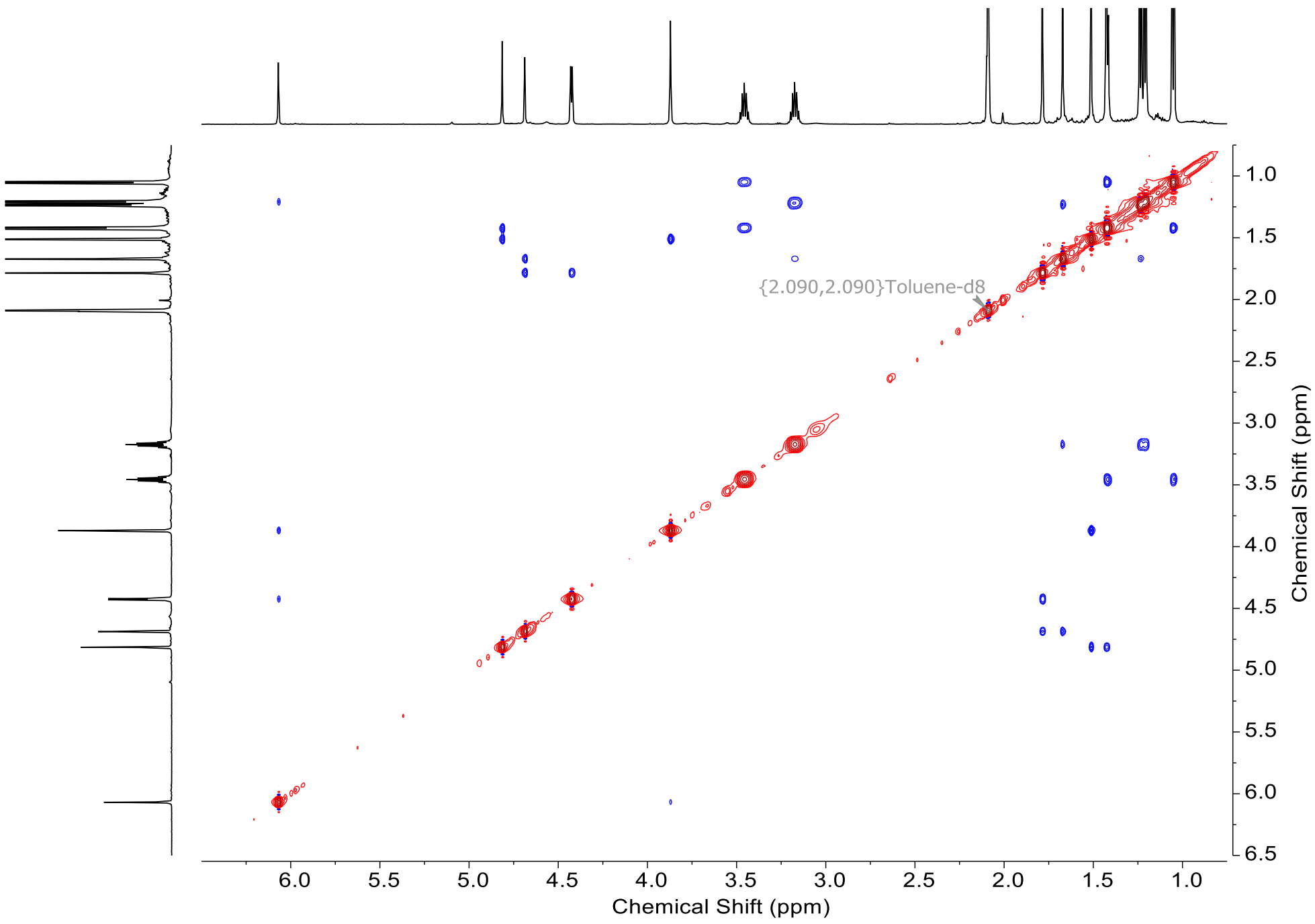

Figure S5: Section of the $^{1}H-^{1}H$ NOESY spectrum of $\mathbf{2}^{CO}$ in toluene-d$_8$, recorded at 25 °C. The experiment was performed after reaction of **1** with CO.

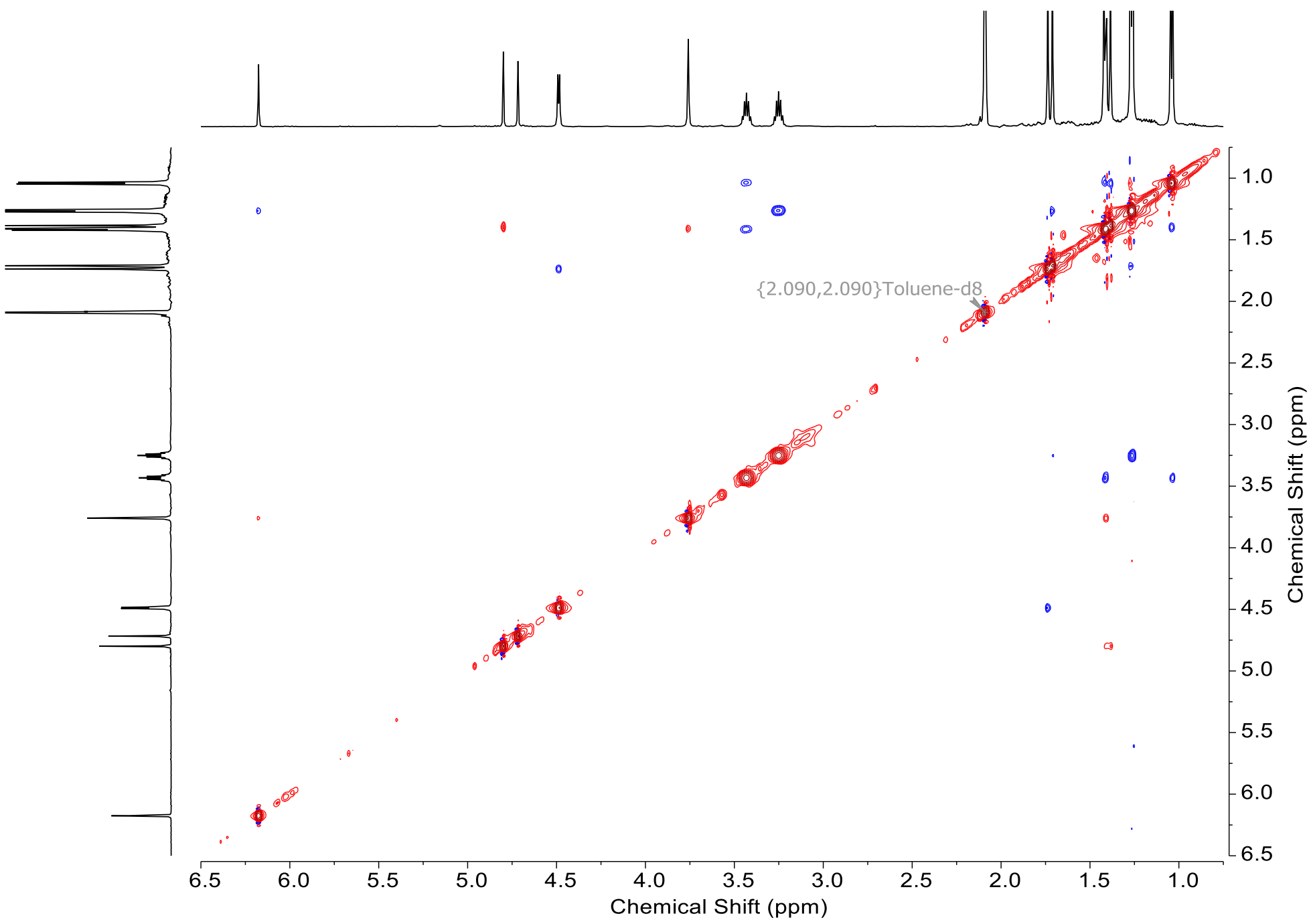

Figure S6: Section of the $^{1}H-^{1}H$ NOESY spectrum of $\mathbf{2}^{CO}$ in toluene-d$_8$, recorded at −25 °C. The experiment was performed after reaction of **1** with CO.

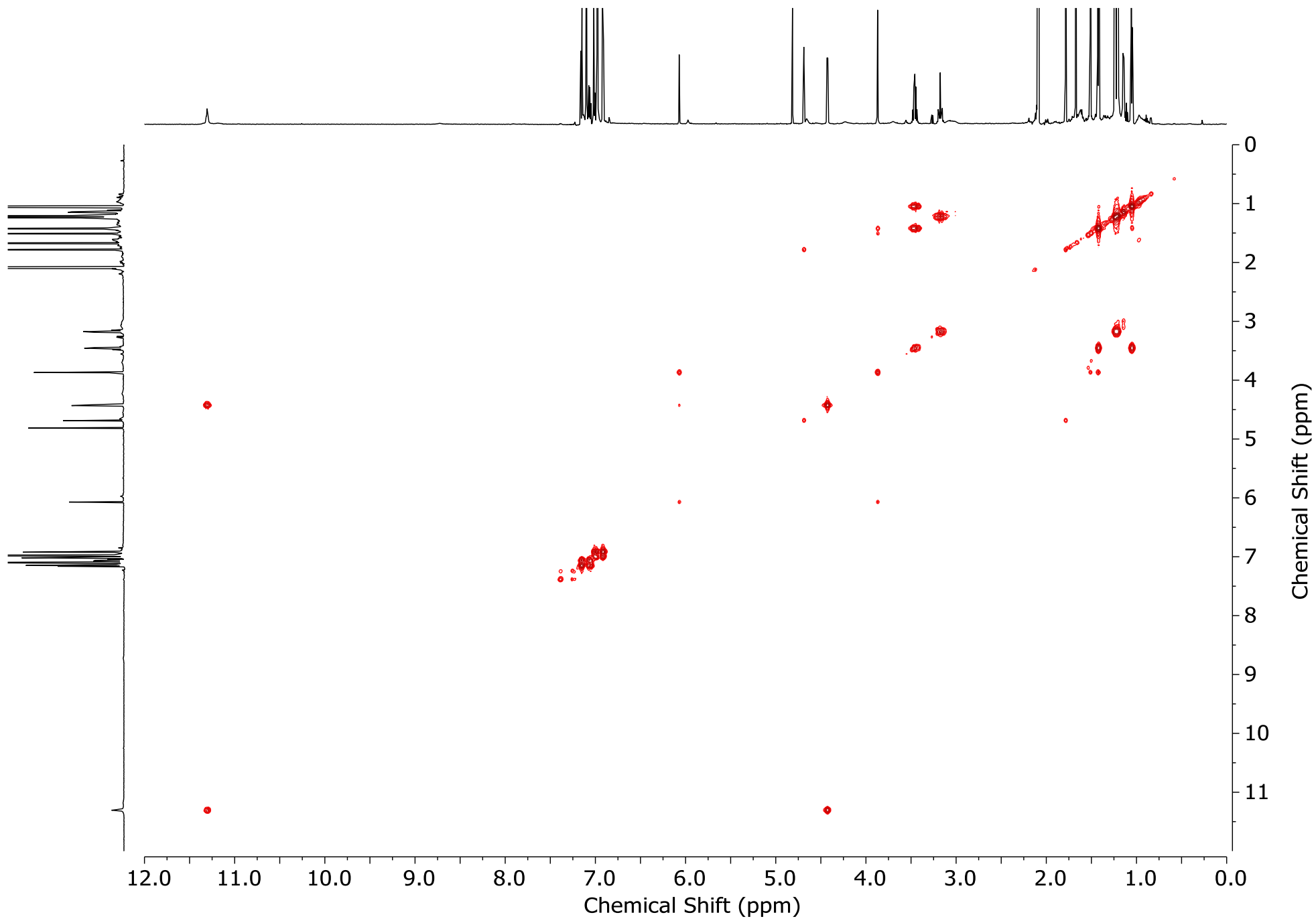

Figure S7: ^{1}H–^{1}H COSY NMR spectrum of **2^{CO}** (^{13}CO labelled) in toluene-d_8. The spectrum was recorded after the reaction of **1** with ^{13}CO.

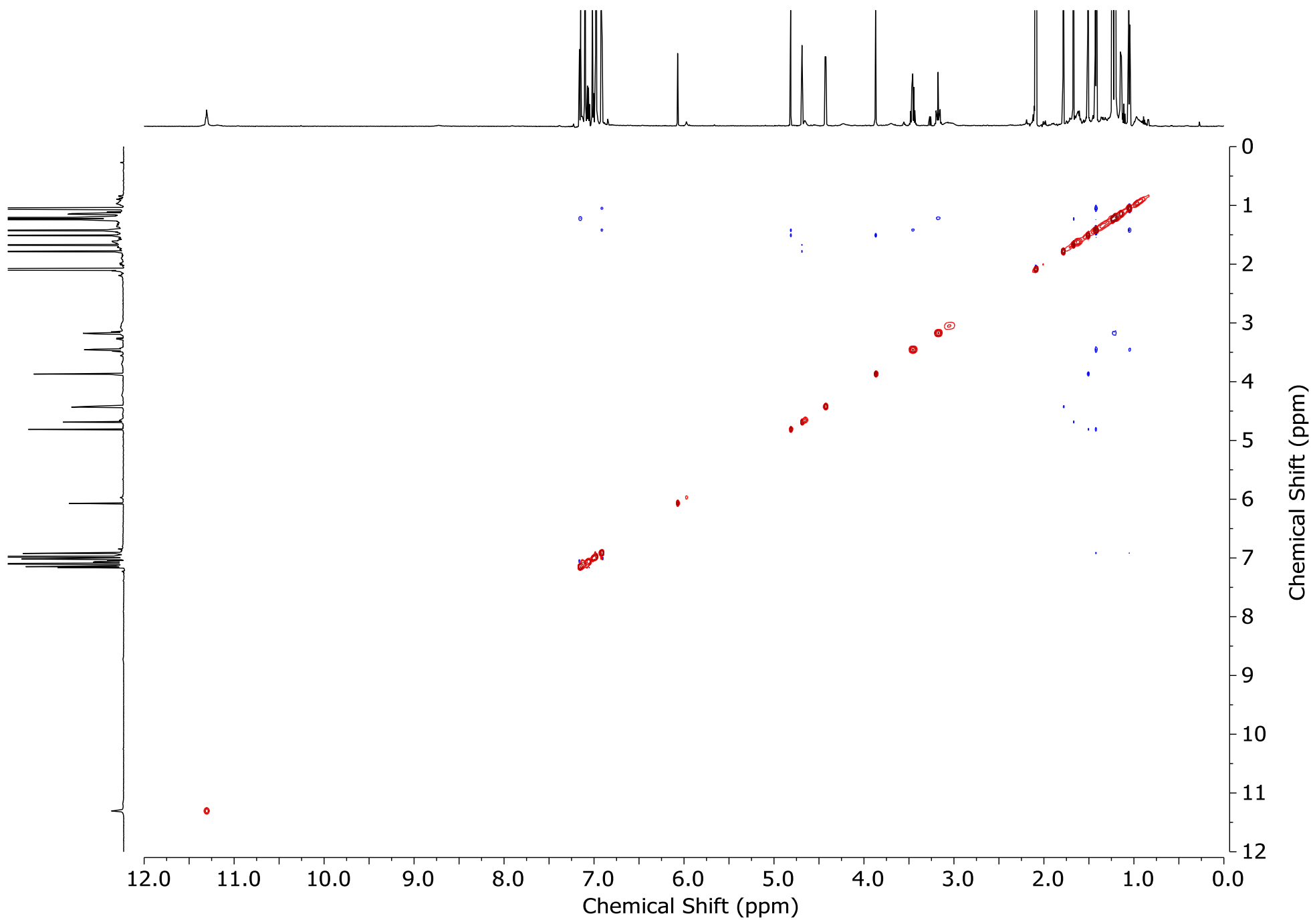

Figure S8: ^{1}H–^{1}H NOESY NMR spectrum of **2^{CO}** (^{13}CO labelled) in toluene-d_8. The spectrum was recorded after the reaction of **1** with ^{13}CO.

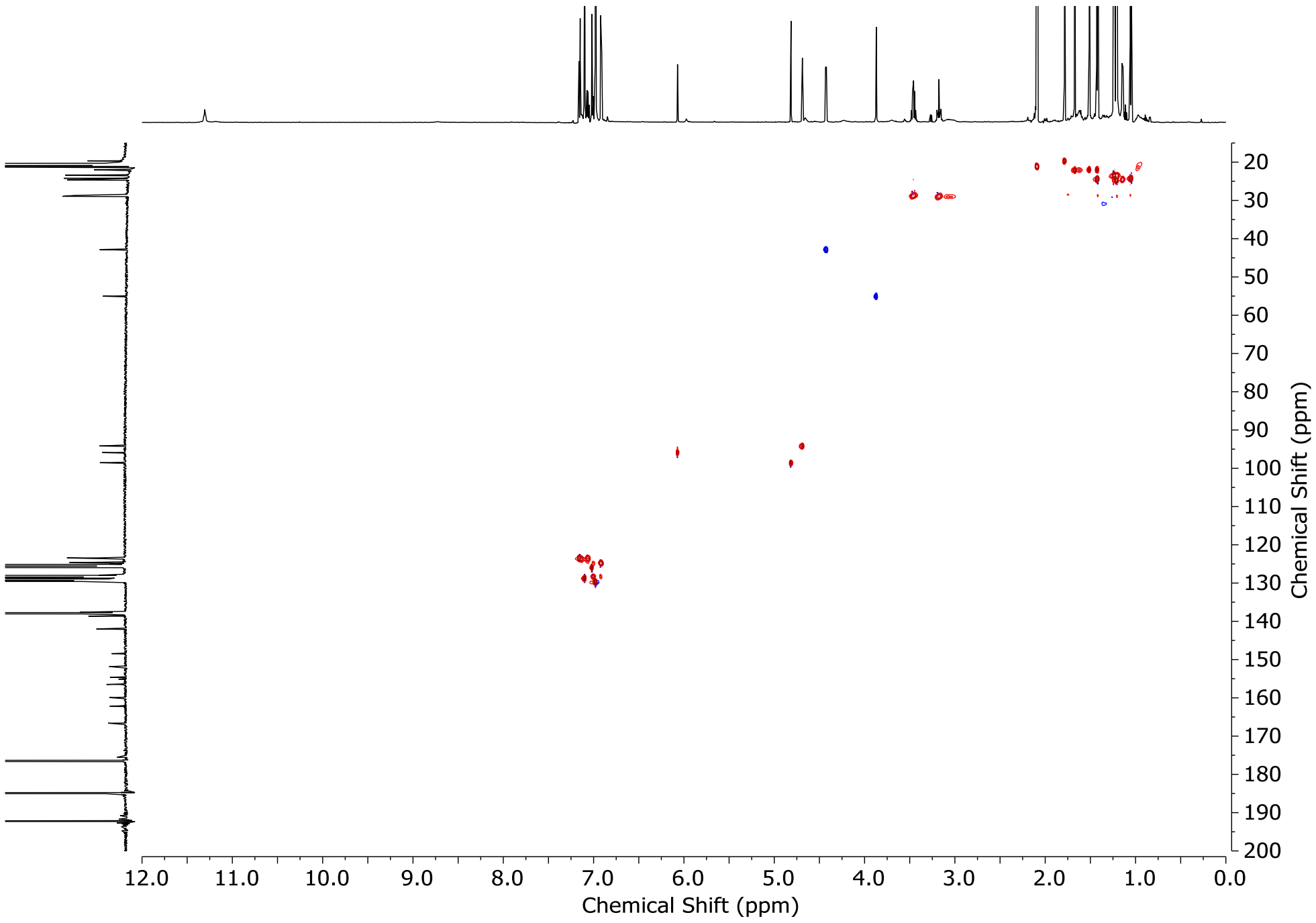

Figure S9: $^{1}H–^{13}C$ HSQC NMR spectrum of $\mathbf{2}^{CO}$ (^{13}CO labelled) in toluene-d_8. The spectrum was recorded after the reaction of **1** with ^{13}CO.

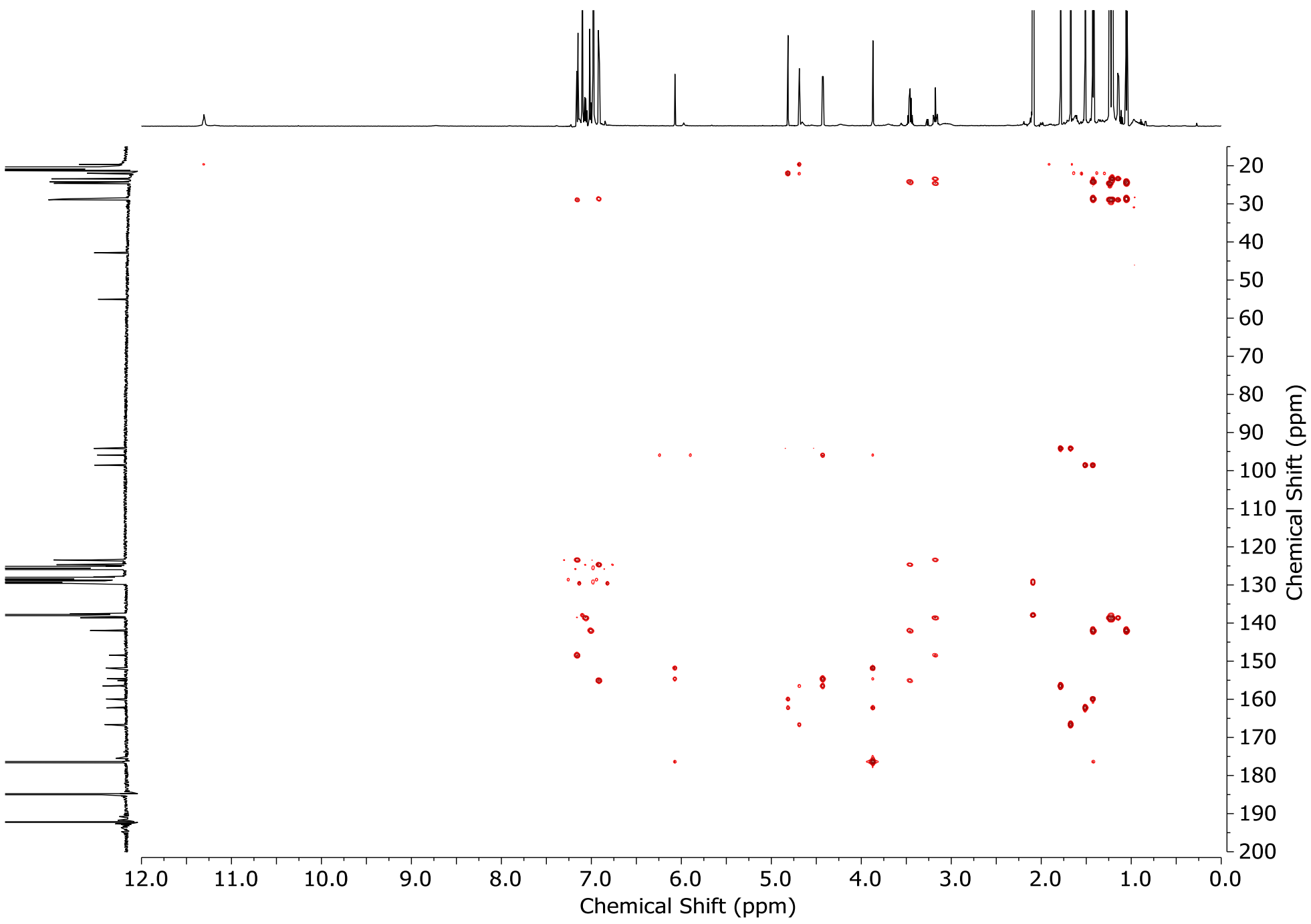

Figure S10: $^{1}H–^{13}C$ HMBC NMR spectrum of $\mathbf{2}^{CO}$ (^{13}CO labelled) in toluene-d_8. The spectrum was recorded after the reaction of **1** with ^{13}CO.

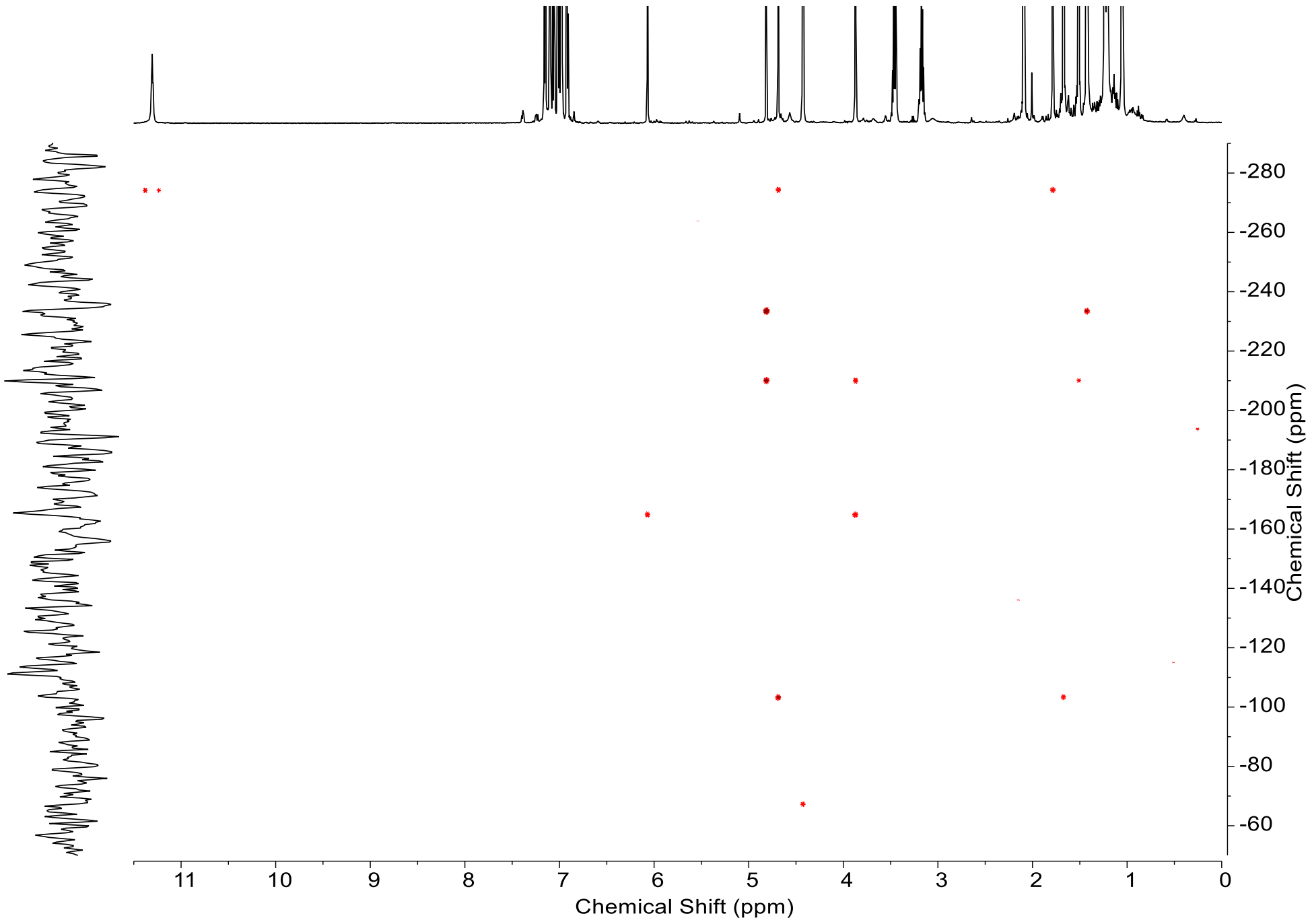

Figure S11: ^{1}H–^{15}N HMBC NMR spectrum of **2**$^{\mathbf{CO}}$ (^{13}CO labelled) in toluene-d_8. The spectrum was recorded after the reaction of **1** with ^{13}CO.

ESI Mass Spectrometry Data

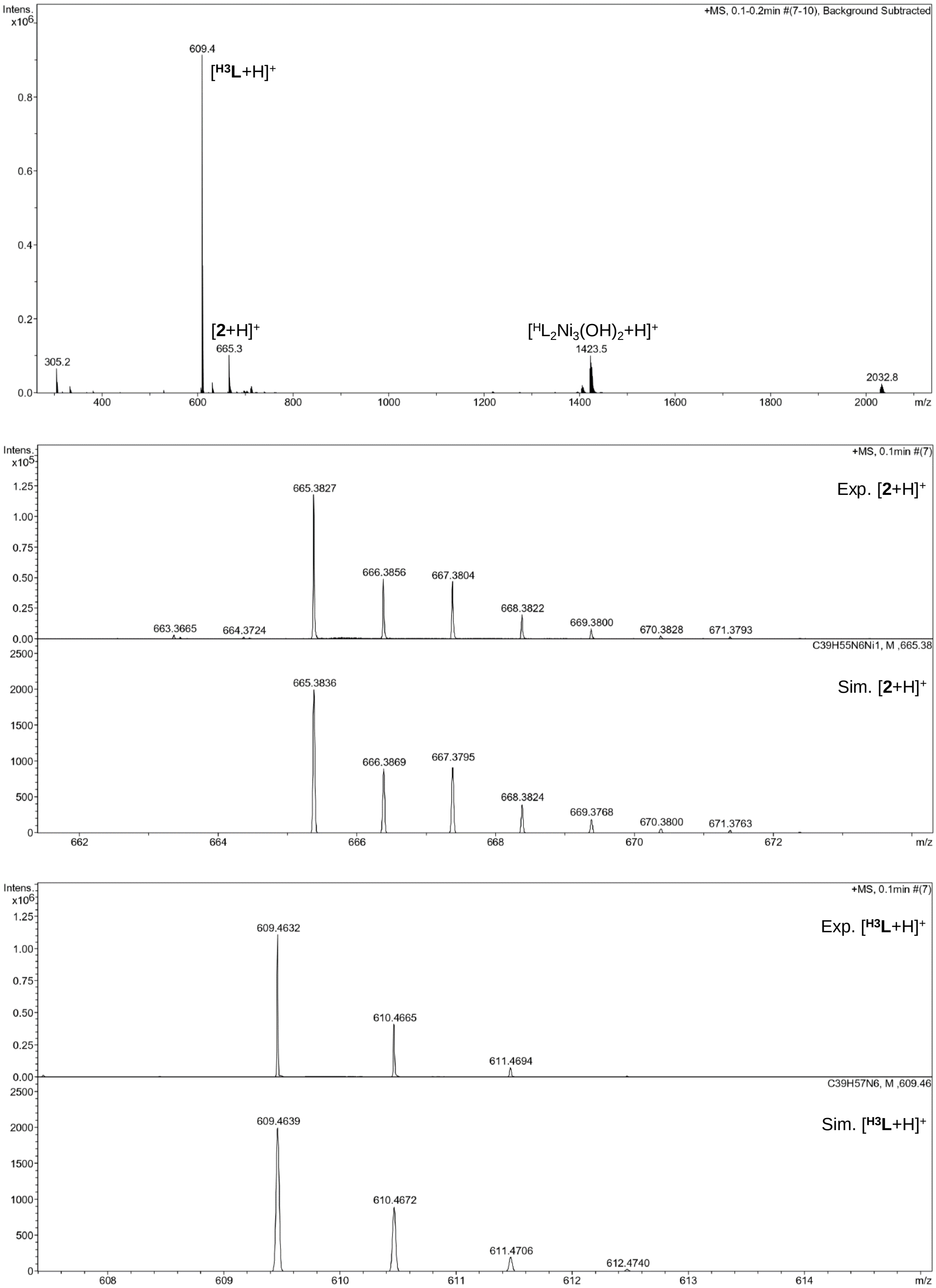

Figure S12: Top: ESI mass spectrum of 2^{CO} in THF. Middle: section of the spectrum showing the peak corresponding to $[HLNi+H]^+$ at m/z = 665.3 and simulated isotopic pattern. Bottom: section of the spectrum showing the peak corresponding to and $[H_3L+H]^+$ at m/z = 609.5 and simulated isotopic pattern.

IR and UV-Vis Spectroscopic Data

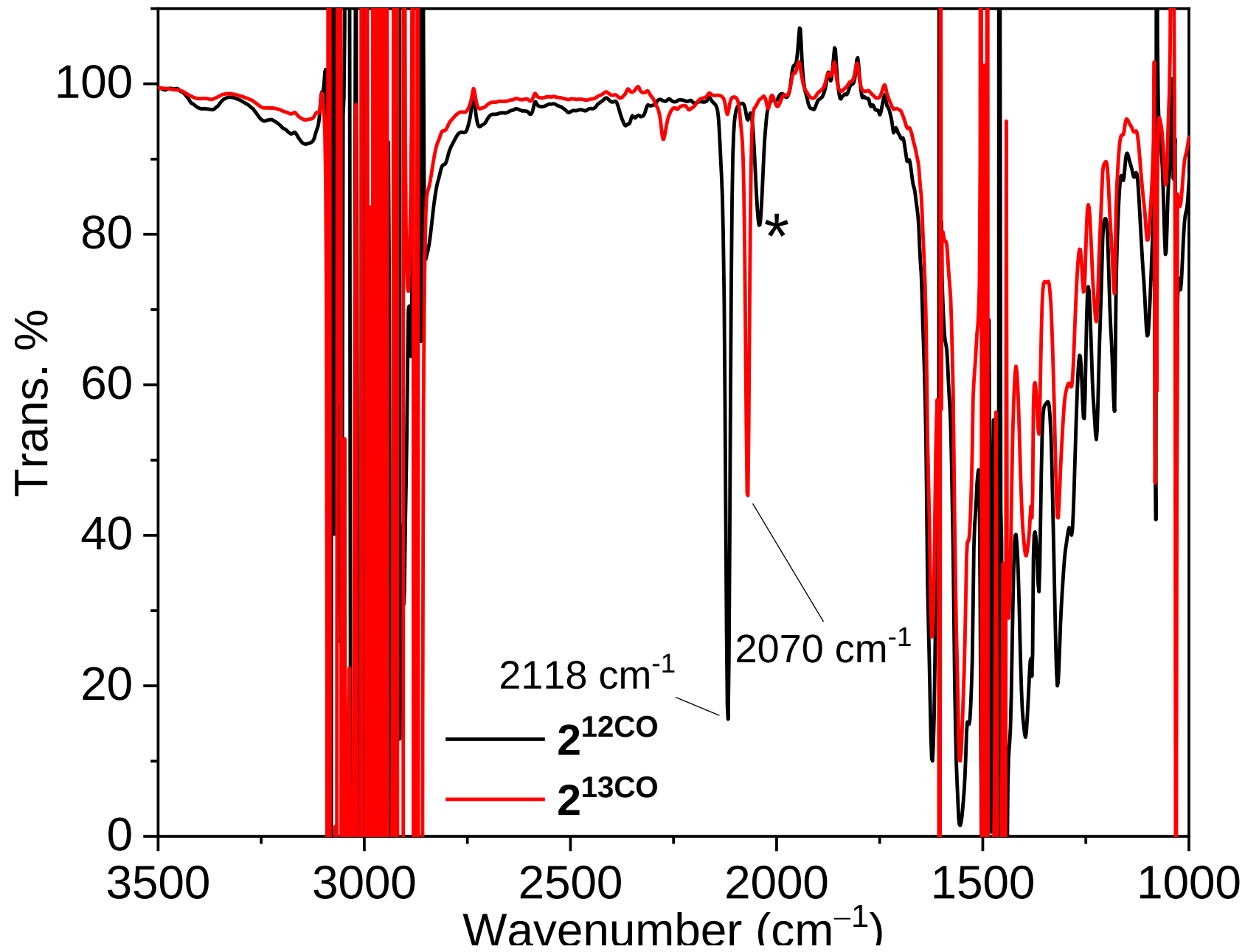

Figure S13: IR spectrum of toluene solutions of $\mathbf{2^{CO}}$ (black) and ^{13}CO labelled $\mathbf{2^{CO}}$ (red). Residual $Ni(CO)_4$ is marked with an asterisk.

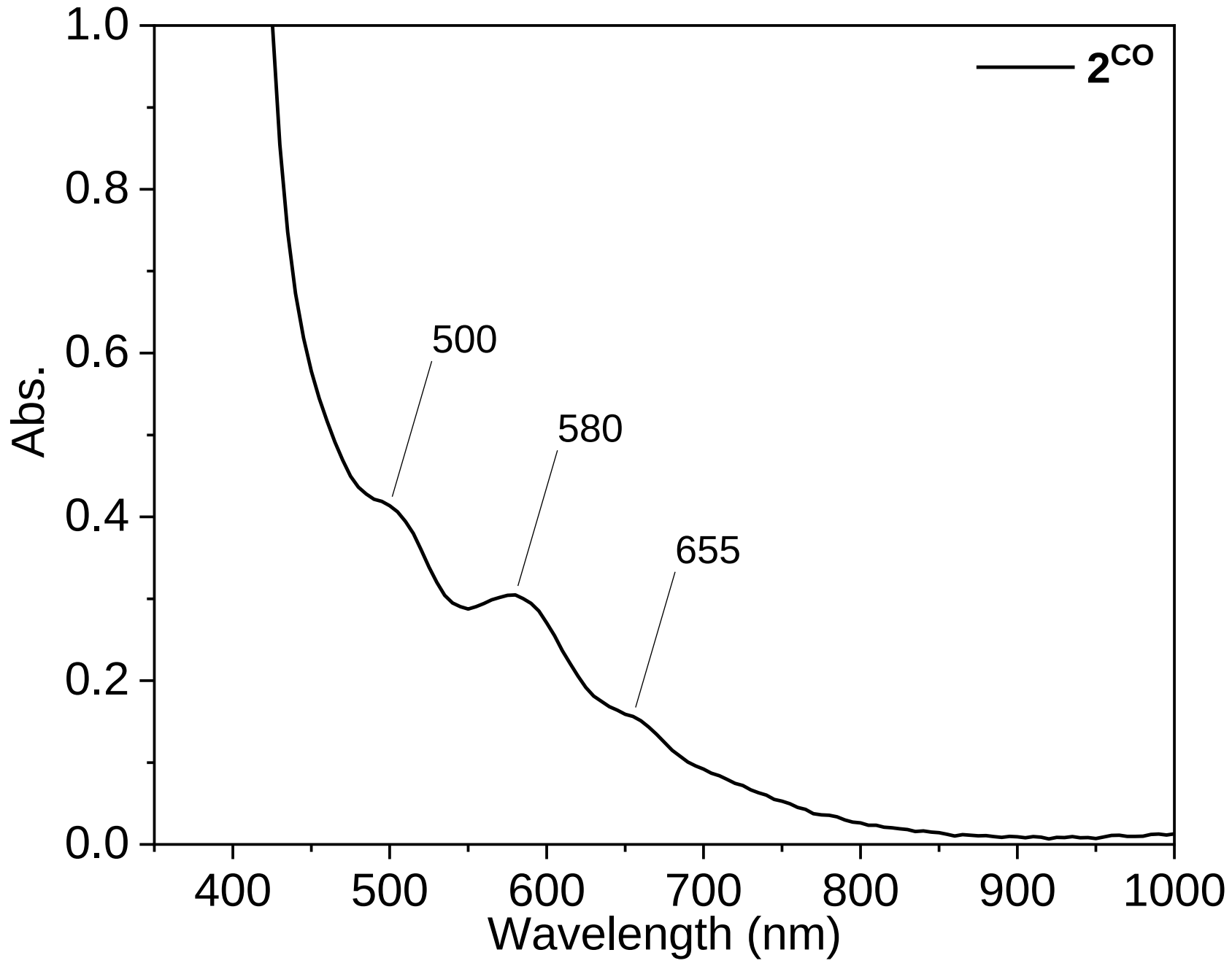

Figure S14: UV-vis spectrum of $\mathbf{2^{CO}}$ in toluene (c = 1.7×10^{-3} M).

2.3 Complex HLNi(CNBn) (2^{CNBn})

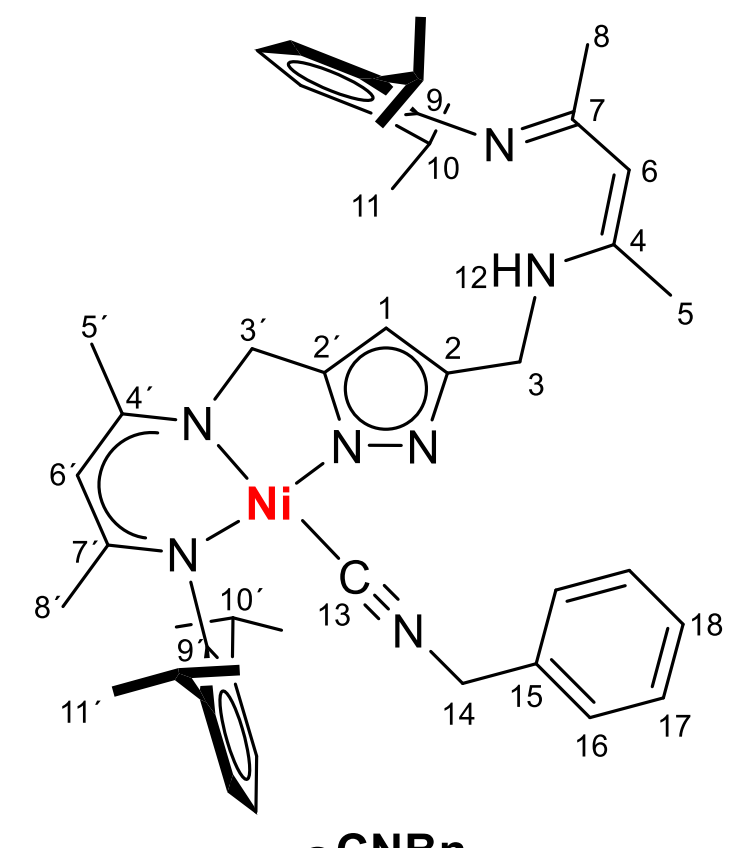

2^{CNBn}

NMR Spectra

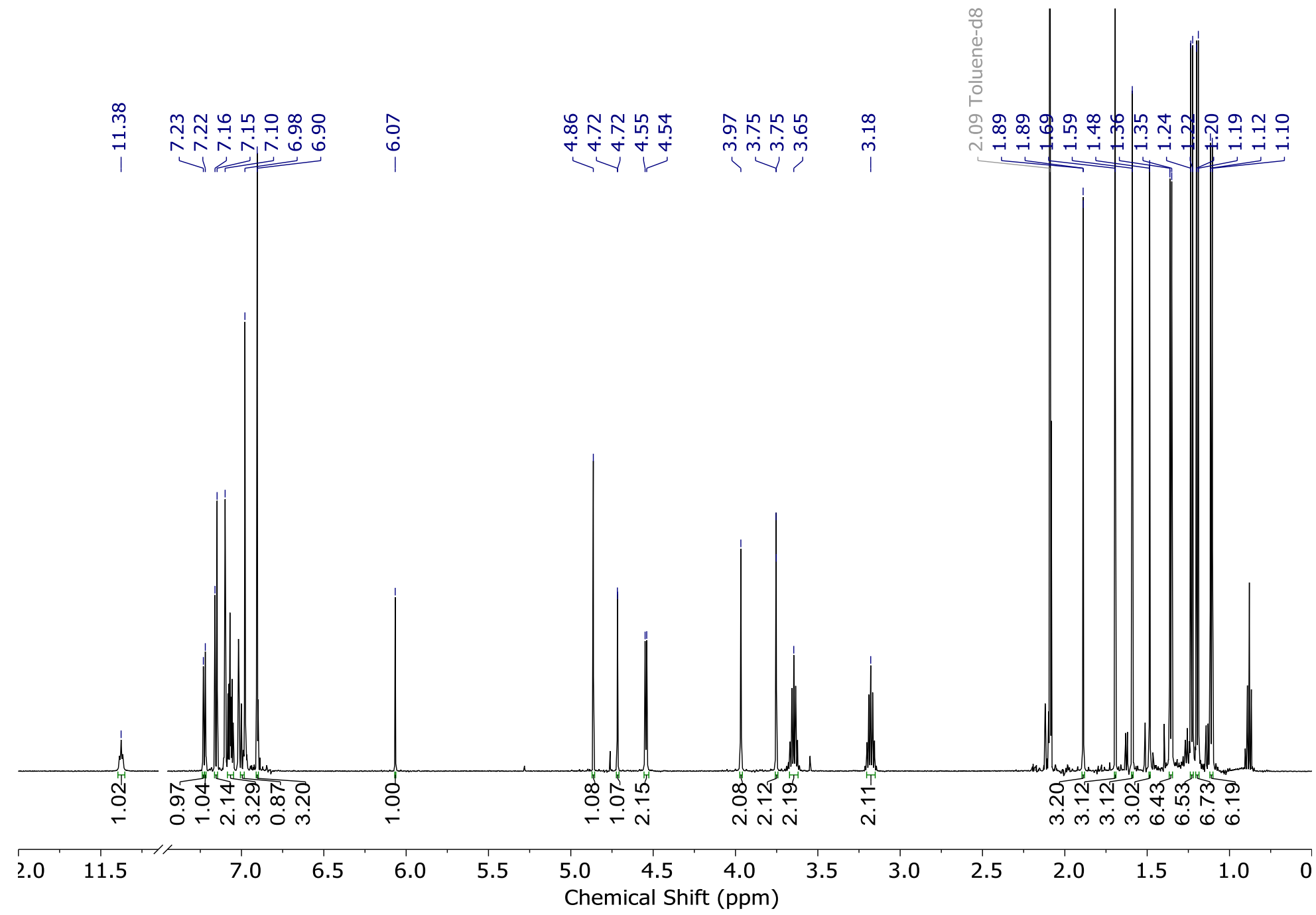

Figure S15: ^{1}H NMR spectrum of $\mathbf{2^{CNBn}}$ in toluene-d_8.

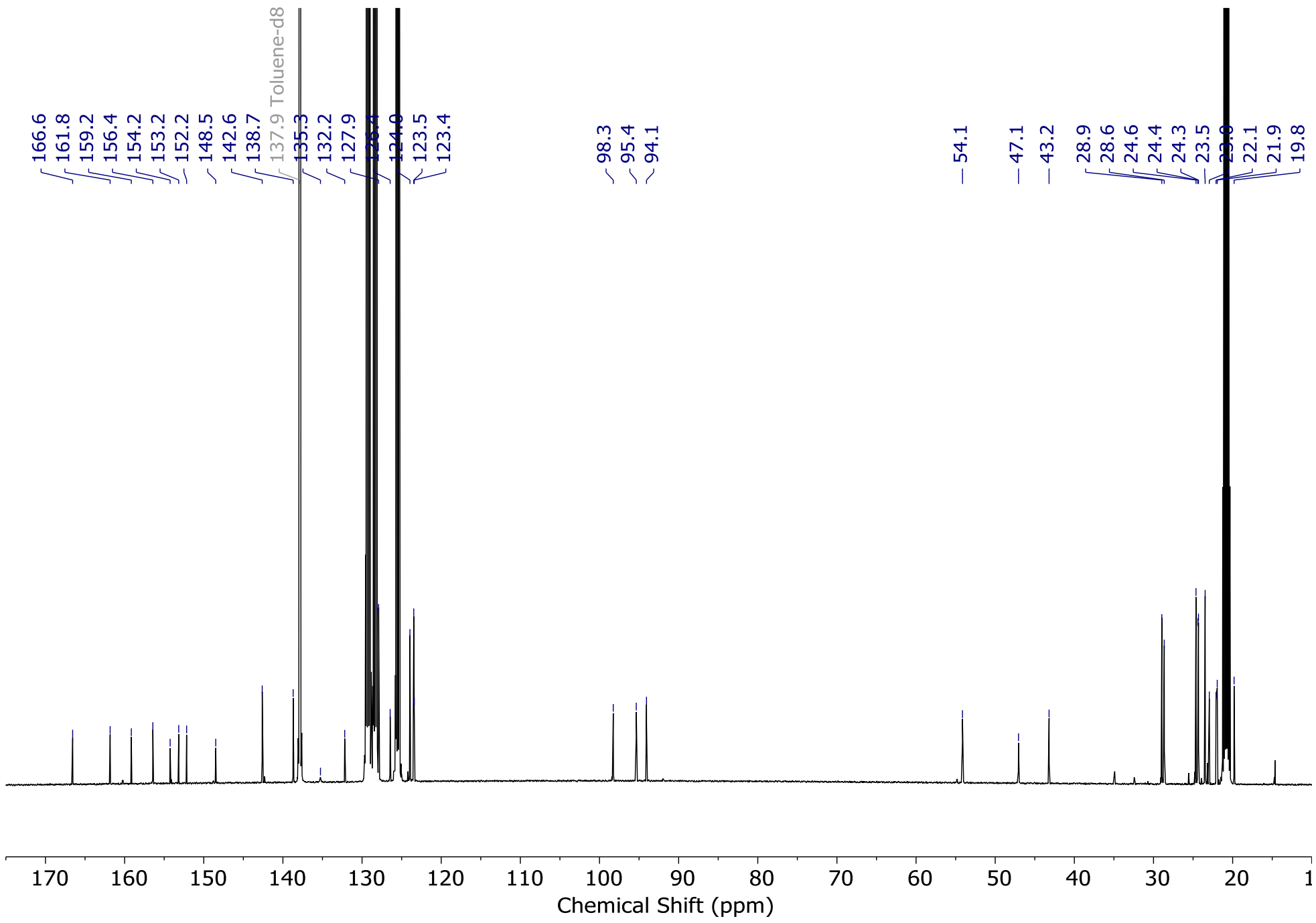

Figure S16: $^{13}C\{^{1}H\}$ NMR spectrum of $\mathbf{2}^{\mathbf{CNBn}}$ in toluene-d_8.

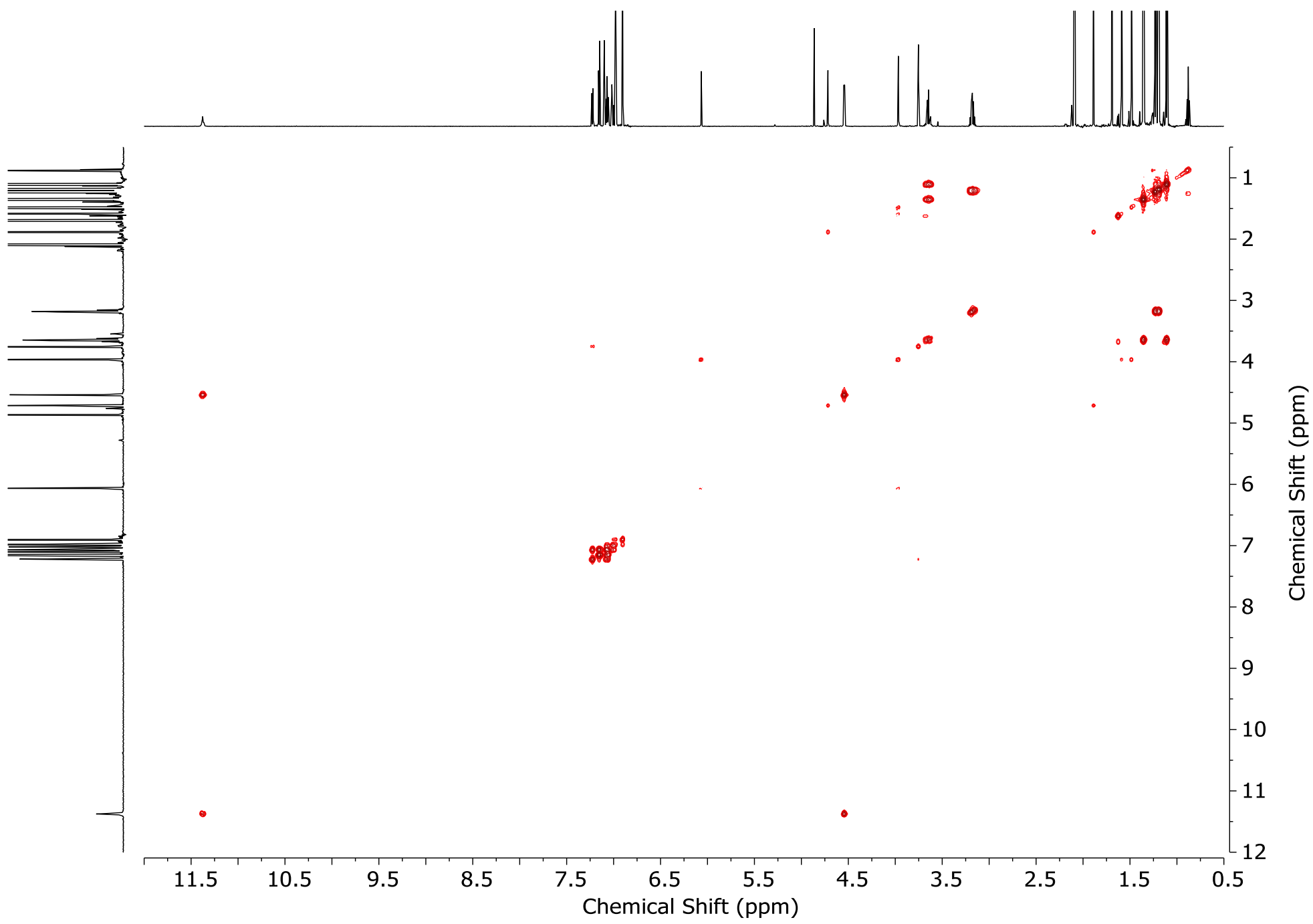

Figure S17: ^{1}H–^{1}H COSY NMR spectrum of $\mathbf{2}^{\mathbf{CNBn}}$ in toluene-d_8.

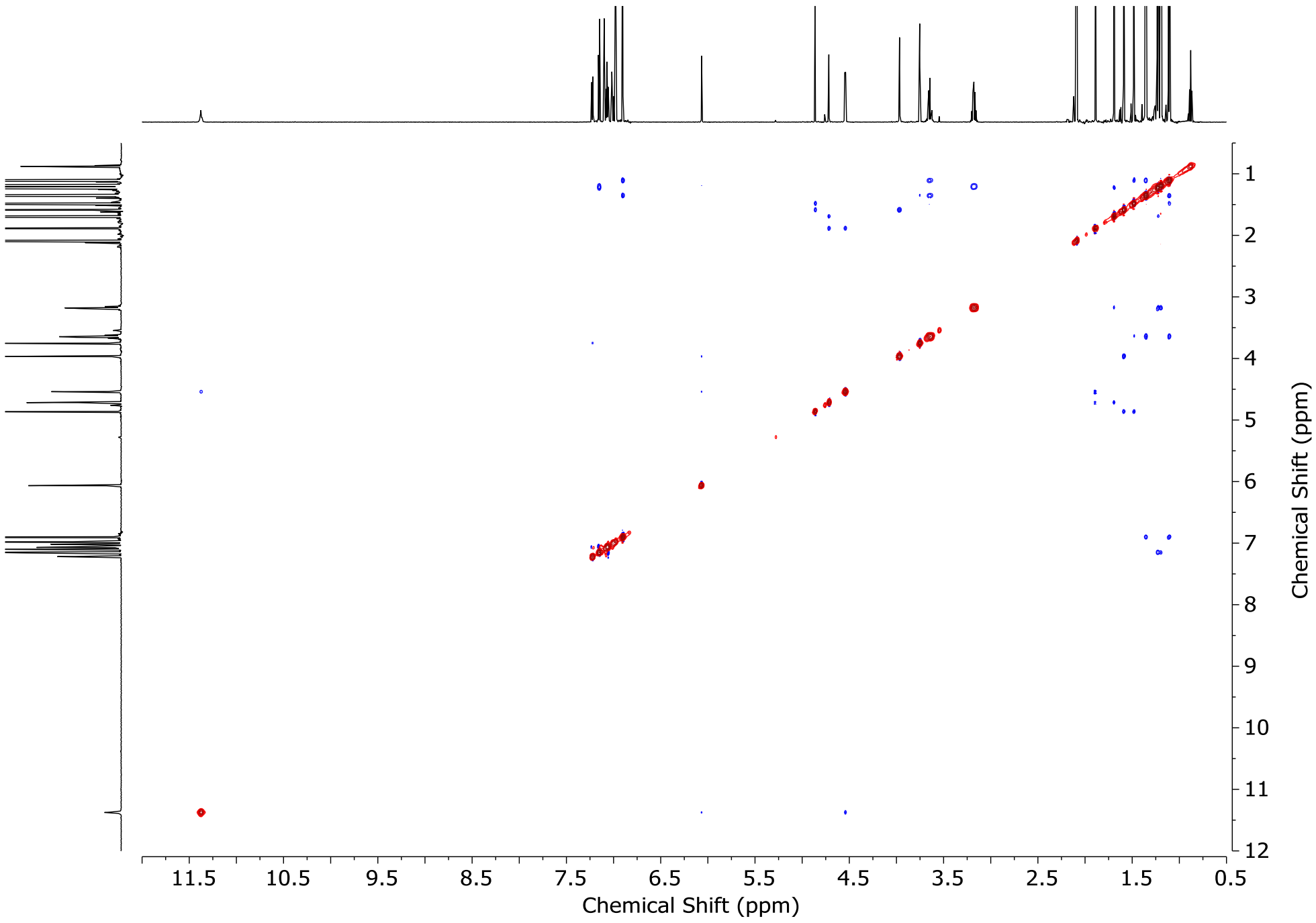

Figure S18: ^{1}H–^{1}H NOESY NMR spectrum of **2**$^{\mathbf{CNBn}}$ in toluene-d$_8$.

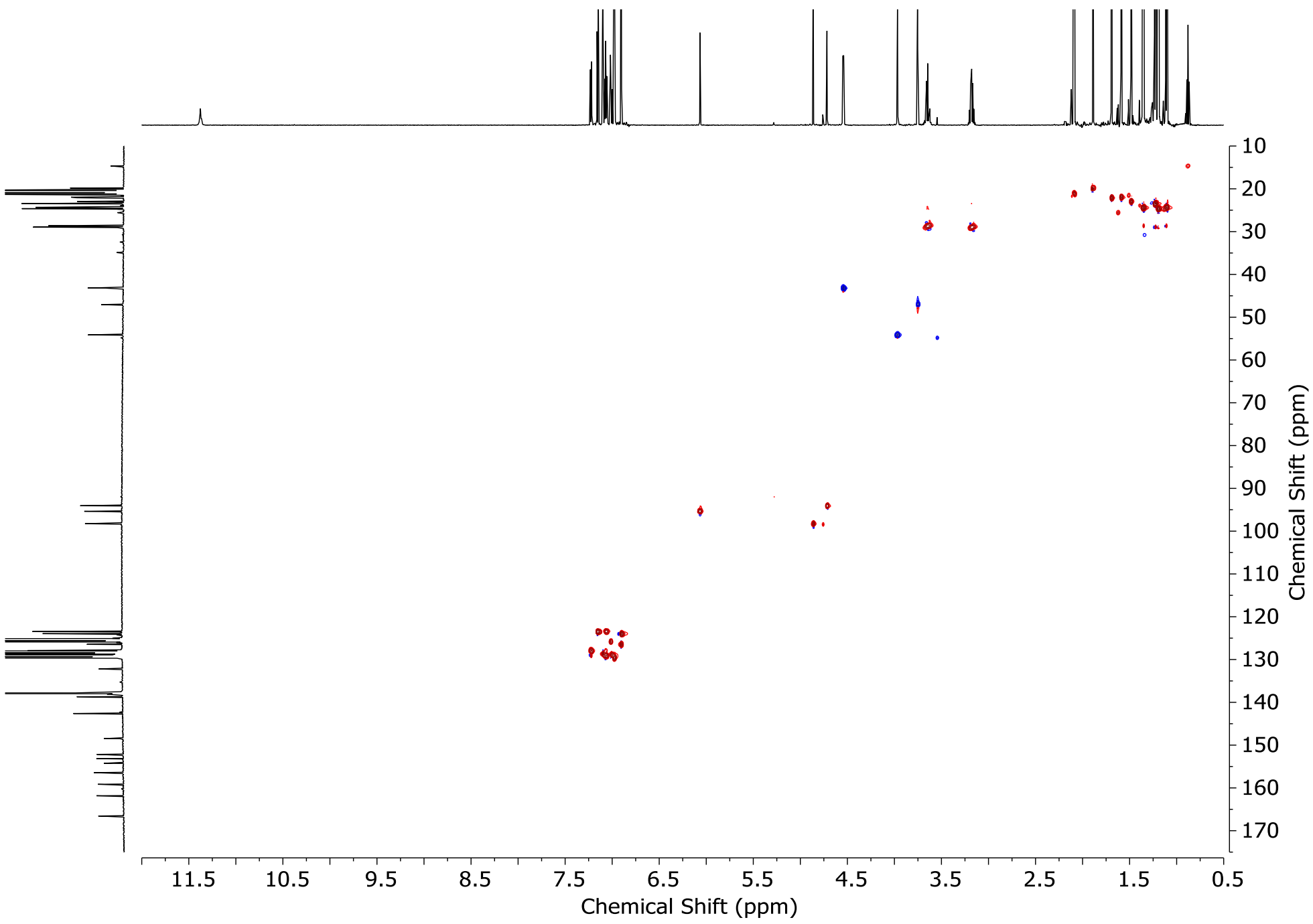

Figure S19: ^{1}H–^{13}C HSQC NMR spectrum of **2**$^{\mathbf{CNBn}}$ in toluene-d$_8$.

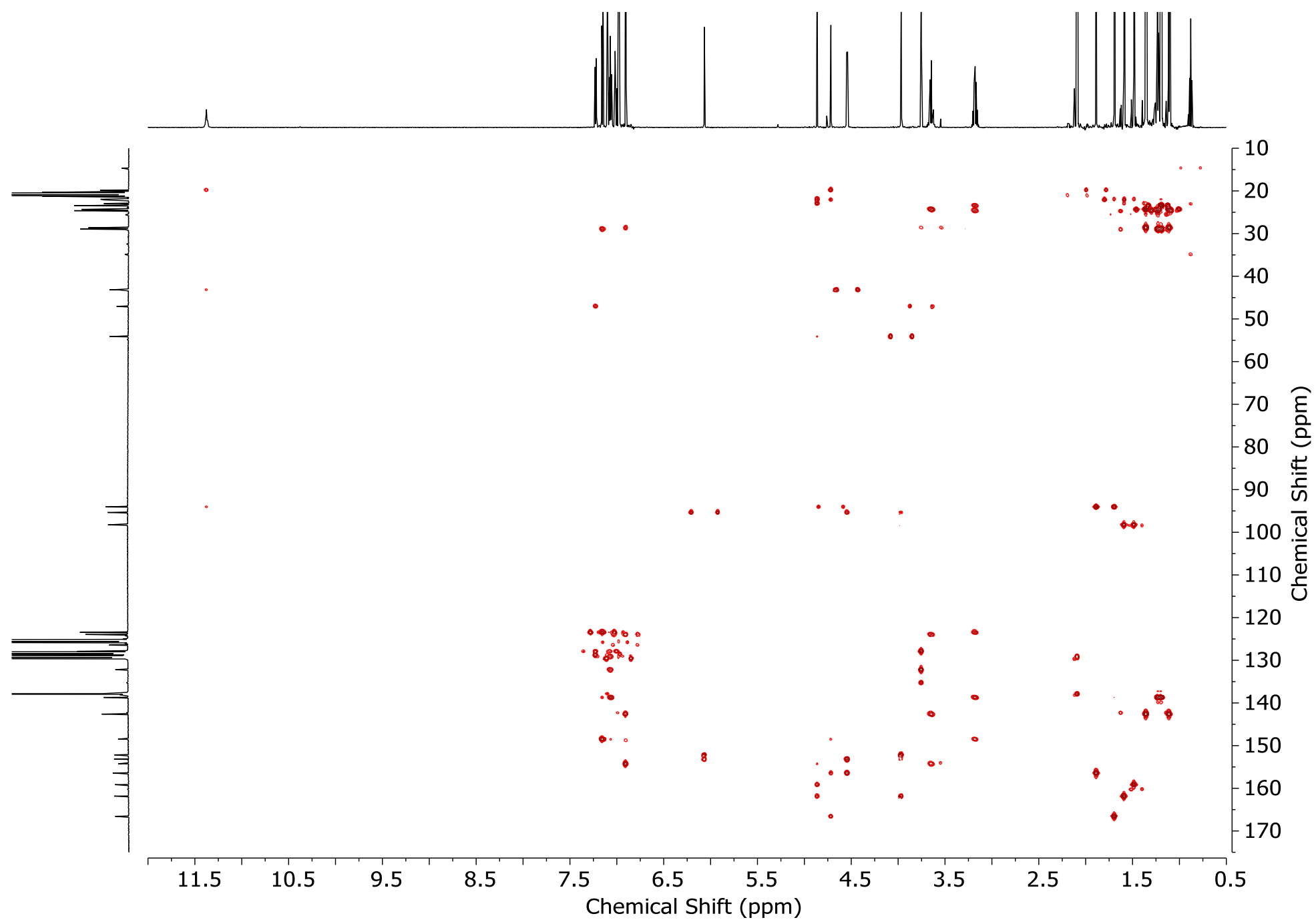

Figure S20: ^{1}H–^{13}C HMBC NMR spectrum of **2**$^{\mathbf{CNBn}}$ in toluene-d_8.

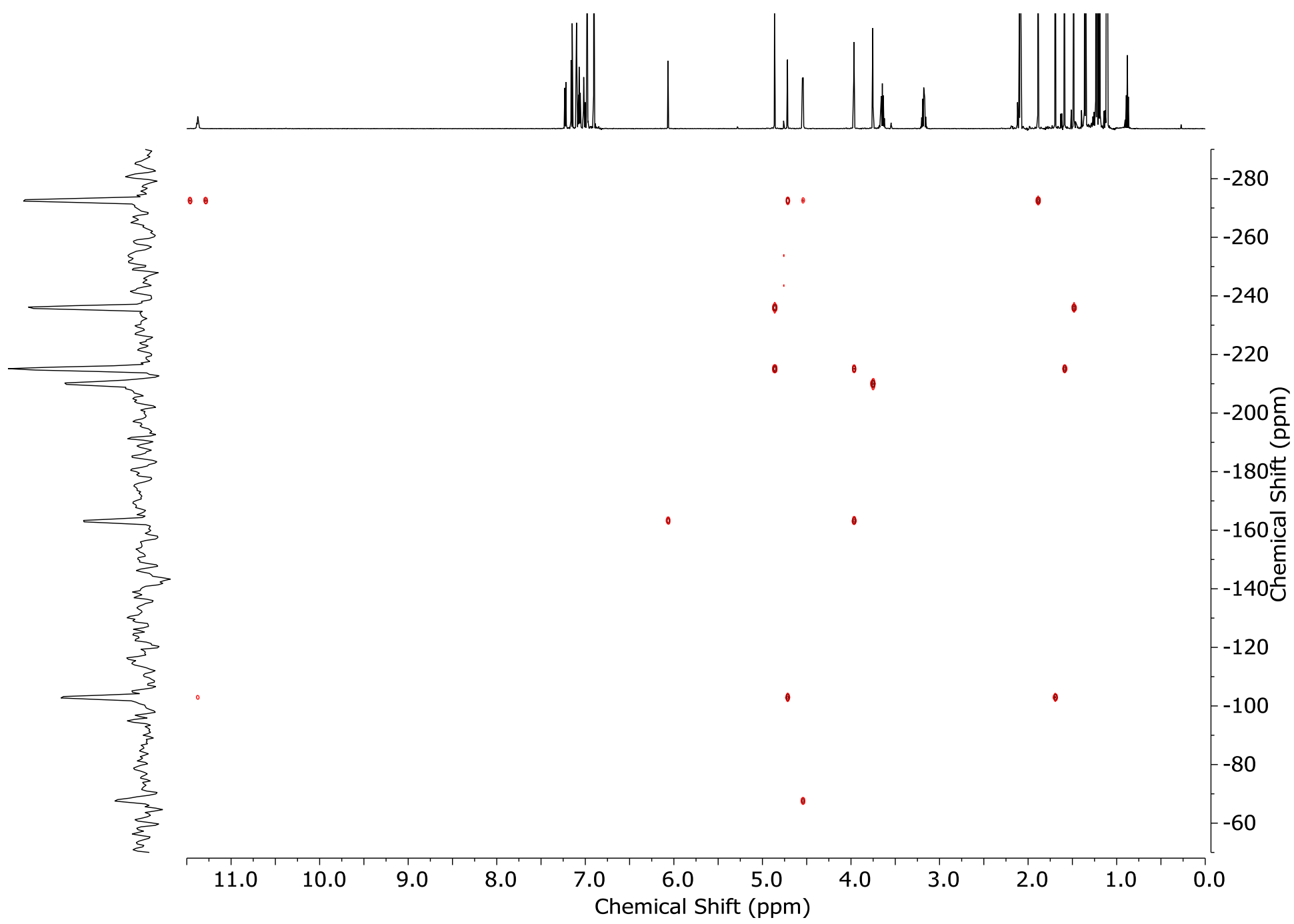

Figure S21: ^{1}H–^{15}N HMBC NMR spectrum of **2**$^{\mathbf{CNBn}}$ in toluene-d_8.

ESI Mass Spectrometry Data

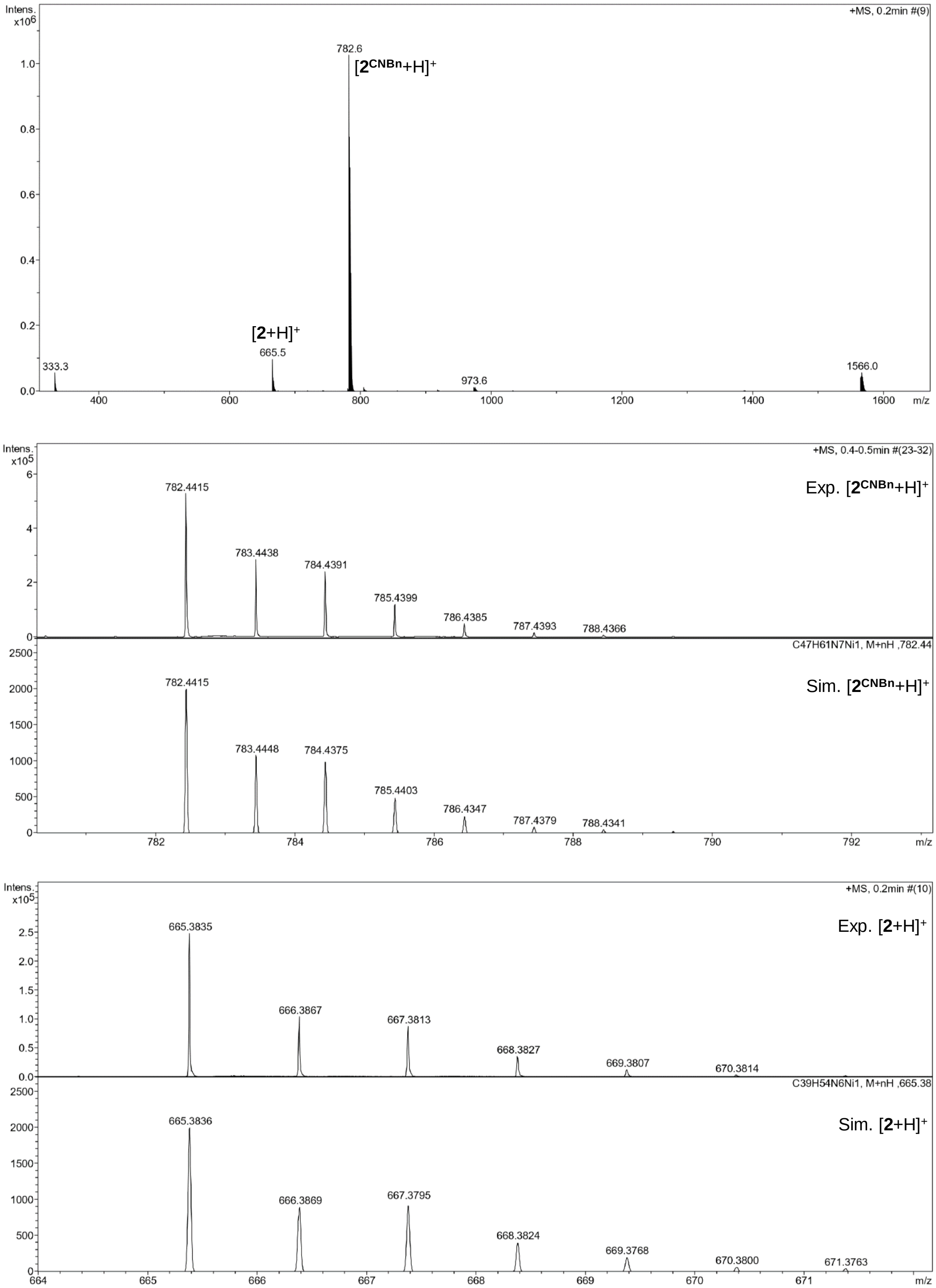

Figure S22: Top: ESI mass spectrum of $\mathbf{2}^{\mathbf{CNBn}}$ in toluene. Middle: experimental and simulated isotopic pattern corresponding to $[\mathbf{2}^{\mathbf{CNBn}}+H]^+$ at *m*/*z* = 782.4. Bottom: experimental and simulated isotopic pattern corresponding to $[HLNi+H]^+$ at m/z = 665.4

IR and UV-Vis Spectroscopic Data

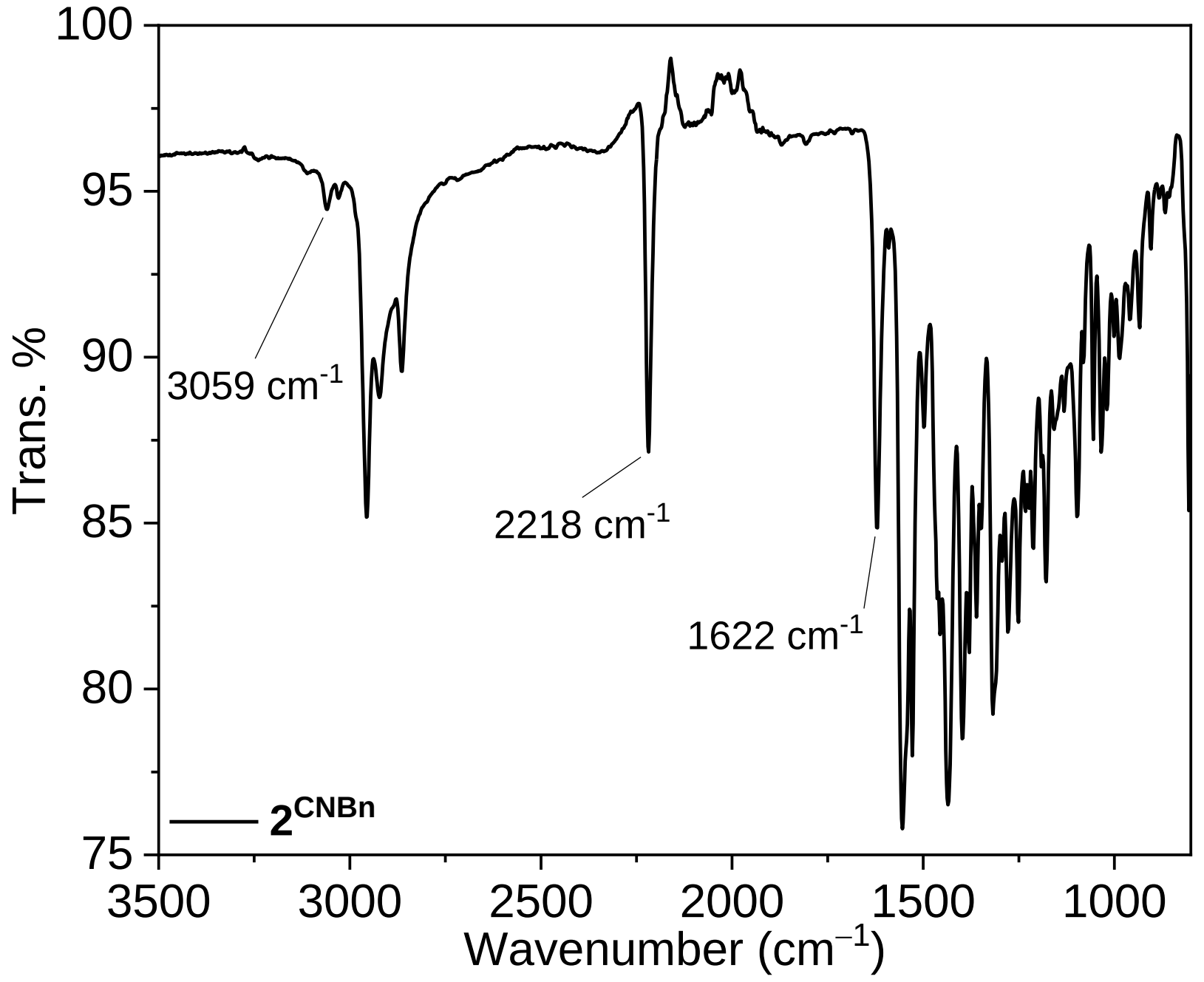

Figure S23: ATR-IR spectrum of crystalline $\mathbf{2}^{\mathbf{CNBn}}$.

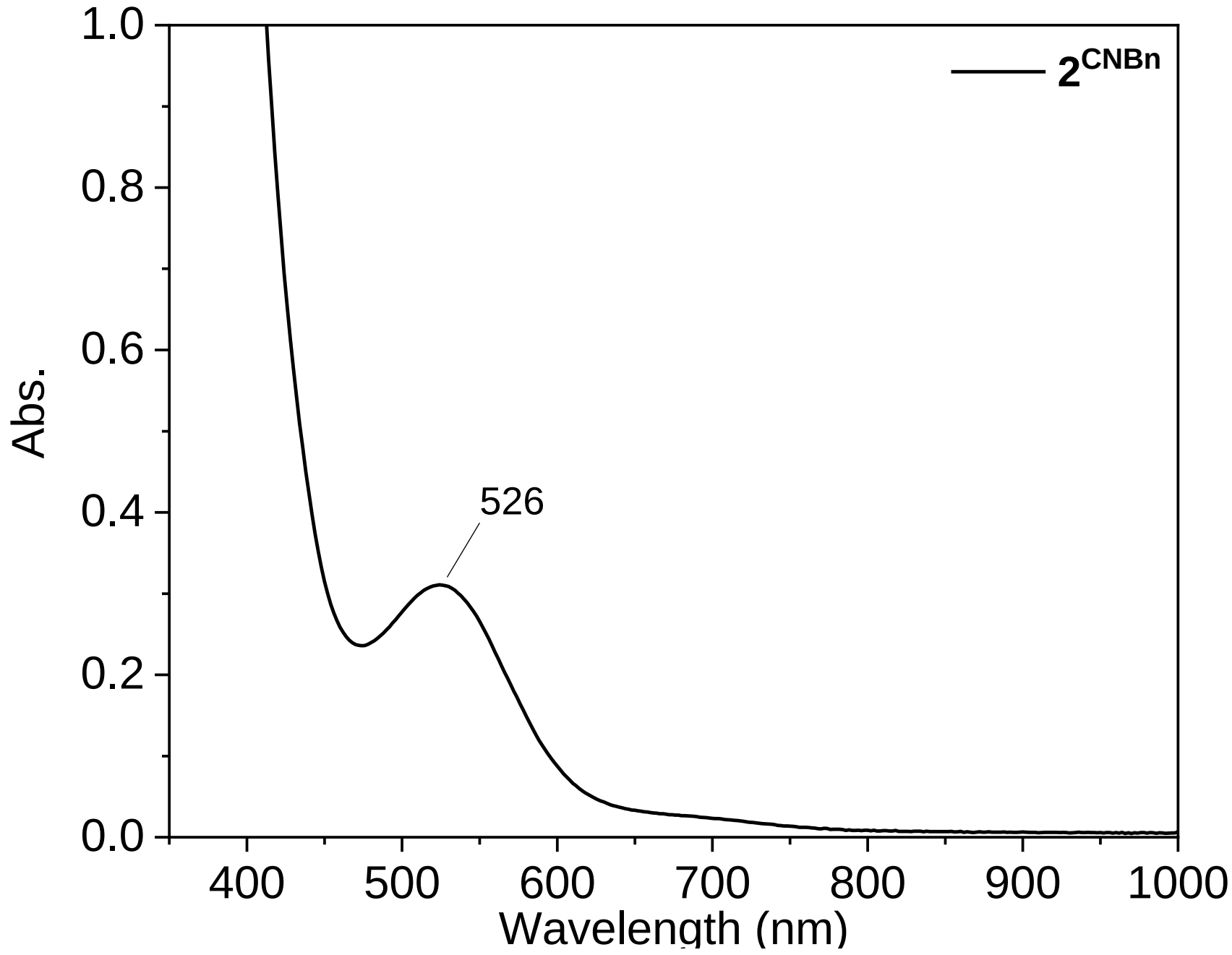

Figure S24: UV-vis spectrum of $\mathbf{2}^{\mathbf{CNBn}}$ in toluene (c = 2.5×10^{-3} M).

X-Ray Crystallography

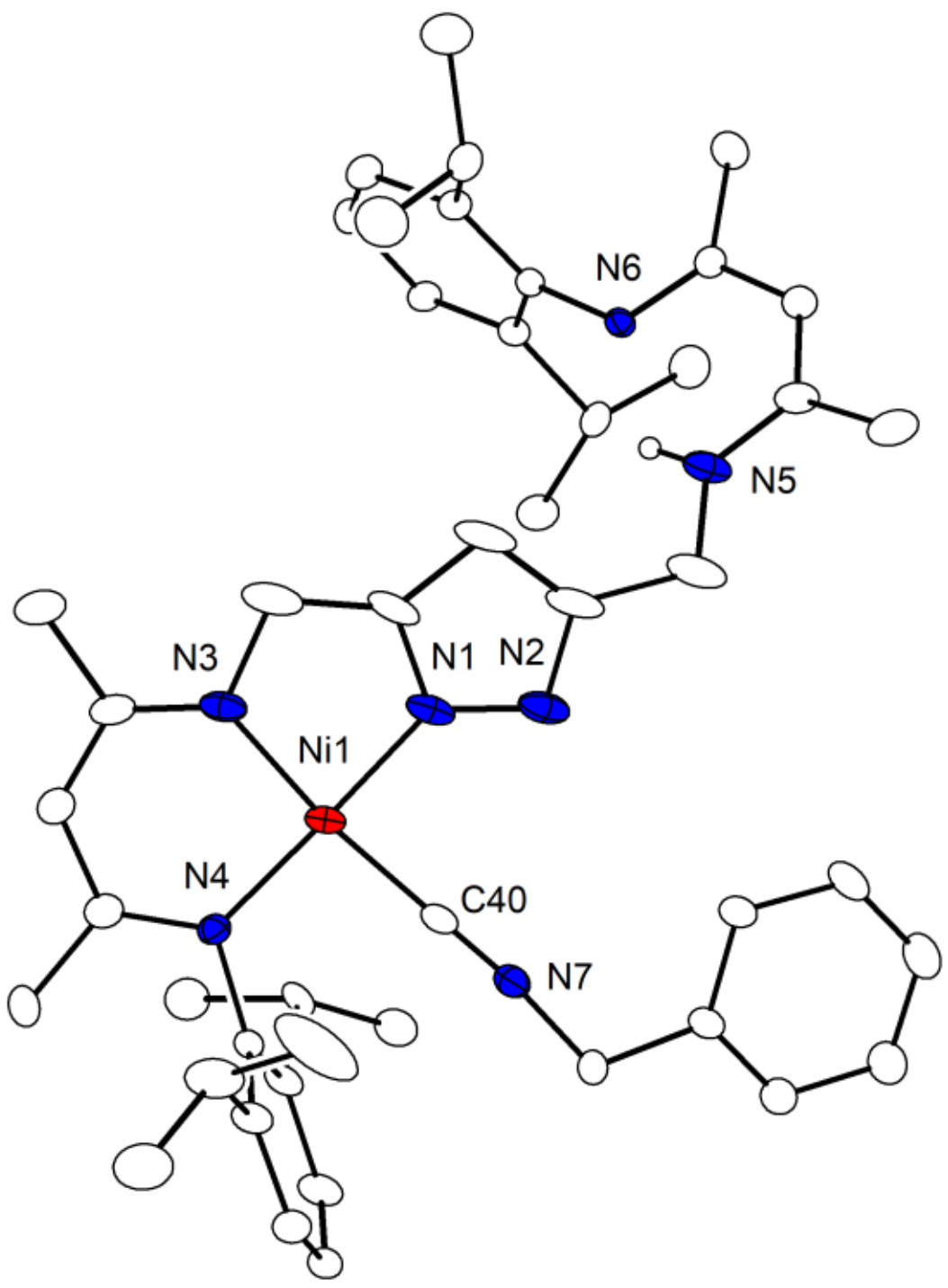

Figure S25: Plot (30% probability thermal ellipsoids) of the molecular structure of **2**^{CNBn} (hydrogen atoms except the H−N5and disorder omitted for clarity). Selected bond lengths [Å] and angles [°]: Ni1–C40 1.831(7), Ni1–N1 1.845(5), Ni1–N3 1.870(6), Ni1–N4 1.896(5), C40–N7 1.165(9), N5···N6 2.682(7); C40–Ni1–N1 88.9(3), C40–Ni1–N3 172.4(3), N1–Ni1–N3 83.8(3), C40–Ni1–N4 92.5(2), N1–Ni1–N4 177.6(2), N3–Ni1–N4 94.9(2), N7–C40–Ni1 179.4(7), N5–H5···N6 134.3.

2.4 Complex $(HL)_2Ni_3(OH)_2(TBD)$ (3^{TBD})

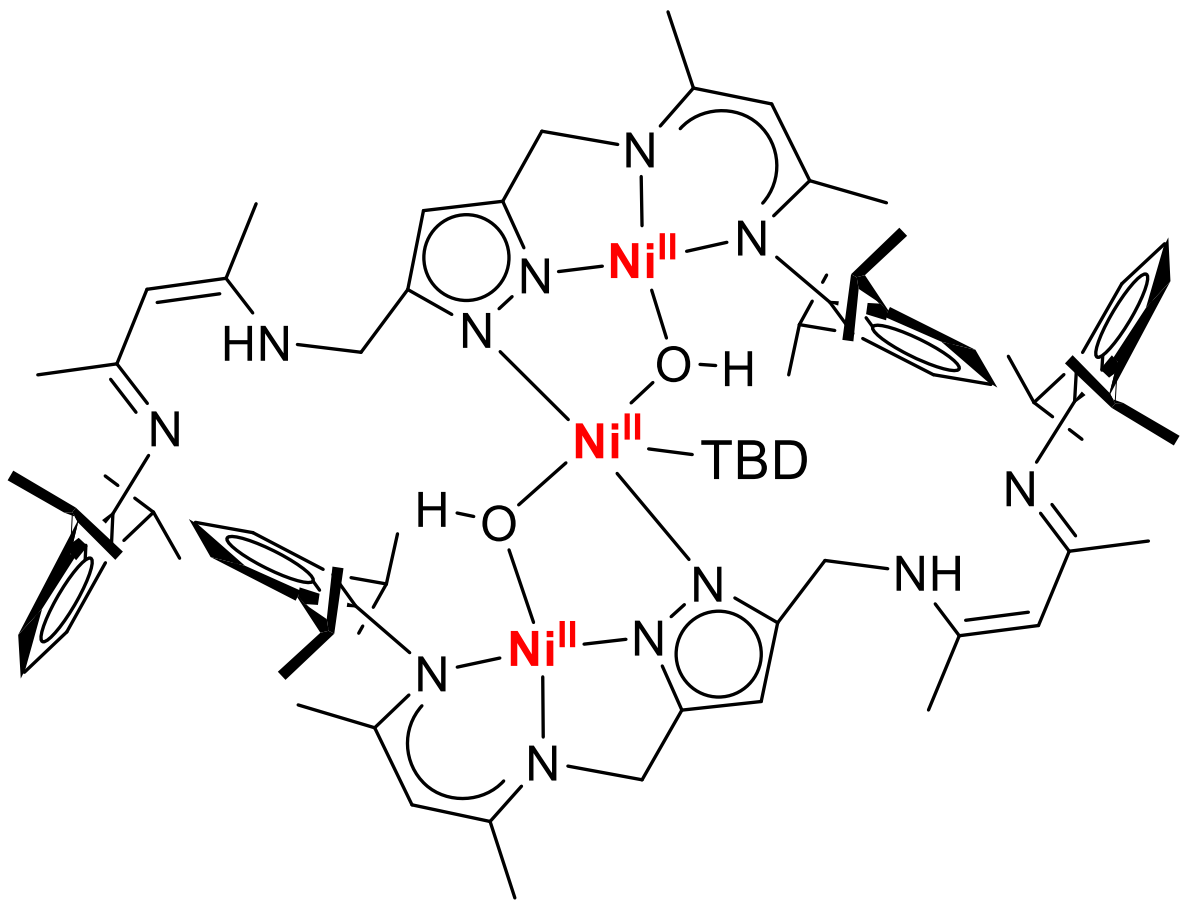

3^TBD

ESI Mass Spectrometry Data

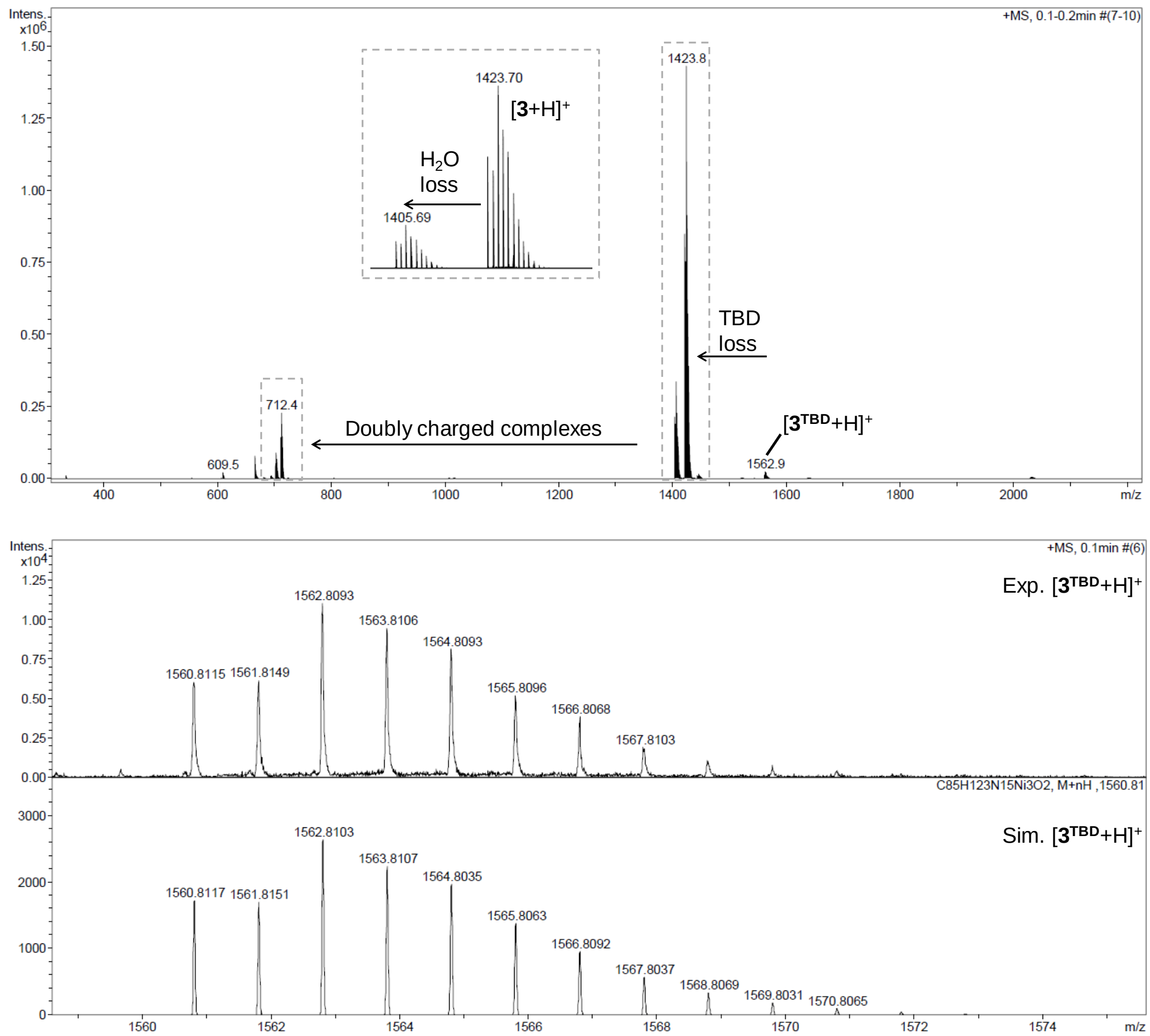

Figure S26. Top: ESI-MS(+) spectrum of 3^{TBD} in THF. Bottom: experimental and simulated isotopic pattern of the peak corresponding to $[3^{TBD}+H]^+$ at m/z = 1560.8.

X-Ray Crystallography

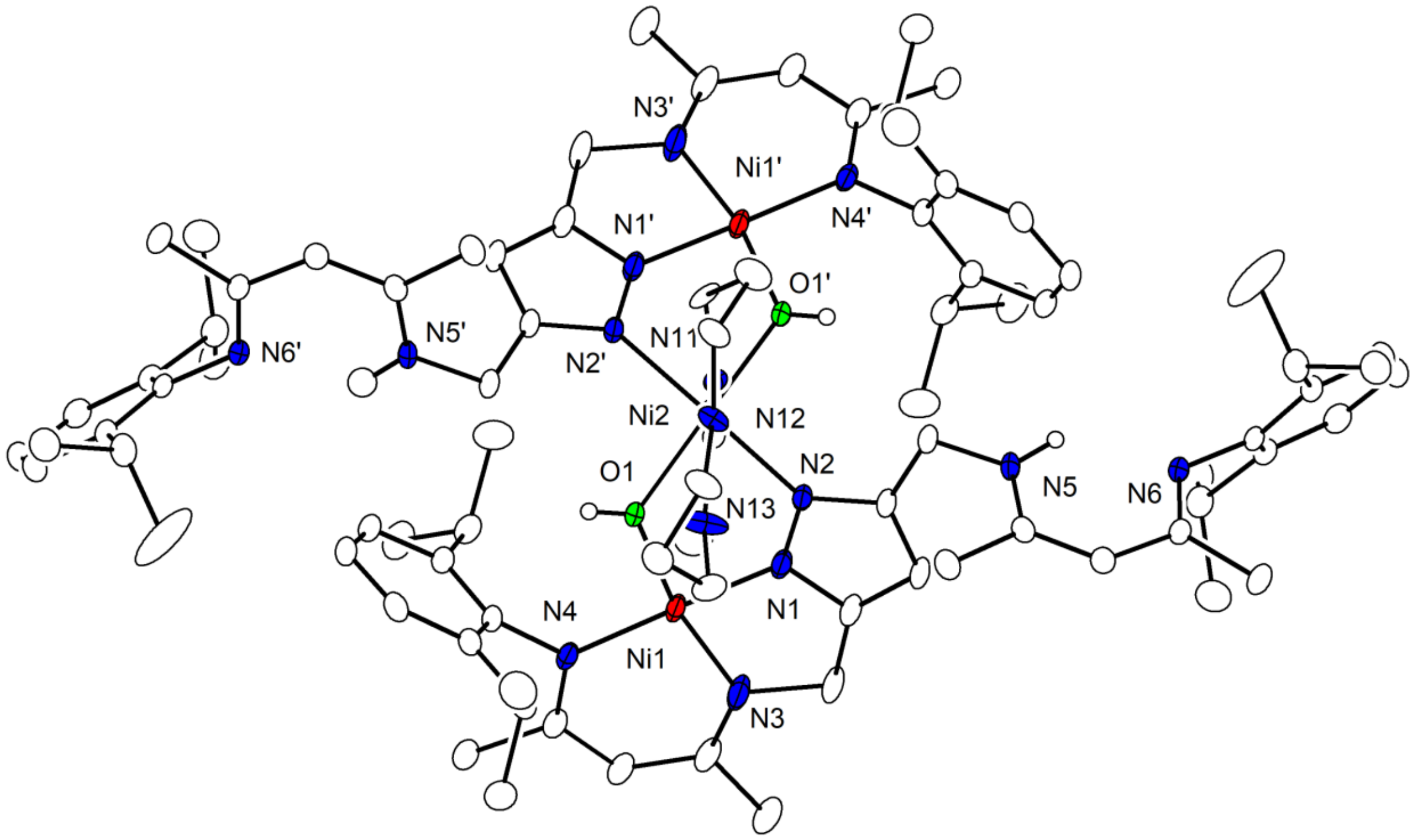

Figure S27. Plot (30% probability thermal ellipsoids) of the molecular structure of the anionic part of **3**$^{\mathbf{TBD}}$ (most hydrogen atoms omitted for clarity). Selected bond lengths [Å] and angles [°]: Ni1–N1 1.849(2), Ni1–O1 1.8649(19), Ni1–N3 1.880(2), Ni1–N4 1.902(2), Ni2–N2 2.0152(18), Ni2–N2' 2.0153(18), Ni2–N11 2.032(4), Ni2–O1 2.0331(18), Ni2–O1' 2.0332(18); N1–Ni1–O1 85.35(8), N1–Ni1–N3 82.76(10), O1–Ni1–N3 168.10(9), N1–Ni1–N4 178.66(9), O1–Ni1–N4 95.87(8), N3–Ni1–N4 96.03(10), N2–Ni2–N2' 164.90(12), N2–Ni2–N11 104.03(12), N2'–Ni2–N11 90.76(12), N2–Ni2–O1 82.59(7), N2'–Ni2–O1 93.64(7), N11–Ni2–O1 116.56(13), N2–Ni2–O1' 93.64(7), N2'–Ni2–O1' 82.59(7), N11–Ni2–O1' 92.14(13), O1–Ni2–O1' 151.16(14), Ni1–O1–Ni2 122.68(9). Symmetry transformation used to generate equivalent atoms: (') 1–*x*, *y*, 3/2–*z*.

3. Additional Data

3.1 Detection of the CO_2 Produced During the Reaction of 1^{OH} with CO

NMR scale reaction (ca. 6 mg of **1**) for GC analysis: after the completion of the reaction of **1** with dry CO, a rubber septum was attached to the J. Young NMR-tube and the headspace was purged with argon. The tube was opened and a sample (1 mL) of the headspace was taken and injected into the GC (Figure S28).

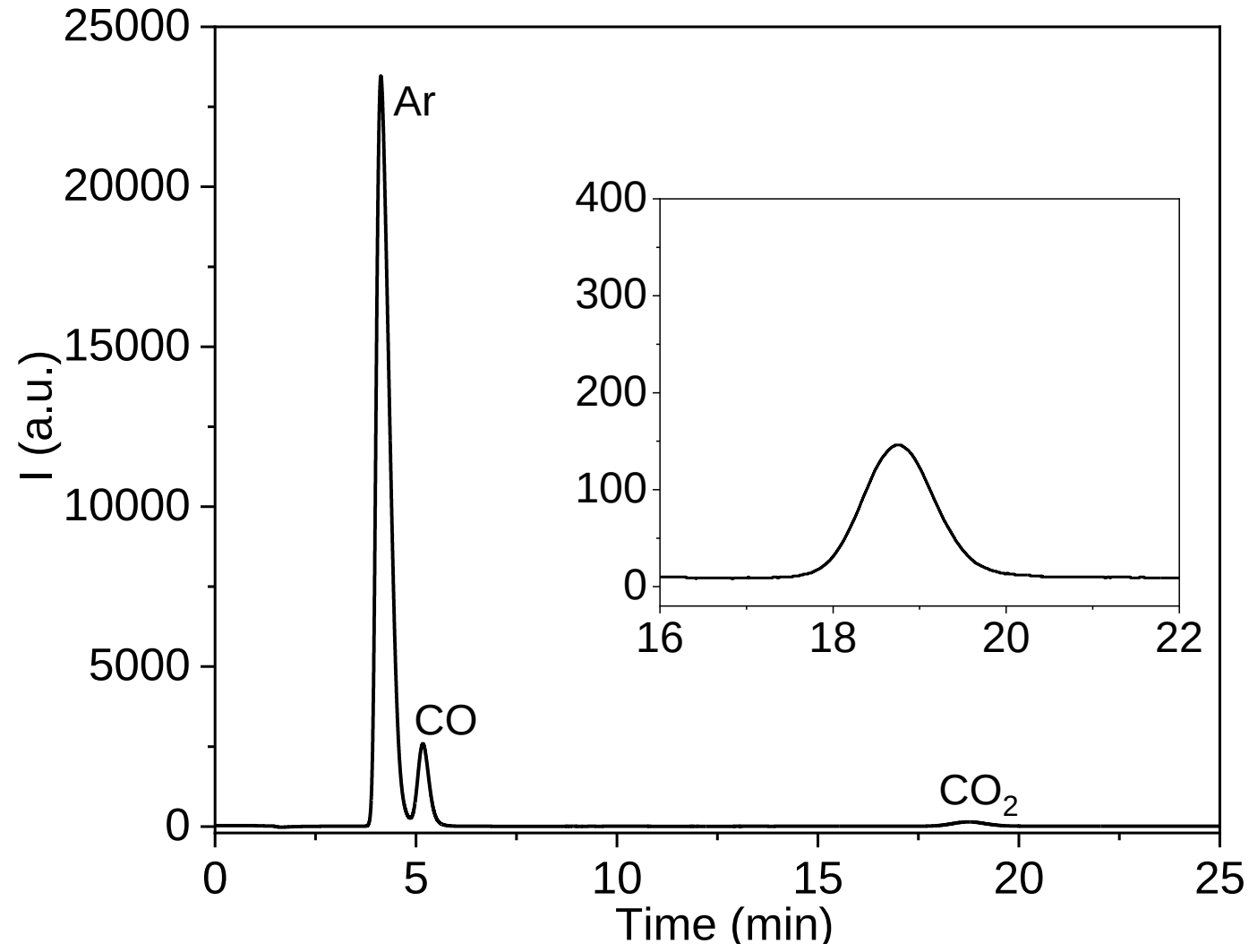

Figure S28: GC trace of the headspace of the reaction of **1** with CO: peaks corresponding to Ar, CO and CO_2 at retention times 4, 5 and 19 min, respectively.

30 mg **1** scale reaction for IR spectroscopy analysis of the headspace of the reaction mixture (Figure S29): the reaction was performed as above described using dry ^{12}CO or ^{13}CO. After the completion of the reaction the flask was connected *via* a three-way valve to a Schlenk line and to the IR cell. The IR cell was evacuated, the reaction mixture solution was cooled to –60 °C, the and the headspace vacuum transferred to the IR cell ready for analysis. The same procedure adding dry CO to pure toluene was used to record a background spectrum.

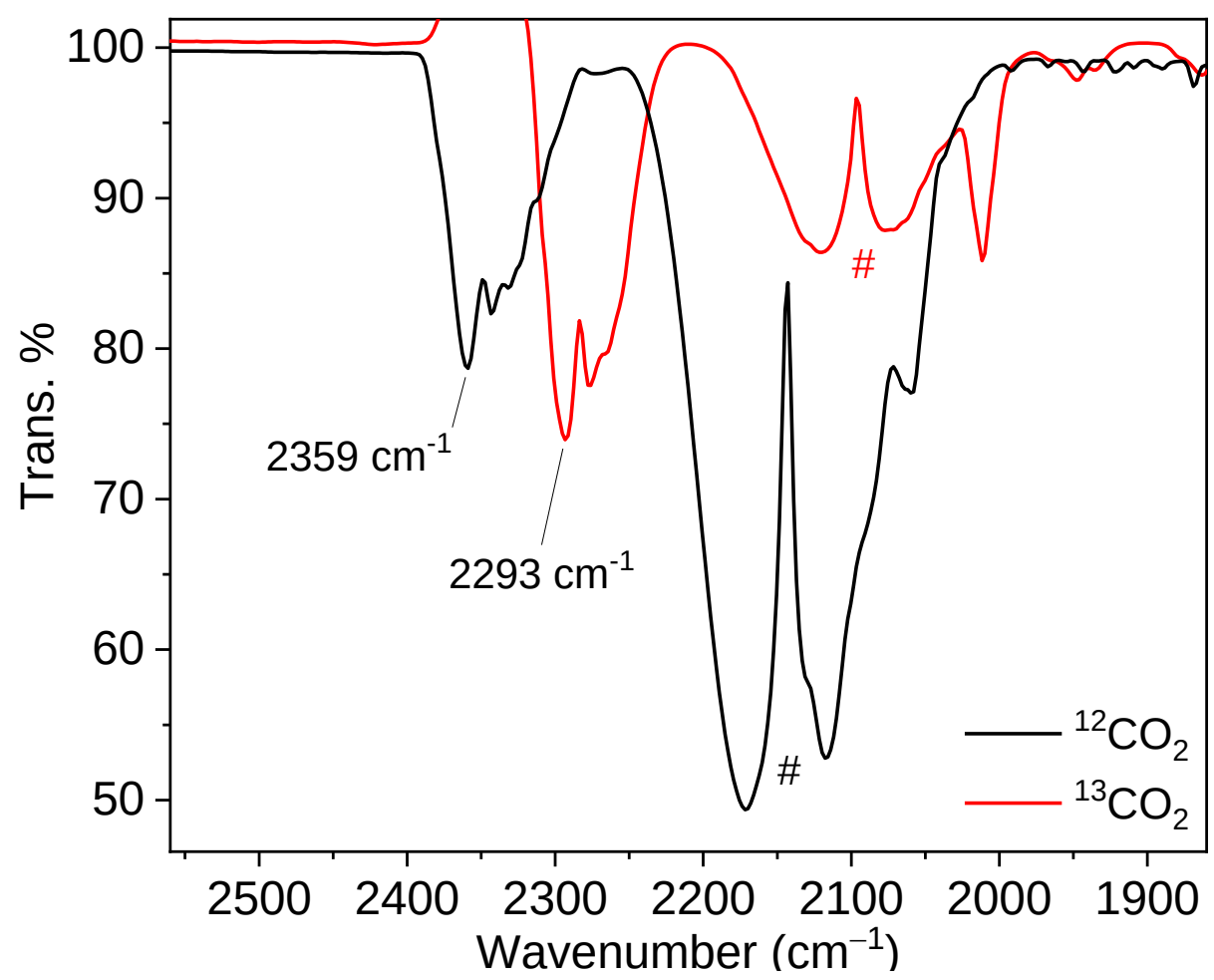

Figure S29: IR spectra of the headspace of the reaction of **1** with ^{12}CO (black line) and with ^{13}CO (red line) showing the presence of $^{12}CO_2$ and $^{13}CO_2$, respectively. # indicates ^{12}CO/^{13}CO.

3.2 Detection of the $Ni(CO)_4$ Produced During the Reaction of 1^{OH} with CO

NMR scale reaction (ca. 6 mg of **1**) for IR spectroscopy analysis (Figure S30): after the completion of the reaction of **1** with dry ^{12}CO or ^{13}CO, the J. Young NMR-tube was evacuated and transferred in a glovebox. The solution was transferred in a Schlenk apparatus and the NMR tube rinsed with 0.5 mL of dry toluene-d_8. The apparatus was transferred outside the glovebox and connected to a Schlenk line. The solution (containing $Ni(CO)_4$) was separated by the complex *via* vacuum distillation. The apparatus was transferred in a glovebox where the distilled fraction was loaded in a OTTLE cell. The background was recorded using toluene-d_8.

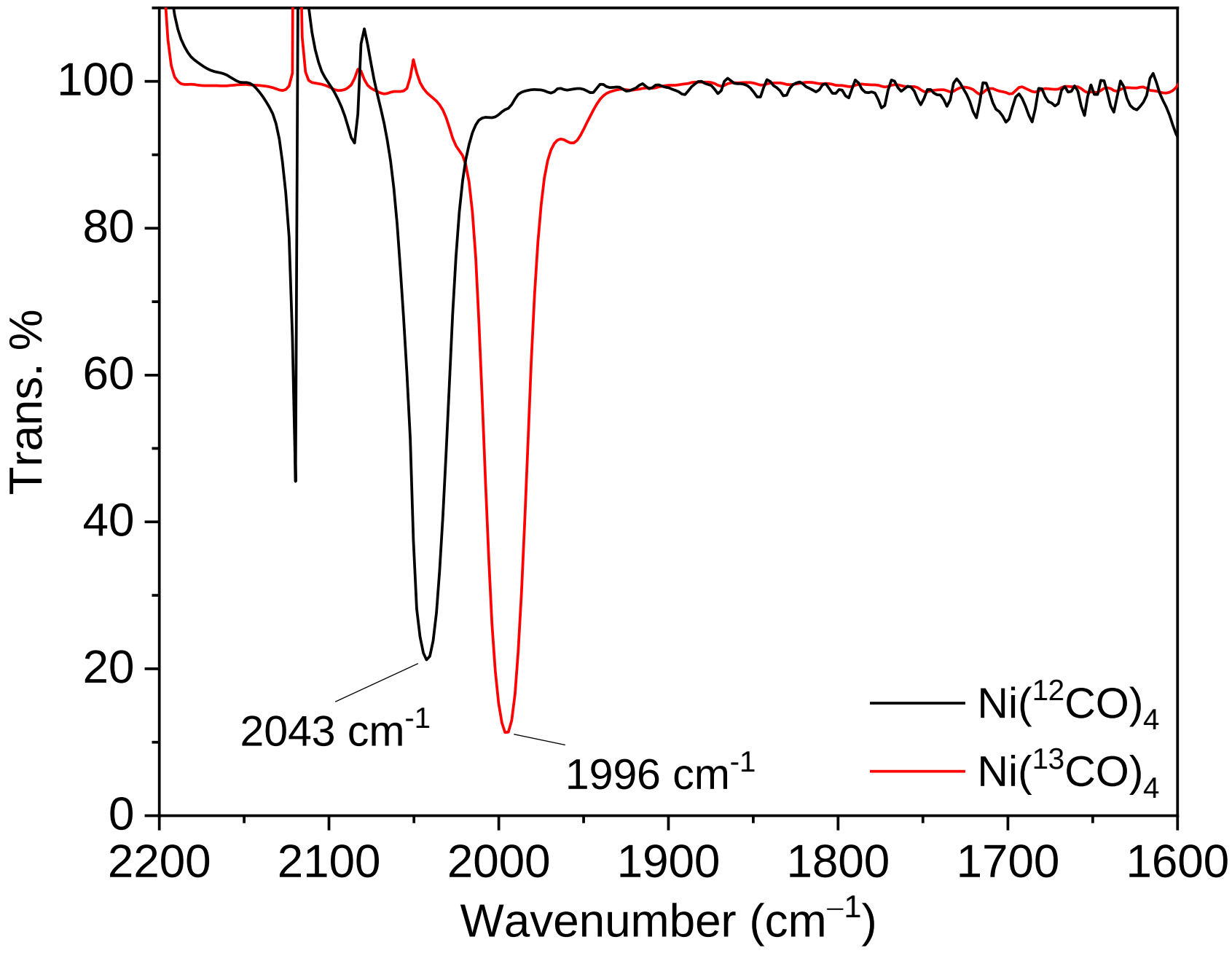

Figure S30: IR spectrum of the distilled fraction from the reaction of **1** with ^{12}CO (black line) or ^{13}CO (red line) corresponding to $Ni(^{12}CO)_4$/$Ni(^{13}CO)_4$.

3.3 Reaction of 1 with CO directly from the cylinder and decomposition to $^{H}L_2Ni_3(OH)_2$

NMR scale reaction (5 mg of **1**): wet CO was added to a solution of **1** in dry toluene-d_8. After 2h the sample was subjected to ^1H NMR and $^{13}C\{^1H\}$ NMR analysis (Figures S31, S32) and GC analysis, as previously described (Figures S33).

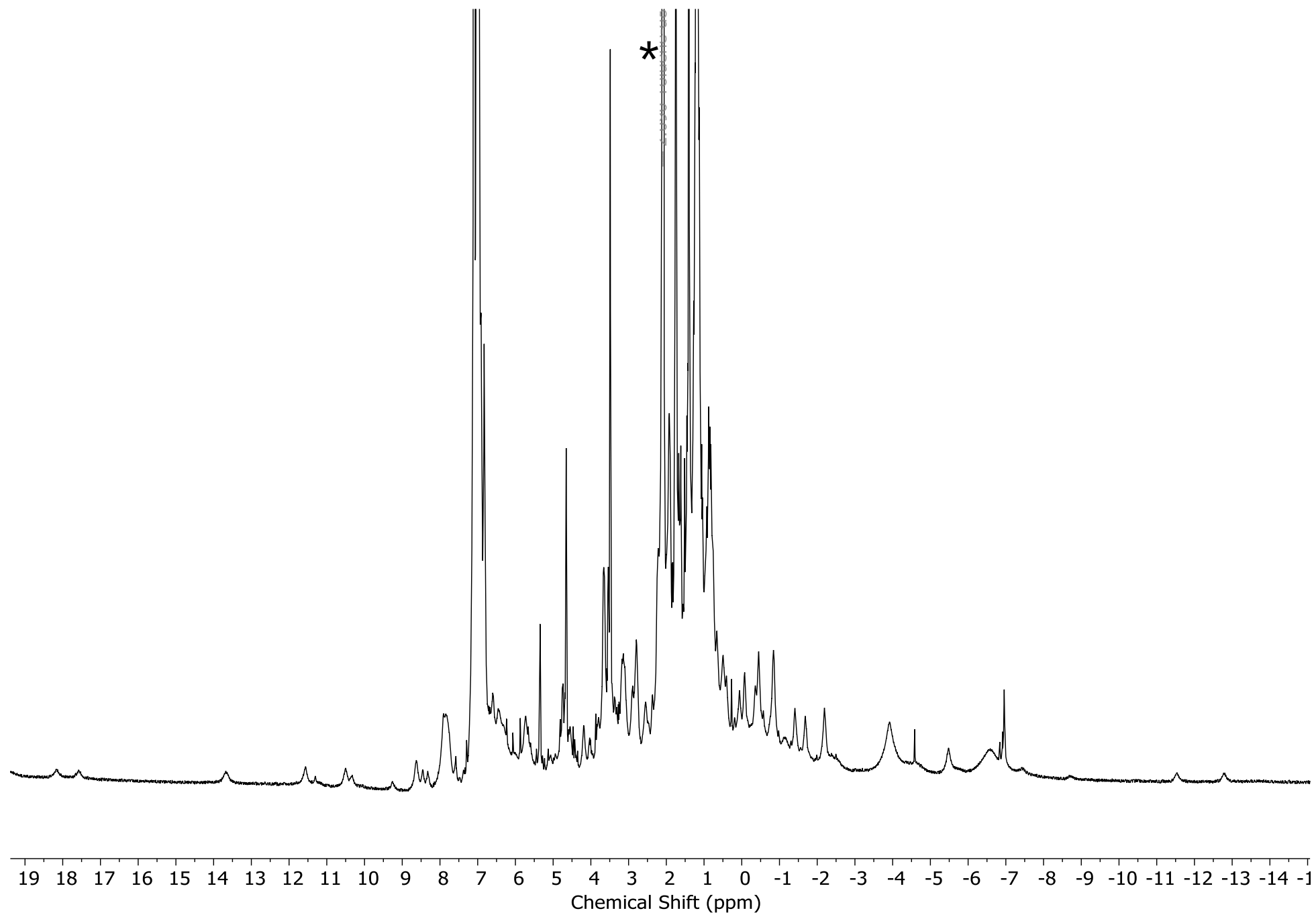

Figure S31: ^1H NMR spectrum recorded after **1** was reacted with wet CO in toluene-d_8. Residual solvent (*) is marked as reported in brackets

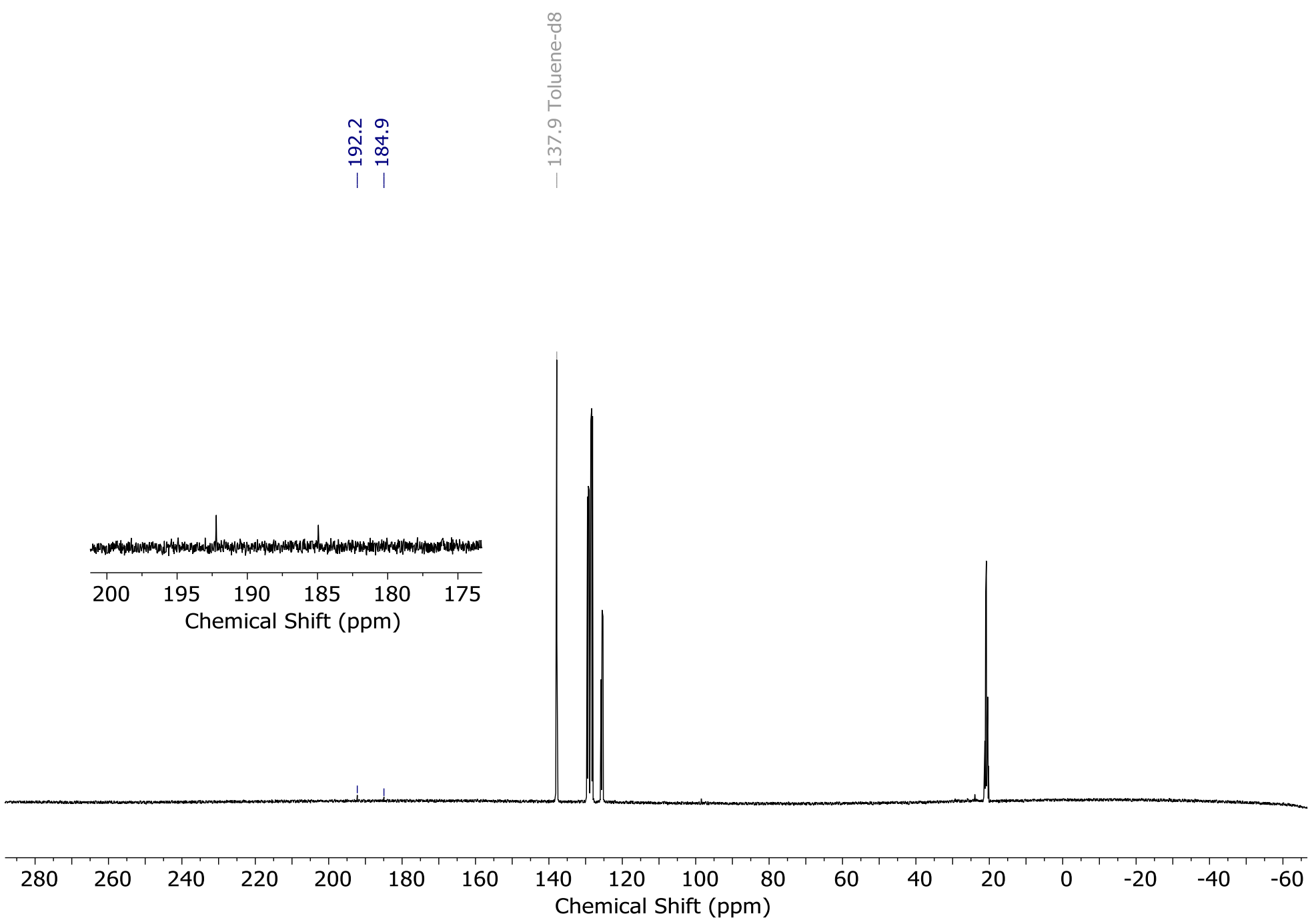

Figure S32: $^{13}C\{^1H\}$ NMR spectrum of the reaction of **1** with wet CO: peaks corresponding to $Ni(CO)_4$ (192.2 ppm) and CO (184.9 ppm) are marked and shown in the enlargement.

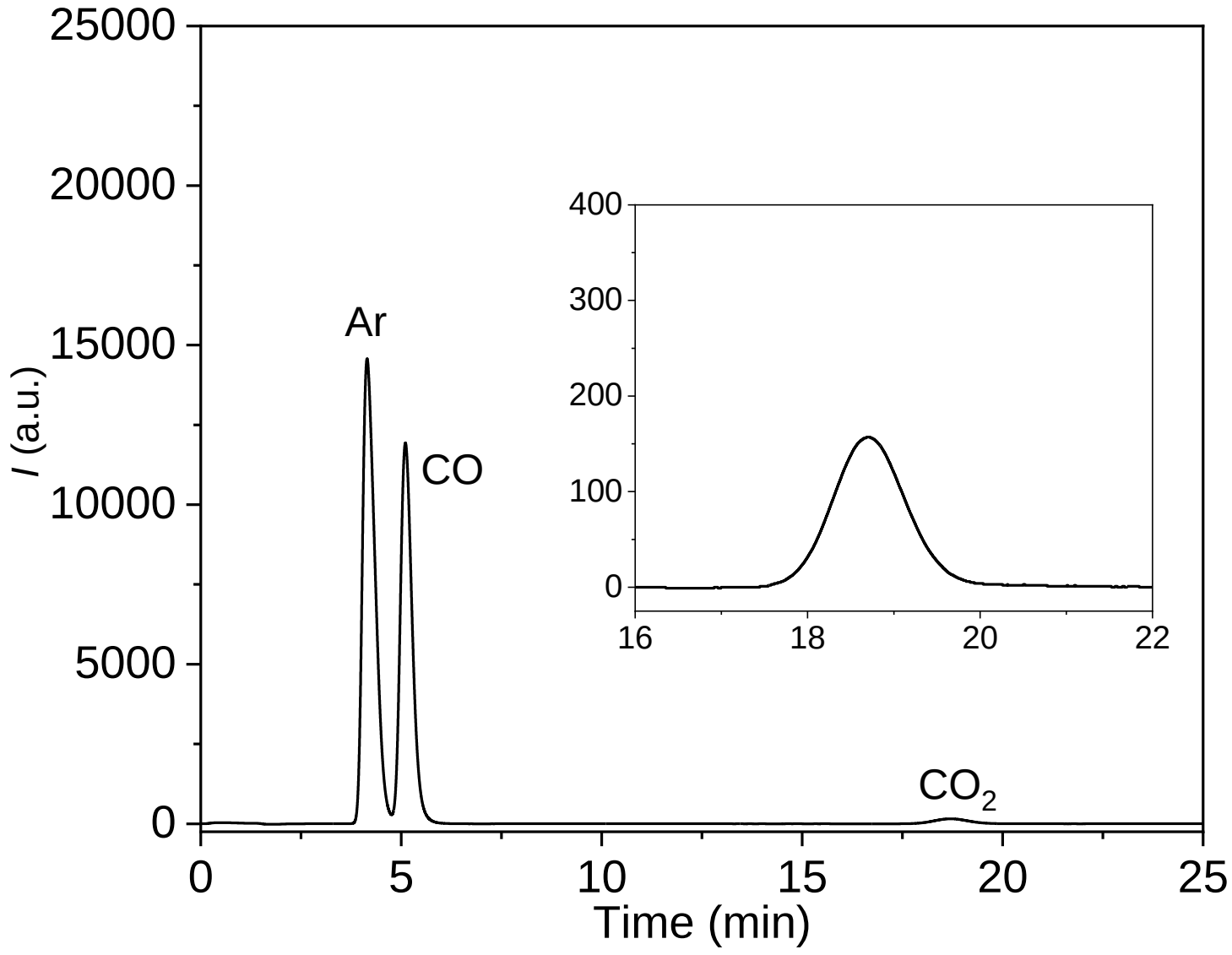

Figure S33: GC trace of the headspace of the reaction of **1** with wet CO: peaks corresponding to Ar, CO and CO_2 at retention times 4, 5 and 19 min, respectively.

A sample of $\mathbf{2}^{\mathbf{CO}}$ was exposed to air and subsequently subjected to IR and ESI-MS analysis (Figures S34, S35).

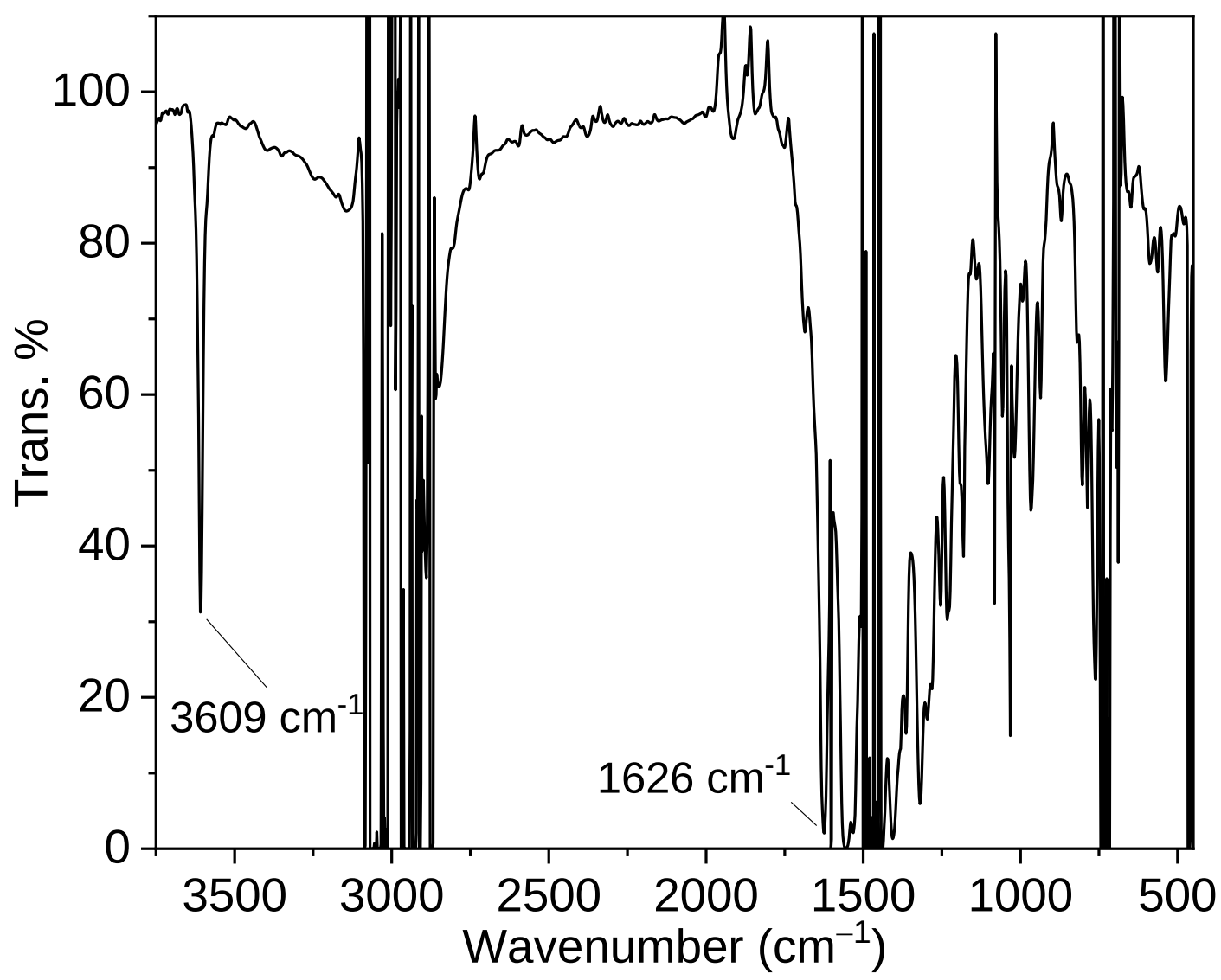

Figure S34: IR spectrum of the toluene solution of $\mathbf{2}^{\mathbf{CO}}$ that was exposed to air.

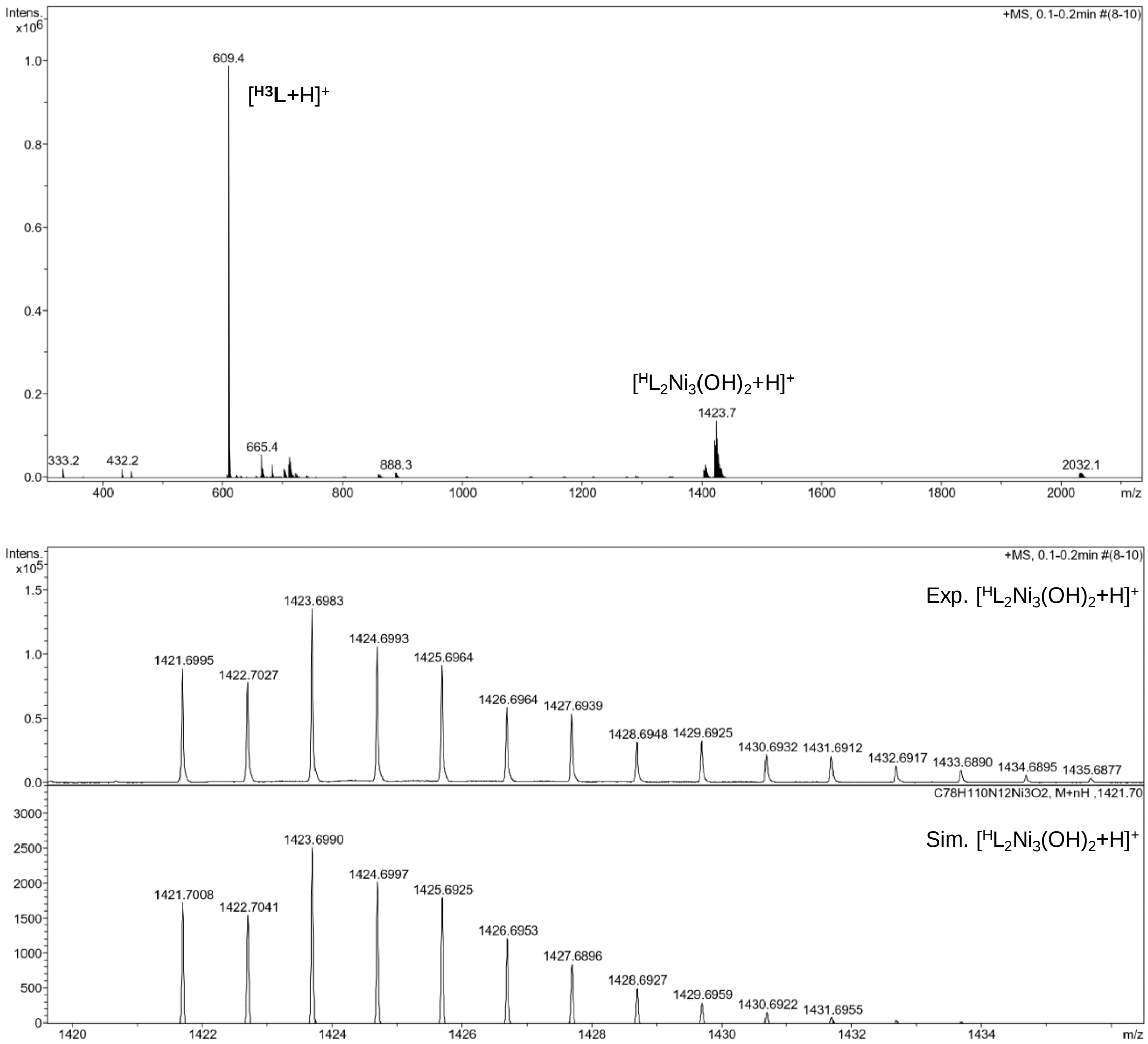

Figure S35: Top: ESI-MS(+) spectrum of the toluene solution obtained after exposure of $\mathbf{2^{CO}}$ to air. Bottom: experimental and simulated isotopic pattern of the peak corresponding to $[(HL)_2Ni_3(OH)_2{+}H]^+$.

3.4 Analysis of the reaction of 1 with CNBn

NMR scale reaction: to a toluene solution of **1** (6 mg, 8.6 mmol, 1.0 eq), CNBn was added (4.6 µL, 8.6 mmol, 1.0 eq). ^{1}H NMR and ESI-MS spectra of the reaction mixture are shown in Figures S36 and S37.

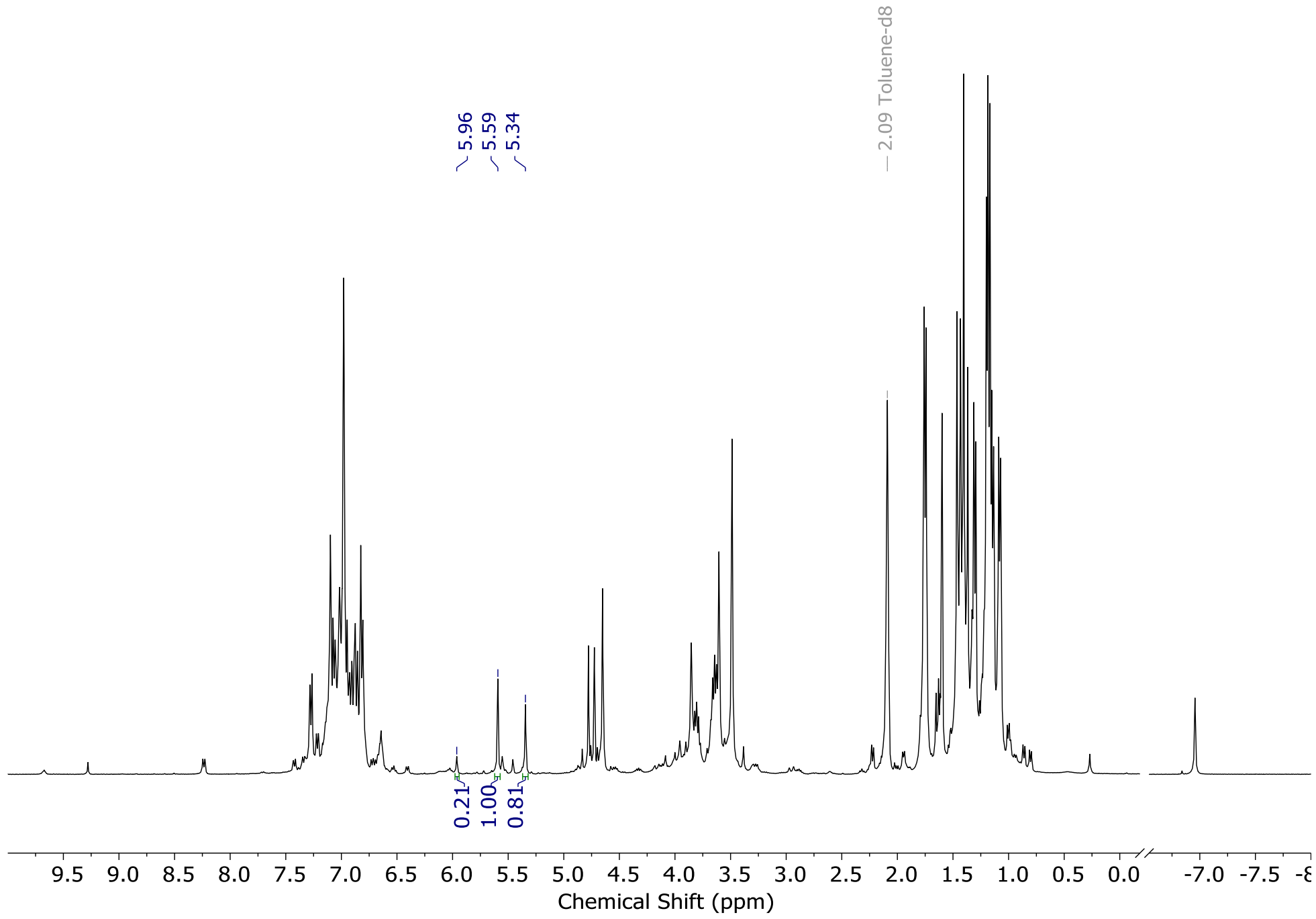

Figure S36: ^{1}H NMR spectrum of the crude reaction mixture of **1** with 1.0 eq of CNBn in toluene-d_8.

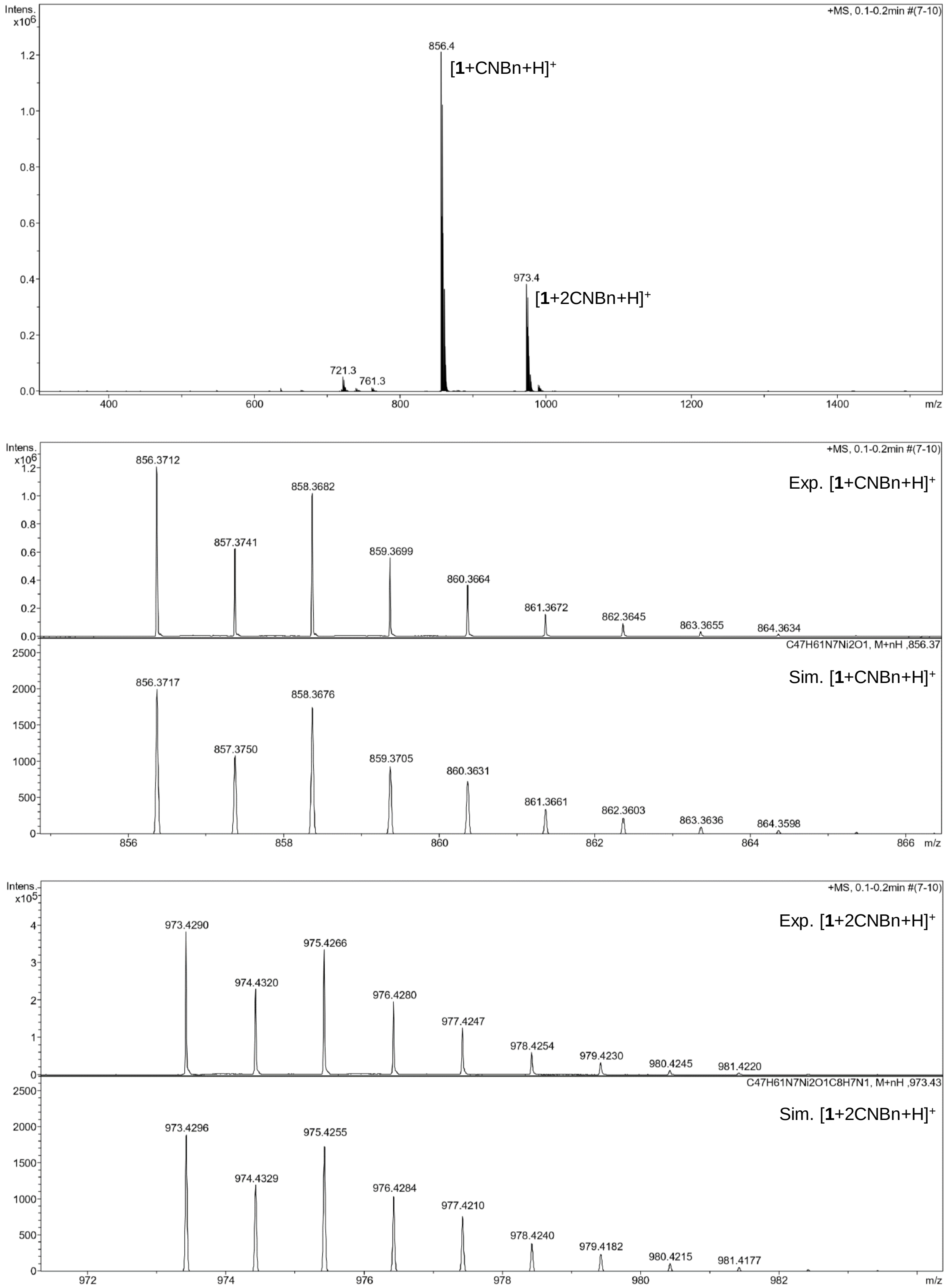

Figure S37: Top: ESI-MS(+)of **1** after reaction with 1 eq. of CNBn in toluene. Middle: section of the spectrum showing the peak and the simulated isotopic pattern corresponding to $[\mathbf{1}\text{+CNBn+H}]^+$ at m/z = 856.4. Bottom: section of the spectrum showing the peak and the simulated isotopic pattern corresponding to $[\mathbf{1}\text{+2CNBn+H}]^+$ at m/z = 973.4.

4. Computational Details

Geometry optimizations and harmonic frequency calculations were performed with the ORCA[1,2] program package employing Grimme's r^{2}SCAN-3c[3] composite scheme which makes use of the meta-generalized-gradient approximation (mGGA) and a specifically tailored valence triple-zeta basis set (mTZVPP),[3] the D4 London dispersion correction,[4] and a geometrical counterpoise (gCP) correction[5] to account for inter- and intramolecular basis set superposition errors (BSSE). The transition structures were verified by eigenvalue analysis of the Hessian, and connectivities between minima and the corresponding TS were validated by intrinsic reaction coordinate (IRC)[6] following calculations.

Relative energies were produced using correlated coupled-cluster theory within the domain-based local-pair natural orbital framework and including single and double excitations with iterative perturbative triple contributions, i.e., DLPNO-CCSD(T1)[7,8] A DFT-based Kohn–Sham (KS) orbital reference wavefunction using the PBE0[9,10] functional was chosen. Restricted and unrestricted KS reference wavefunctions were used for closed-shell singlet and high-spin open-shell species, respectively. The NormalPNO setting was applied. The one-particle space on hydrogen atoms was described with the def2-SVP basis set, all other non-metal atoms (C, N, O) with the def2-TZVP sets, and the Ni metal centres with the def2-TZVPP and def2-QZVPP sets. Two-point extrapolations to both, the complete pair-natural-orbital (PNO) space (CPS) limit as well as the complete basis set (CBS) limit for Ni were performed, resulting in KS-DLPNO-CCSD(T1)/CPS/CBS(Ni=T,Q) energies according to the following extrapolation schemes:[11–13]Two-point PNO extrapolation of correlation energies is computed for the def2-TZVPP basis set on Ni as:

$$E_{\mathrm{corr}}^{\mathrm{PNO}} = E_{\mathrm{corr}}^{\mathrm{loose}} + F \times \left(E_{\mathrm{corr}}^{\mathrm{tight}} - E_{\mathrm{corr}}^{\mathrm{loose}}\right)$$

where $F = 2.38$ for loose (1.00×10^{-6}) and *tight* (3.33×10^{-7}) TCutPNO thresholds.

Two-point CBS extrapolations of SCF reference and correlation energies are computed as

$$E_{\mathrm{ref}}^{\mathrm{CBS}} = \frac{E_{\mathrm{ref}}^{X}\exp\left(-\alpha\sqrt{Y}\right) - E_{\mathrm{ref}}^{Y}\exp\left(-\alpha\sqrt{X}\right)}{\exp\left(-\alpha\sqrt{Y}\right) - \exp\left(-\alpha\sqrt{X}\right)}$$

$$E_{\mathrm{corr}}^{\mathrm{CBS}} = \frac{X^{\beta}E_{\mathrm{corr}}^{X} - Y^{\beta}E_{\mathrm{corr}}^{Y}}{X^{\beta} - Y^{\beta}}$$

with cardinal numbers X = 3 and Y = 4 for T and Q, and $\alpha = 7.88$, $\beta = 2.97$ for the def2-family. The CBS extrapolation for the correlation energies is applied to results with the NormalPNO settings (TCutPNO = 3.33×10^{-7}) to yield an additive correction term, $\delta E_{\mathrm{corr}}^{\mathrm{CBS}}$, which is defined as

$$\delta E_{\mathrm{corr}}^{\mathrm{CBS}} = E_{\mathrm{corr}}^{\mathrm{CBS}} - E_{\mathrm{corr}}^{X}$$

The final extrapolated energies are obtained as

$$E^{\mathrm{CPS/CBS(Ni=T,Q)}} = E_{\mathrm{ref}}^{\mathrm{CBS}} + E_{\mathrm{corr}}^{\mathrm{PNO}} + \delta E_{\mathrm{corr}}^{\mathrm{CBS}}$$

Relative enthalpies and Gibbs energies are reported at the extrapolated KS-DLPNO-CCSD(T1) level and include the corresponding thermal contributions at 298.15 K from the r^{2}SCAN-3c geometry optimisations, i.e., KS-DLPNO-CCSD(T1) // r^{2}SCAN-3c.

Table S2: Relative energies for singlet–triplet splittings, H_{rel} and G_{rel}, given in kcal mol^{-1}, computed at the KS-DLPNO-CCSD(T1) // r^{2}SCAN-3c level. Spin expectation values ⟨S^2⟩ of the reference wavefunction, and T_1 diagnostics for CCSD are also given.

Species	Mult	⟨S^2⟩	T_1	ΔH_{rel}	ΔG_{rel}
**1**	singlet	0.000	0.010	0.0	0.0
	triplet	2.090	0.011	17.9	16.5
**Int(A)**	singlet	0.000	0.010	0.0	0.0
	triplet	2.073	0.011	8.0	6.7
**Int(B)**	singlet	0.000	0.010	0.0	0.0
	triplet	2.010	0.011	17.0	15.6
**Int(C)**	singlet	0.000	0.010	0.0	0.0
	triplet	2.010	0.011	15.7	15.6
**2^{CO}**	singlet	0.000	0.009	0.0	0.0
	triplet	2.020	0.011	26.4	24.2
**2a^{CO}**	singlet	0.000	0.009	0.1	0.0

Table S3: ⟨S^2⟩ of the reference spin expectation values, total energies E_{tot}, thermal corrections to enthalpy H^{298} and Gibbs energy G^{298} in Hartree, computed at the r^{2}SCAN-3c level. CCSD T_1 diagnostics and total energies E_{tot} for KS-DLPNO-CCSD(T1) results are also given. The corresponding relative enthalpies H_{rel} and G_{rel} are given in kcal mol^{-1}.

Species	r^{2}SCAN-3c						KS-DLPNO-CCSD(T1)			
	⟨S^2⟩	E_{tot}	H^{298}	G^{298}	ΔH_{rel}	ΔG_{rel}	T_1	E_{tot}	ΔH_{rel}	ΔG_{rel}
CO (×6)	0.000	−113.293 607	0.008 301	−0.014 131	0.0	0.0	0.005	−113.158 481	0.0	0.0
**1**	0.000	−4938.824 639	0.916 452	0.793 428	0.0	0.0	0.010	−4934.356 506	0.0	0.0
**Int(A)**	0.000	−5052.126 782	0.926 090	0.795 838	−4.5	5.0	0.010	−5047.519 449	−2.0	7.6
**TS(A,B)**[a]	0.000	−5052.114 760	0.924 254	0.795 144	1.9	12.1	0.010	−5047.495 033	12.2	22.5
**^{3}Int(A,B)**	2.009	−5052.110 003	0.924 214	0.790 233	4.8	12.0	0.011	−5047.482 028	20.3	27.5
**Int(B)**	0.000	−5052.136 692	0.927 015	0.798 935	−10.2	0.8	0.010	−5047.516 405	0.5	11.4
**TS$_{rot}$(B,C)**[b]	0.000	-5052.116 536	0.925 880	0.798 669	1.8	13.2	0.010	-5047.495 170	13.1	24.6
**TS$_{13H}$(B,C)**[c]	0.000	-5052.095 091	0.922 886	0.795 946	13.4	25.0	0.010	-5047.474 974	23.9	35.6
**Int(C)**	0.000	−5052.141 943	0.927 508	0.798 404	−13.1	−2.9	0.010	−5047.520 490	−1.7	8.5
CO_2	0.000	−188.570 063	0.015 255	−0.009 012	0.0	0.0	0.006	−188.333 367	0.0	0.0
$NiCO_4$	0.000	−1961.700 816	0.042 978	−0.004 384	0.0	0.0	0.009	−1960.456 517	0.0	0.0
**2^{CO}**	0.000	−3468.467 363	0.918 209	0.789 945	−89.0	−52.7	0.009	−3464.601 704	−46.4	−10.2

[a] imaginary mode ν_{imag} = 81.8 i cm^{-1}
[b] imaginary mode ν_{imag} = 198.4 i cm^{-1}
[c] imaginary mode ν_{imag} = 1728.0 i cm^{-1}

We include the two TS structures connecting **Int(B)** and **Int(C)**: **TS$_{rot}$(B,C)** (Figure S38, left) via rotation around the Ni1–C bond is 13 kcal/mol above **Int(B)**, and **TS$_{13H}$(B,C)** (Figure S38, right) via 1,3-hydrogen shift is computed 25 kcal/mol above Int(B), but the latter is expected to be significantly lowered by acid/base catalysis. Interconversion between **Int(B)** and **Int(C)** is thus facile.

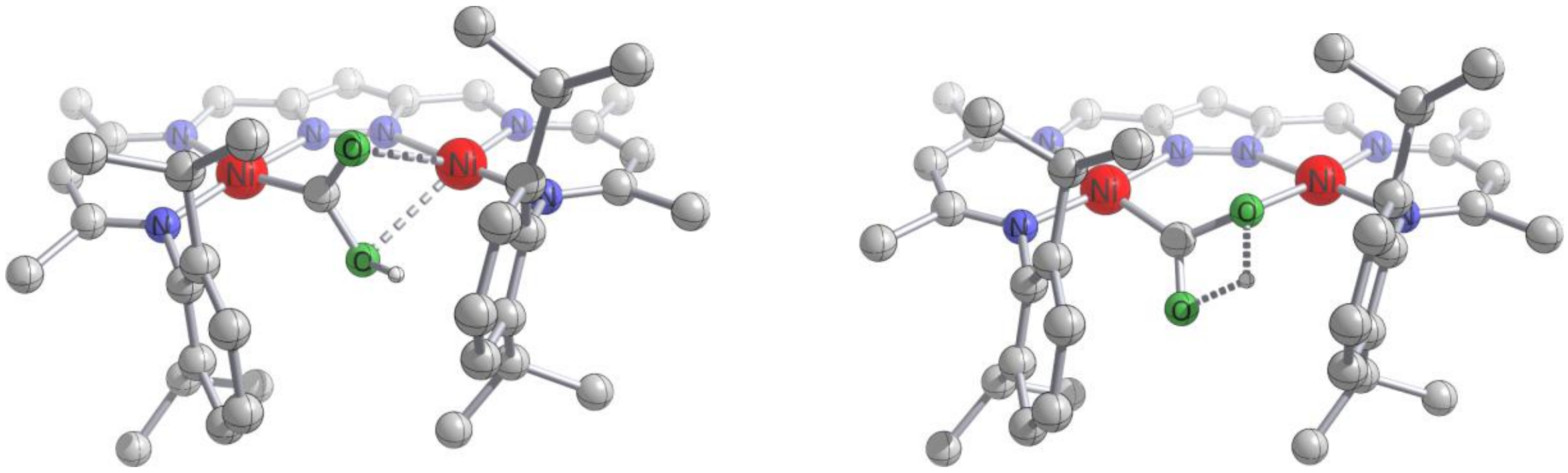

Figure S38: Left: **TS$_{rot}$(B,C)** for rotation around Ni1−C bond; right: **TS$_{13H}$(B,C)** for 1,3-hydrogen shift. The rotational TS is 13 kcal mol^{-1} above **Int(B)**. The 1,3-H shift is computed 25 kcal mol^{-1} above **Int(B)**, but is expected to be significantly lowered by acid/base catalysis.

[1]CO

2

C	0.00000000000000	0.00000000000000	0.03477423331069
O	0.00000000000000	0.00000000000000	1.16522576668931

[1]1

102

Ni	-1.79816554561081	-0.09141079798274	-0.13035280330428
Ni	1.67019707582772	-0.02673032426175	0.07853355278786
N	-0.70028407899922	-1.51408236638867	0.05190679296176
N	-3.10854105349144	-1.45667244943353	-0.10927474091205
N	-3.02099784621874	1.33574468013477	-0.20080555743863
N	0.60636543015982	-1.48061214815850	0.20940925354334
N	3.00990928635458	-1.33017470350080	0.37625207877408
N	2.86063010889021	1.41242292159889	-0.13850122906812
C	-1.13170621647151	-2.77404741701362	0.15980535145025
C	-2.60834420920763	-2.85162642129626	0.01688641049848
C	-4.41708546651823	-1.28291367435665	-0.22165947325853
C	-4.35001993925608	1.20771627347054	-0.21201494261161
C	-2.52818973978310	2.66704423161383	-0.05030829788418
C	1.06439086739976	-2.71967456311450	0.40965527930571
C	-0.02441246071229	-3.60696408289156	0.39069757003963
C	-5.01239853693423	-0.02016231235435	-0.27980189566895
C	-5.33403260235523	-2.48221895204383	-0.27016887998933
C	-5.23114707194031	2.43524537998195	-0.11686322387455
C	-2.16648160152577	3.39971883792867	-1.19919204455065
C	-2.51875991168521	3.24426554179385	1.23632188624259
C	2.54068253269386	-2.72781179831629	0.57387042083937
C	-1.84040697757630	4.74702950929870	-1.03315194925243
C	-2.18064395088423	2.75637045266246	-2.58018429066893
C	-2.19918233141484	4.59800187023666	1.34287622127852
C	-2.87612878326092	2.41406638568921	2.45972932570575
C	4.31464534285184	-1.10857568986911	0.43783026559797
C	4.19217625753411	1.32089283899327	-0.09946785165898
C	2.33808216527569	2.67909296793068	-0.53677718473126

C	-1.87303863871003	5.34479690665172	0.21904123723836
C	-1.34222665801083	3.52871480387795	-3.60139694674761
C	-3.60065171289934	2.58977971718707	-3.15078397281166
C	-1.65950663034542	1.62432434829677	2.96981908052424
C	-3.48996936954756	3.23688411210297	3.59531749692577
C	4.88220203720996	0.14875232686748	0.21678176252450
C	5.25877283859141	-2.24844259585808	0.73904511412737
C	5.04295041835574	2.52694550112292	-0.43403723048939
C	2.32300922035988	3.00639158432384	-1.90768426389345
C	1.94509558926889	3.60151172418452	0.45521593896379
C	1.96292060321349	4.30561090881823	-2.26533072731088
C	2.70755713382753	1.97576583660449	-2.95715604276726
C	1.57799364734590	4.88280645850051	0.04026839016459
C	1.96616411733962	3.22375485504983	1.93164994399394
C	1.60193919349561	5.23737032249247	-1.30149471070513
C	1.49433542026919	1.12143129965547	-3.36035908427585
C	3.35931281280558	2.58907019726677	-4.19994054165549
C	1.09845831801722	4.14345148239500	2.79342198755052
C	3.38622691615142	3.20092413870986	2.52487573898585
H	-2.88503662002490	-3.44624049378430	-0.86742571975408
H	-3.05966210827803	-3.34504450033765	0.89073004616252
H	-0.01286167026136	-4.67851410828164	0.52295671334779
H	-6.09252758025563	0.01053727145245	-0.34218862890623
H	-5.08835553536119	-3.13802462668693	-1.11311081618487
H	-6.37090155277496	-2.16205570180275	-0.37812231966476
H	-5.25560141351013	-3.08386238295945	0.64252473846166
H	-5.27926491927080	2.78673567027355	0.92098456723887
H	-6.24613125816051	2.19368040387353	-0.43857608065875
H	-4.85317716668223	3.26752794328706	-0.71394341112309
H	2.82084658887054	-3.09386274381304	1.57376671643210
H	3.01283923013108	-3.39922526918125	-0.15867845990985
H	-1.55972533399030	5.34301374587179	-1.89442705334991
H	-1.74698682164813	1.75205677015744	-2.46673005347663
H	-2.22171769710405	5.08323321258224	2.31390988720381
H	-3.62204893844692	1.67296864392624	2.14960235697216

H	-1.63160368372999	6.39942704768441	0.32091561324392
H	-1.82396905851822	4.47441122713787	-3.87809164986943
H	-1.24447405611922	2.93799024386250	-4.51801692318027
H	-0.34166613042282	3.74817100264793	-3.22122853616107
H	-4.11934363738202	3.55581878741139	-3.17603578999215
H	-4.20378928575565	1.88197488974712	-2.57994845999919
H	-3.53940390274926	2.21649145899488	-4.17928480752950
H	-1.21150681845993	1.02278292112944	2.17143976480848
H	-1.95937693847053	0.95096648927518	3.78095692602542
H	-0.89267662806783	2.30295878804249	3.35635756885188
H	-2.75162918838910	3.89585296743913	4.06648911876969
H	-3.86726265534242	2.56743589687280	4.37539374818332
H	-4.32277121981922	3.85653233727703	3.24469443922322
H	5.96138099578124	0.21535786370137	0.26533803898202
H	5.02677572458327	-2.71655004853436	1.70241010958286
H	6.28759453505755	-1.88847842740086	0.77412882691714
H	5.19721310648504	-3.03127803346787	-0.02559916301125
H	5.06590553883084	2.68558430364287	-1.51878695585688
H	6.06816637917081	2.36977931566745	-0.09304527227095
H	4.65319252358685	3.44522463209372	0.01071000821769
H	1.97793574765940	4.60110199078111	-3.31011403105609
H	3.43633809123889	1.29459747429491	-2.50356140107831
H	1.26858627885077	5.61912174832000	0.77371076296305
H	1.56129751679629	2.20277145340935	2.00212399228235
H	1.32591177808988	6.24547313375657	-1.59871982510625
H	1.00446865587853	0.68220040096571	-2.48551872475993
H	1.80777770615566	0.31325394507542	-4.03110130855748
H	0.75685574776884	1.73513384092198	-3.88727326514870
H	2.63831329751281	3.15947040546622	-4.79647904860383
H	3.74974080554495	1.79320702701537	-4.84275884101985
H	4.18796657923562	3.25724314733194	-3.94012875855752
H	1.54431666568429	5.14109550210335	2.88648048010190
H	1.02069464039188	3.73240402732877	3.80538664992800
H	0.09115338090126	4.25163784453560	2.38402618001639
H	4.01155918276829	2.42209394655008	2.08564123992975

H 3.32934150121516 3.01161549381822 3.60267983068455
H 3.87763700196802 4.17070130752759 2.37997962738412
O -0.07379477780725 0.90099305973429 -0.20196012314086
H -0.09296260323486 1.86555663466051 -0.15374173848201

^{3}1

102

Ni -1.89254395416121 -0.10287076658501 -0.18061522630306
Ni 1.63124166707507 0.08739405852847 -0.04086553270521
N -0.75589313186699 -1.53318166224206 -0.24621390764197
N -3.18674210034040 -1.48575334457261 -0.11072789403171
N -3.14125831355969 1.30962109724065 -0.10664487755459
N 0.55806335296375 -1.48244444526713 -0.15289575780170
N 3.00588678320991 -1.34339682133769 0.34984847602882
N 2.97032910146522 1.47703813072125 -0.20303880332595
C -1.18057226846992 -2.77607123983026 0.00507855481460
C -2.66239031915668 -2.87167090943447 -0.01622341701387
C -4.50116090387676 -1.32874500969412 -0.16237113657172
C -4.46851776327243 1.16384280725698 -0.11583850127171
C -2.66229469363932 2.64398069361096 0.06087167570030
C 1.02101320548710 -2.69200004288536 0.17340348487435
C -0.06306134315680 -3.58014563724838 0.27905062985107
C -5.11418159658899 -0.07242576260872 -0.19036473186047
C -5.40521097892344 -2.53869814160986 -0.17462119556928
C -5.36415846353465 2.38035428908871 -0.01174994560189
C -2.25789336167976 3.37342884063014 -1.07513154904240
C -2.66880947155691 3.21875557170549 1.34844477026668
C 2.49773343613777 -2.72866697843843 0.41456305017370
C -1.90648152190293 4.71360137199974 -0.90026260530443
C -2.24112272457282 2.72558503042345 -2.45125133193157
C -2.32969472079901 4.56663996275356 1.46505264711232
C -3.02599940244376 2.38340579290079 2.56713598453176
C 4.30543735677710 -1.14702554198502 0.49860615574743
C 4.28879089748016 1.31421569679386 -0.06340168542308
C 2.51554354752570 2.75334211829149 -0.64646824165894

C	-1.96080927272547	5.31035726938608	0.35148758602751
C	-1.29267486051505	3.42679165280458	-3.42464787351794
C	-3.64321422367920	2.64910906247678	-3.07885434467117
C	-1.79648886239652	1.60658051896944	3.06600605090457
C	-3.64414180570739	3.19795366254401	3.70607572258439
C	4.89801011346169	0.11625009673862	0.33436316052715
C	5.22746456660054	-2.30124991747517	0.81864935340437
C	5.22693769841054	2.45388513861154	-0.40273119614880
C	2.53297537867998	3.05610446337890	-2.02146980220145
C	2.06117132678433	3.68191241754358	0.31195575335639
C	2.12721561889894	4.32998976014698	-2.41895912972010
C	2.94671857212072	2.00715387180211	-3.03949802593863
C	1.65557818757703	4.93888258459423	-0.13854485697773
C	2.05358693242219	3.32850219219741	1.79250653117867
C	1.69879573329854	5.26680872090115	-1.48743501162627
C	1.72087429004984	1.20981186627493	-3.51453686845249
C	3.70700049750953	2.58655035431112	-4.23606684083534
C	1.06223608694661	4.16459513871370	2.60254360609231
C	3.44791301898946	3.45109894152465	2.43034918470668
H	-3.00470204895819	-3.48001387592573	-0.86787699966326
H	-3.03276102421373	-3.35999936982285	0.89746660957563
H	-0.04457700866608	-4.63026342004383	0.53237295977892
H	-6.19589712642487	-0.05581589328263	-0.22741214561184
H	-5.20297133911583	-3.17802692132711	-1.04154801067905
H	-6.45084803288587	-2.23132081986140	-0.21391710273830
H	-5.26276052730341	-3.15271547337899	0.72179238077983
H	-5.41450729690522	2.73182452526719	1.02580486530402
H	-6.37694713092379	2.12772079227807	-0.33188079074389
H	-4.99643703792681	3.21697068428921	-0.61010162436846
H	2.70098674661345	-3.18610836111867	1.39625280329642
H	3.00018217830795	-3.36115774107472	-0.33492507405168
H	-1.58568991545976	5.30097526035994	-1.75376346254915
H	-1.88078127944092	1.69605458907178	-2.31178854327910
H	-2.35335134099895	5.04659865469964	2.43877380784026
H	-3.76486972063011	1.63589228924815	2.25581802969362

H	-1.69804192156949	6.35879757239905	0.46347628887568
H	-1.67775707362435	4.40782098389401	-3.72821231652181
H	-1.19264034274120	2.82492856944179	-4.33376106082553
H	-0.29958164574283	3.56460882764111	-2.98853550190666
H	-4.09403328187614	3.64743860197061	-3.13384029107306
H	-4.31314433368368	1.99298863111830	-2.51939307048394
H	-3.57087956155958	2.25565512282076	-4.09908447607914
H	-1.37031672443426	0.98850780387790	2.26875987873023
H	-2.07363248920516	0.95264471735849	3.90080521209938
H	-1.02088176394963	2.29723486247926	3.41396915605331
H	-2.90888322279234	3.85716787041791	4.18138731485550
H	-4.02046648021497	2.52351416228648	4.48224636653742
H	-4.47841940688048	3.81648270274871	3.35698871810392
H	5.97431448061184	0.15505312795097	0.45342163732205
H	4.91732649399548	-2.80926957105056	1.73885319287963
H	6.25539461650045	-1.95841637520356	0.94502280255321
H	5.21294539499378	-3.04951549140828	0.01764333278538
H	5.37803100636931	2.49753853940348	-1.48859689795321
H	6.20173519303884	2.29984445266655	0.06471844871339
H	4.83048480415174	3.42539995114660	-0.10043672419686
H	2.14261666974071	4.59579899732874	-3.47212512050276
H	3.61332154892836	1.29742174528325	-2.53724906978211
H	1.29511036704497	5.67406347035142	0.57298963046583
H	1.74594192413164	2.27474601842594	1.86836305598337
H	1.38423387888871	6.25378181694252	-1.81523542649847
H	1.14914693286493	0.80794773570825	-2.66949984142312
H	2.02975487771147	0.37359650927198	-4.15199981360797
H	1.05003874886244	1.85296414305412	-4.09315862514120
H	3.05788328764042	3.19545051548442	-4.87481121223933
H	4.09950609459285	1.77406368785210	-4.85676027726132
H	4.54785580511805	3.21130845156478	-3.91584582637204
H	1.39354521504237	5.20587172761130	2.69538496218955
H	0.98566852949227	3.76139111190489	3.61787462675971
H	0.06714226695917	4.15857952006015	2.15121725593803
H	4.15823250212827	2.72966251131509	2.02260918650219

H	3.37922355623873	3.27395203723742	3.50957153444612
H	3.84902793618640	4.46032829295074	2.27792922053696
O	-0.25203205065983	0.94974543553612	-0.12532816473657
H	-0.37176523871276	1.90791733712773	-0.07774389548935

[1]Int(A)

104

Ni	-1.76345593617843	-0.08507970319905	-0.16058330099942
Ni	1.69892322350196	-0.02347412336636	0.07106077322669
N	-0.67822052938230	-1.49749062737885	0.17290299700045
N	-3.08693787128954	-1.44467133280532	-0.00353105493977
N	-2.98670587336570	1.34785036715279	-0.32824547726910
N	0.63196253772226	-1.46585529654901	0.28123448701451
N	3.04008627062314	-1.32367362628997	0.37811545501772
N	2.88797102906062	1.40513806903178	-0.20977294301565
C	-1.11547836493786	-2.74503139298254	0.36470959473483
C	-2.59371774435428	-2.82577940099960	0.23977823568954
C	-4.38009766375513	-1.28320642812252	-0.23387210952580
C	-4.31190952993647	1.20497415233018	-0.38234941459092
C	-2.51159951683256	2.67856114580238	-0.11324308375927
C	1.08785172172200	-2.69450427351594	0.54150454803748
C	-0.00692314228774	-3.57212514802251	0.60930012336905
C	-4.96343611047867	-0.03009820288878	-0.43785434916243
C	-5.29384310994649	-2.48470132324196	-0.26774640397473
C	-5.20963815429644	2.42215384096042	-0.32522424676699
C	-2.10876143447079	3.46183138568438	-1.21542865594994
C	-2.55041701023286	3.20069012109308	1.19567124909382
C	2.56834887396601	-2.70508796781296	0.66306172444014
C	-1.77247811379114	4.79524110235138	-0.97428152533952
C	-2.07867213036927	2.88801653914575	-2.62713379908268
C	-2.21089502946805	4.54102700299233	1.37811290359388
C	-2.96110115142953	2.32987215978582	2.37190927081673
C	4.34793533406111	-1.11313689099863	0.36935318623787
C	4.22018715032667	1.31021423457363	-0.19847216588888
C	2.36490314219720	2.68466601514245	-0.56438703557865

C	-1.83238156595037	5.33344601660903	0.30382231737430
C	-1.24724574282829	3.72920803468891	-3.59842253933772
C	-3.47648394434398	2.69477208342157	-3.24060196718346
C	-1.73931268585926	1.62736736052990	2.98651553503390
C	-3.73533849048765	3.09524987745520	3.44987566291674
C	4.91396479592862	0.13469085113050	0.09617369838944
C	5.29658530247884	-2.25385946045134	0.65233087283893
C	5.06715405960849	2.52172266000508	-0.52458467094071
C	2.34214977684599	3.05733249392305	-1.92406645986791
C	1.98968043040960	3.57829083426106	0.45952141396535
C	2.01537072873057	4.37791450304155	-2.23296197128533
C	2.68252336951119	2.04844574841025	-3.01021038244198
C	1.64958300037682	4.88122257395784	0.09280278998739
C	2.00170334888614	3.14459475751152	1.91868666718364
C	1.68311388637758	5.28425055647029	-1.23473123715583
C	1.47154863730940	1.15596338270847	-3.32817133835435
C	3.21614811860553	2.68938932123191	-4.29372636691428
C	1.11752957905179	4.02292959908620	2.80582934550232
C	3.41941145563236	3.10852491476521	2.51551520227315
H	-2.87668438824223	-3.49212525320596	-0.58957747207016
H	-3.04579345335055	-3.23994171439706	1.15407330277642
H	0.00156504389290	-4.63423818923680	0.80328360767262
H	-6.03718139411458	-0.00731933309141	-0.57228743284945
H	-5.02178085358496	-3.15909175282321	-1.08820593911586
H	-6.32795749581950	-2.17143646566244	-0.41455698335490
H	-5.23751341203654	-3.06197239938883	0.66153560069831
H	-5.34707475496099	2.72833558610039	0.71924969817823
H	-6.19249214937216	2.18150388903545	-0.73618599215768
H	-4.79534765027653	3.27982376148481	-0.85698688846366
H	2.87487667191400	-3.01745266915392	1.67345761042941
H	3.01329938047920	-3.42091321313345	-0.04392285826655
H	-1.45604390771828	5.42741801772123	-1.79622078015034
H	-1.60855471372602	1.89673733001590	-2.55319269198669
H	-2.25368993629610	4.97676035506691	2.37195208405653
H	-3.62065948311056	1.54217535685715	1.99052242899577

H	-1.57294914864376	6.37648035036602	0.46363611334530
H	-1.74880542252929	4.67530358427143	-3.83575115959246
H	-1.12543721270886	3.18565250398352	-4.54113526505935
H	-0.25571652330776	3.95324299308236	-3.19852103126086
H	-4.02760320835083	3.64283333152320	-3.24052078213643
H	-4.07002044702894	1.94214985404904	-2.71993547568457
H	-3.37486863851652	2.36683268740901	-4.28114167413744
H	-1.15366277728656	1.10595562880780	2.22227435437752
H	-2.06006594802281	0.89947810332888	3.74070001891835
H	-1.08429918289908	2.35651248882395	3.47462393128452
H	-3.09239494827578	3.79752318288135	3.99190408341990
H	-4.13701503605954	2.39227093872755	4.18728804271730
H	-4.57181111815016	3.66135066836033	3.02544602865942
H	5.99450755586063	0.19477940164788	0.10345475594776
H	5.09942667795375	-2.70770128719074	1.63002040745531
H	6.32794551192335	-1.89969380840899	0.64241237242479
H	5.20165400819406	-3.04669802107875	-0.09870044716785
H	5.04555772503043	2.73055085254585	-1.60051930178421
H	6.10404670917652	2.34366827321910	-0.23352709951805
H	4.70393266505512	3.42188151845731	-0.02246871713179
H	2.03369396867239	4.71042891813958	-3.26602943902808
H	3.46599882410328	1.38686724431477	-2.62125047579427
H	1.35791186652517	5.59675935811556	0.85386418703577
H	1.59855262329756	2.12171752239377	1.94333619563126
H	1.43473947905225	6.30967246072781	-1.49505463029529
H	1.10011342685616	0.64099449929583	-2.43641442727899
H	1.74493133008937	0.39736630179949	-4.07044979721868
H	0.65173950436987	1.75358251684674	-3.73926797983053
H	2.43564078649608	3.24810895839123	-4.82310831712697
H	3.56991885109327	1.90840713891630	-4.97456220855080
H	4.04944840947626	3.37273885876987	-4.09540145876860
H	1.55339067128279	5.01931801920636	2.94806072643968
H	1.02776842729970	3.56774402170379	3.79762573195085
H	0.11536276751627	4.13853755998224	2.38528318354166
H	4.05249866091592	2.35444264254491	2.04413142418976

H	3.36242743091299	2.87247170047064	3.58409552992851
H	3.90331656957387	4.08751012801099	2.41279055516810
O	-0.04301216216591	0.92694326570487	-0.07942576923830
H	-0.03683996880343	1.83608967400311	-0.40616664323377
C	-1.82583587395588	-0.54625185139973	-2.62864358105165
O	-2.84016926436014	-0.75499699556163	-3.10940681035138

^{3}Int(A)

104

Ni	-1.79068169707120	-0.01139779495732	-0.52173658238078
Ni	1.76104189996154	0.02397339362196	0.09343279555816
N	-0.66993615057541	-1.41995442208624	0.18226930974720
N	-3.13942651526102	-1.53225399787990	-0.21589149691733
N	-3.19467517517939	1.36089017581129	-0.26214598135989
N	0.63619744259235	-1.38803729909369	0.36079333074369
N	3.06814822413236	-1.28422011201827	0.50665747045832
N	2.99781466962809	1.40889622990789	-0.23651478799565
C	-1.10081845927575	-2.67874789935491	0.33092526665768
C	-2.56657432741124	-2.85541557282607	0.08048471321679
C	-4.44108822645868	-1.40930550273100	-0.37012583646390
C	-4.50140015936161	1.11695900582214	-0.37666910724273
C	-2.80197082571961	2.70688033799753	0.00622295367050
C	1.08576172800877	-2.61425436622718	0.64227266988854
C	-0.00525567785403	-3.49988931357978	0.63900840227701
C	-5.06742677594053	-0.15751007529143	-0.52839876984071
C	-5.33602747252014	-2.62659728159914	-0.32687130666208
C	-5.47420216471486	2.27111413633069	-0.28245869241009
C	-2.54807148849294	3.59767472368333	-1.05222570616043
C	-2.70820673609833	3.12546057180369	1.35098440737590
C	2.55835565757616	-2.63878168163168	0.83619229118649
C	-2.16552776463551	4.90461257250713	-0.74453258628727
C	-2.70006914395870	3.17703441209188	-2.50222544550891
C	-2.36158884701279	4.45035527656841	1.60705680667157
C	-2.96879538495607	2.14497138955583	2.48124435532442
C	4.37881651936602	-1.09294875720037	0.55286478549818

C	4.32430945276738	1.29471703095472	-0.16575596603007
C	2.47754152613695	2.65384894238519	-0.69753413145109
C	-2.08103035260574	5.33397525885372	0.57075630788062
C	-1.32853176125029	3.07569135189985	-3.17826239044478
C	-3.61339546653289	4.12218944270331	-3.29546879124818
C	-1.66737955273073	1.42610711785447	2.87574690742410
C	-3.61819593559261	2.78813034971509	3.70982284755521
C	4.98105833683649	0.12939023765039	0.23731792075361
C	5.29247864736269	-2.22939714779199	0.94738895509489
C	5.20394475101483	2.46011538607080	-0.56439352779403
C	2.44987161373303	2.92400278608436	-2.08001714915531
C	2.00979961085303	3.58739269386444	0.25049287726801
C	1.96591885947571	4.16379423139475	-2.49717443362672
C	2.91396836781686	1.88373302090201	-3.08664103921422
C	1.52341862343482	4.80852626683947	-0.21989973815949
C	2.06238566552988	3.27080247770813	1.73803641033369
C	1.50785936656717	5.09819467326538	-1.57810993374348
C	1.74229521323034	0.99578870199787	-3.53171999798149
C	3.62036781887513	2.49258220162559	-4.30198728344936
C	1.05981606790080	4.08273784898023	2.55710042280268
C	3.47119554098598	3.46888545474748	2.32257606966660
H	-2.72219554440936	-3.55543639920454	-0.75805635177947
H	-3.05645016383458	-3.30820467070613	0.95718281210635
H	0.00019937661133	-4.56334322946237	0.82914494106611
H	-6.14284192678590	-0.18168955746925	-0.65412774480764
H	-4.99839156387477	-3.39227338778469	-1.03407474569179
H	-6.36719798686538	-2.36287473680606	-0.56582999403217
H	-5.32442842851838	-3.08040765021901	0.67124469487962
H	-5.45244563678226	2.70206959619806	0.72557121005547
H	-6.49205786867067	1.94193065523744	-0.49645828516826
H	-5.20419763922192	3.07873960418841	-0.96817851109043
H	2.81042202744209	-2.90700263910569	1.87441582498067
H	3.01988389112499	-3.39865826050841	0.18809140298087
H	-1.94811196490793	5.60140359078723	-1.54975854565025
H	-3.16233136289077	2.18323510859392	-2.51483548490352

H	-2.30429463896558	4.80084396474986	2.63332054702995
H	-3.66030515705280	1.38201787780442	2.10448620885499
H	-1.79983555723274	6.35977445116864	0.79284031463700
H	-0.85209343361873	4.06028719762560	-3.23352939857604
H	-1.42288872201714	2.68091879205104	-4.19705501651253
H	-0.65460311269443	2.42338971009789	-2.61508380088125
H	-3.16824497114951	5.11792550781644	-3.39455465225749
H	-4.59113352129807	4.24039542070314	-2.81773209323504
H	-3.77329643920341	3.73026523859234	-4.30601540602352
H	-1.14505096960087	1.01942732751941	2.00353511532902
H	-1.87662844980845	0.60794680102658	3.57440624522878
H	-0.98292174804625	2.12706194511412	3.36683392982229
H	-2.92847363956961	3.46438652591197	4.22670788918484
H	-3.90337867052632	2.01092686373644	4.42678443153750
H	-4.51669636711627	3.35595233187283	3.44421220916982
H	6.06160834455643	0.17165801753208	0.28890840141801
H	5.04380080823773	-2.61541258054007	1.94250286076030
H	6.33061557273690	-1.89549812078493	0.96075222502201
H	5.21283558617305	-3.06763811420222	0.24561658087154
H	5.30766038901922	2.50049406120231	-1.65556939131779
H	6.20210656278574	2.34257927902034	-0.13714717298787
H	4.78870645050316	3.41986744003025	-0.24989549495163
H	1.94032583176602	4.40157745277453	-3.55670453752699
H	3.62906631516756	1.22649268760262	-2.58102642484416
H	1.14331830159195	5.54227680966286	0.48263461578009
H	1.80171877749556	2.20770942491671	1.84414274992866
H	1.12759786072798	6.05649880544492	-1.92157551232011
H	1.23823926422744	0.55655081484360	-2.66358476290684
H	2.10037985359309	0.18323002613624	-4.17428802000524
H	1.00863629441563	1.58127466539990	-4.09706945425201
H	2.92247756274388	3.03563422157429	-4.94882084952522
H	4.06966214358614	1.69843222296821	-4.90768398170969
H	4.41285915251575	3.18762443640034	-4.00372782691516
H	1.34247591524228	5.14120491789055	2.61223214967715
H	1.03253178146311	3.70324475165080	3.58415101904660

H	0.05419730388764	4.01410487910056	2.13615629365576
H	4.19576531070250	2.76628623731183	1.90651209468238
H	3.44683124133773	3.31553813698925	3.40735057164654
H	3.82430846143437	4.48988984510773	2.13329361575569
O	0.13076259652055	1.01964808318373	-0.20557082203424
H	0.24069852032388	1.95411945676446	-0.42100467705914
C	-1.59916028734108	-0.18582092544795	-2.36996179541061
O	-1.54955426850675	-0.42442138899330	-3.48232779625770

[1]TS(A,B)

104

Ni	-2.03768087545980	-0.07784177261444	-0.42693040054560
Ni	1.73662915213903	0.07436278416550	-0.03432374691265
N	-0.78361318880331	-1.36518271969508	0.05106173750837
N	-3.27299848835294	-1.44601449450451	0.08565659375217
N	-3.34954518685914	1.27144703187821	-0.43185389654100
N	0.54926812561603	-1.33799868099024	0.07312751249344
N	3.01935907906312	-1.27361529743955	0.31793391327828
N	3.01227977883948	1.45201235948933	-0.25963315794369
C	-1.20390902131471	-2.57972246228071	0.44578048116182
C	-2.67795307313171	-2.73015666573584	0.49956806468506
C	-4.58553497556025	-1.36896114828615	-0.05797515057190
C	-4.66287286861770	1.08473020121212	-0.40591691762897
C	-2.83343687942118	2.57209090657907	-0.15410709877124
C	0.99359709859054	-2.53210679801257	0.48608539620417
C	-0.09525940854387	-3.37468421617602	0.73987215262189
C	-5.25771492751116	-0.17792881872625	-0.35968957196787
C	-5.42917164435739	-2.60684531748977	0.13570508325561
C	-5.57404074410774	2.28581479692780	-0.35024808765916
C	-2.51259872657537	3.45447262031273	-1.20186724829832
C	-2.72066562708185	2.96237964179880	1.19877509097850
C	2.47103202734366	-2.61254730211858	0.60625957189755
C	-2.08239213972826	4.74011392028353	-0.87252785938886
C	-2.63810443434999	3.05599169537785	-2.65821157241714
C	-2.32041040581765	4.26729592640186	1.47350453105158

C	-2.98857802934351	1.98565318542805	2.32898569395060
C	4.33431798510659	-1.12305294453136	0.37563238006688
C	4.33628111169649	1.30333208918876	-0.19273359760239
C	2.52836071114722	2.73046845290292	-0.66528404480831
C	-2.00029010176532	5.15039666466046	0.44938723283383
C	-1.26276454940683	3.08696946211881	-3.33591294960522
C	-3.62836419752619	3.94564842381955	-3.42338458706483
C	-1.66035863123300	1.39458514951137	2.83135836570743
C	-3.78104043523367	2.60476351035076	3.48556585611195
C	4.96744373186525	0.09951413205972	0.12886798010580
C	5.21506380115822	-2.29954972968244	0.72353848269382
C	5.24371338257481	2.47910059750313	-0.48909568455821
C	2.53571811846049	3.06350926337592	-2.03539498495365
C	2.06502770867115	3.63342542015533	0.31507441019201
C	2.06765947227255	4.32411290893871	-2.40849829930318
C	3.03575042109175	2.07405764807952	-3.07785365667692
C	1.61226553746402	4.88280445725593	-0.11178852278774
C	2.06990919714448	3.24735710399927	1.78718341487726
C	1.61194032638969	5.22672969755932	-1.45776867553728
C	1.90466745956845	1.15639837133744	-3.56000637283634
C	3.72676624037131	2.74885816956292	-4.26646103985770
C	1.12511605581949	4.10199904120652	2.63239180047352
C	3.47660115418300	3.29527340461732	2.40690215867424
H	-3.01103347829886	-3.54033932171154	-0.16673153379705
H	-2.99521088243296	-3.00302366228953	1.51937903608305
H	-0.08246282111867	-4.39448346627116	1.09498758848661
H	-6.33619860058380	-0.22226819012589	-0.43547547153491
H	-5.17084097266319	-3.37603155051755	-0.60136666397567
H	-6.48701742034458	-2.36753736249127	0.02343304720154
H	-5.27795041869720	-3.04352837063456	1.12898139427825
H	-5.57934910326756	2.70000725471409	0.66522452943409
H	-6.59524944105569	2.00038633559622	-0.60941679271173
H	-5.23518918530708	3.08383184569579	-1.01424234206113
H	2.74930642680253	-2.94312924329511	1.61911231572441
H	2.86780694988682	-3.36614937074368	-0.09316338938309

H	-1.81967238108959	5.43494118620510	-1.66553397759022
H	-3.01654958760227	2.02817761344020	-2.69064840559245
H	-2.24978973703361	4.59921163698417	2.50523904494560
H	-3.58527844415193	1.15814851576039	1.92927169480726
H	-1.67861554454348	6.16107700930414	0.68535892412750
H	-0.88854726454917	4.11351685804368	-3.41933781556486
H	-1.31782434688579	2.65987002452575	-4.34388742250920
H	-0.52837041792740	2.51947094410773	-2.75833648956266
H	-3.29100392841510	4.98767442714153	-3.43939952840426
H	-4.62766690515268	3.92468505407551	-2.97768017431892
H	-3.71396300901927	3.60625690211974	-4.46155748163652
H	-1.06386803645050	0.99044428003315	2.00546950031627
H	-1.84891977950239	0.60100749237889	3.56371207351438
H	-1.05826451424310	2.17255897207470	3.31466071804955
H	-3.19374434545363	3.35291446774016	4.02893857461518
H	-4.05576344576174	1.82621820690281	4.20518966608618
H	-4.70032309753632	3.08658978351899	3.13516257470598
H	6.04828209900408	0.11484661327976	0.18815491853320
H	4.97824986234122	-2.69407906534352	1.71864086071890
H	6.26485197698576	-2.00384189894161	0.71724370217827
H	5.08735862524571	-3.12230651856429	0.01056982446740
H	5.34777280046200	2.62319815886145	-1.57116375973301
H	6.23889752331243	2.29677386147991	-0.07795067794294
H	4.85299260212887	3.41392013438861	-0.08157514172476
H	2.06419885988874	4.60762956329906	-3.45687511184055
H	3.77139218413795	1.42454113905071	-2.59273412849068
H	1.24461216269496	5.59809178171030	0.61601675318741
H	1.71342228526005	2.20873196567370	1.83168101111903
H	1.25292294368755	6.20513889905704	-1.76597156359600
H	1.41785009945355	0.66454983582340	-2.71129747667430
H	2.29870003674567	0.37899623783487	-4.22458751517395
H	1.14953312364783	1.72627380428735	-4.11169976087281
H	3.01270378221172	3.28478745739495	-4.90161296120423
H	4.21020115576894	1.99209109143295	-4.89343900875023
H	4.49024350572262	3.46374928767025	-3.94021767793847

H	1.50055141764477	5.12612431550117	2.75129004802351
H	1.04064293674392	3.67142819375350	3.63589335464451
H	0.12664472241876	4.14608214894000	2.19119722930276
H	4.14587969320464	2.55065674556868	1.97240728846990
H	3.41226833578924	3.09433834154948	3.48234566083917
H	3.92393446138692	4.28838070231078	2.27648395797123
O	0.18884944035583	1.14369630817214	-0.08763562140876
H	0.30342862800654	2.01554133550750	-0.48712431431762
C	-1.39527212970700	0.04177970734813	-2.22917813304831
O	-1.09645751861928	-0.48420908910415	-3.20228573080940

^{3}Int(A,B)

104

Ni	-2.07904135861532	0.04274264554707	-0.20476524879328
Ni	2.03512203689170	0.17884292594052	0.50541612148563
N	-0.71628609984275	-1.24185911805832	-0.19292587331818
N	-3.22462986199224	-1.45509870232640	-0.33369706250906
N	-3.50481786512683	1.29130608356848	-0.15532910970032
N	0.62208532512187	-1.16865048482038	-0.07377641836091
N	3.22186588098253	-1.34993360792769	0.30312562945407
N	3.43809072858124	1.48194967174342	0.07687820633352
C	-1.09528342016046	-2.53416233789977	-0.24710494899706
C	-2.54904922198024	-2.76555368586922	-0.37790971943932
C	-4.55007949874258	-1.44315335167008	-0.41043636176571
C	-4.80441481281976	1.01656140554416	-0.22357117414508
C	-3.10984219111551	2.66363646455709	0.01799066681967
C	1.08805457574071	-2.42552543155781	-0.03604967305510
C	0.03179452917723	-3.33925882775969	-0.14944604243937
C	-5.31121582919751	-0.27382878836248	-0.36490165188365
C	-5.30138816603119	-2.74598529481281	-0.53987233650319
C	-5.79220647182815	2.15367418975994	-0.13725711529071
C	-2.89734786502921	3.46474156059977	-1.11934508716693
C	-2.84865420163347	3.13621156539891	1.31756290496018
C	2.55499425441565	-2.64160788327558	0.18076777606093
C	-2.33503247591917	4.72664818381975	-0.93280256225626

C	-3.21228940134312	2.97378213178249	-2.52280378929478
C	-2.28984557576272	4.40766559547328	1.45021993066871
C	-3.14234453501471	2.30525765595212	2.55365671768845
C	4.54266023591229	-1.29116191207301	0.35840347833901
C	4.73818808244080	1.20261319172605	0.06805278459952
C	2.97453052389080	2.71744207722655	-0.45898465184157
C	-2.01574305091977	5.18922344401300	0.33707724689399
C	-1.93728226228838	2.55879900668825	-3.27359522207173
C	-3.99051445690393	4.02174915965232	-3.33080822808803
C	-1.85678556552675	1.92434244268754	3.29954706992227
C	-4.11367771285212	3.03603798264470	3.49284840379498
C	5.25416621603574	-0.07705243743699	0.32744401143006
C	5.35315860578716	-2.56601673197003	0.40330109250964
C	5.73035691705337	2.27887392648081	-0.30758732650205
C	2.79412406381822	2.82525131531055	-1.85618214563124
C	2.60705725652124	3.77546694342842	0.39476027017980
C	2.23185591284870	3.99236372813386	-2.36988874684017
C	3.13331384774753	1.67545858095150	-2.79045172860987
C	2.06195987594726	4.92992565866229	-0.16805295010558
C	2.78282056888814	3.68919348394559	1.89844966984085
C	1.86685047254047	5.04232414571055	-1.53660845460175
C	1.86260207685006	0.89286642684448	-3.15454202279991
C	3.86568787795971	2.13540997458402	-4.05657027566824
C	1.45556895779012	3.94814845857059	2.62598549854042
C	3.86624083399592	4.65583483679038	2.39863078689806
H	-2.77342447764644	-3.28416000837200	-1.32319012108388
H	-2.90536542795498	-3.41443912783547	0.43648969246480
H	0.07998613237776	-4.41863852565534	-0.14441999464919
H	-6.38638088619822	-0.37978925370922	-0.42260599519511
H	-4.99086264829876	-3.29931307036516	-1.43299115987387
H	-6.37371502643852	-2.56093306273759	-0.60713585498385
H	-5.11899192328989	-3.39543857326478	0.32357258765561
H	-5.63364929076521	2.73595548232165	0.77538929161356
H	-6.81451186285350	1.77289874437582	-0.14465408690672
H	-5.66709424721192	2.84647446433415	-0.97615282135560

H	2.68798474762289	-3.25409825488582	1.08946455366395
H	2.96581903313245	-3.23317170621448	-0.65400437026235
H	-2.12570392039158	5.35170985805388	-1.79612735747424
H	-3.84477573581584	2.08297085212968	-2.43735316972217
H	-2.05040605700768	4.78375993766210	2.44128150954460
H	-3.62631140768149	1.37677043430159	2.23010946037795
H	-1.55598134391598	6.16575120623322	0.45842273419236
H	-1.20174192881571	3.37156942653747	-3.27280009865356
H	-2.17341053230580	2.30436705959011	-4.31300204182885
H	-1.46664911080923	1.68402393971272	-2.81472800929034
H	-3.36638235829493	4.88684443273778	-3.57838846880701
H	-4.86367462264495	4.38749455732630	-2.78026620391124
H	-4.33560850702291	3.58691014115674	-4.27490046205667
H	-1.13829376127000	1.39843134626424	2.66023549139300
H	-2.09382044599226	1.27463264566041	4.14975612109817
H	-1.35865792270648	2.81863819780030	3.69290380598119
H	-3.65683899614291	3.93997410478309	3.91050151345590
H	-4.38696216994665	2.38615955953444	4.33125922022823
H	-5.03340875085295	3.33553206115878	2.97903496456352
H	6.33428965147883	-0.15577302664631	0.37077193757485
H	5.02440224961248	-3.21686348396660	1.22170331696152
H	6.41421582694374	-2.35062690143239	0.53759350941562
H	5.23834915437571	-3.13485909621525	-0.52731195470391
H	5.79016781338267	2.37161452096089	-1.39942211564442
H	6.72793989907552	2.03074918936514	0.06181852202899
H	5.43098448779538	3.25706738370116	0.07682148416484
H	2.07536274869743	4.08362781104853	-3.44149808173622
H	3.79622969322654	0.98514501631958	-2.25801895742444
H	1.77731481315670	5.75422270982510	0.48090825675280
H	3.10991641236235	2.66890872242616	2.12996756471524
H	1.43266310275228	5.94638641797174	-1.95465770087871
H	1.38137546781561	0.48546252749462	-2.25887827018150
H	2.09950700472977	0.05463361751290	-3.82016580027531
H	1.14246509723004	1.54505587704325	-3.66342547361982
H	3.21280021107862	2.71447609870004	-4.71887297280835

H	4.21533150119191	1.26608303961692	-4.62428902469475
H	4.73304532614108	2.75837109436448	-3.81322173572697
H	1.14023732699124	4.99237212917735	2.51544489947510
H	1.56046954058275	3.74796641234526	3.69936099024962
H	0.66405299456207	3.30779573347274	2.22707510468949
H	4.83387698060103	4.45665839384374	1.92771568603449
H	3.99146282820575	4.56199479851268	3.48349560884801
H	3.59517830403676	5.69502916866447	2.17916883942210
O	1.00020342457144	0.68015436872022	1.97769305866877
H	1.48028694924782	1.21254945444849	2.62049999019927
C	-0.85296382538281	1.35894373852940	-0.25850295175891
O	-0.08613151861364	2.17869783464263	-0.43962915666623

[1]Int(B)

104

Ni	-1.98299678082007	0.02366998403991	-0.35931834831830
Ni	1.95691192920357	0.08304698932595	0.14379053208373
N	-0.68296596364434	-1.27876838887648	-0.04270244451904
N	-3.19654517758010	-1.45008494112810	-0.20447561663955
N	-3.34920137566403	1.34903960404784	-0.30407022288503
N	0.65060416301684	-1.25614628941681	0.14737163745871
N	3.13657574515170	-1.31399799765469	0.52581938245641
N	3.32390581480819	1.39017044907738	-0.02392786252936
C	-1.10491601707066	-2.54959319764845	0.09789412471171
C	-2.56463648589345	-2.76443532765202	-0.05811881433193
C	-4.51521653032932	-1.39527657993418	-0.31702010357736
C	-4.65473784410047	1.09044457356645	-0.21373288175322
C	-2.93984867805969	2.69263346369477	-0.00828640258283
C	1.06649229679696	-2.50676720983536	0.40587078678946
C	-0.02013907413917	-3.37786047853943	0.38591966687441
C	-5.21410073990211	-0.18549130658803	-0.34400333449055
C	-5.32379104916074	-2.66915904585056	-0.37194316196630
C	-5.62610336255028	2.19363359411636	0.15153221174564
C	-2.79162678367328	3.62976290612966	-1.05070443052629
C	-2.71628064863240	3.04806862778902	1.33610983024158

C	2.51899148844597	-2.64603807023275	0.65211185382278
C	-2.39833895046671	4.92590383458780	-0.71344422851336
C	-3.06767540358111	3.26617479013161	-2.50170113537503
C	-2.32872386856827	4.35787726813807	1.62033807098511
C	-2.87631269526100	2.04246230350780	2.46428124029453
C	4.44117995629702	-1.21505324558304	0.74685814199839
C	4.62824270723231	1.18588315409970	0.15117069906748
C	2.92728317485836	2.64343179768255	-0.58851427008922
C	-2.16743029592084	5.29159924208081	0.60631945928914
C	-2.12564659992030	3.98443211902579	-3.47641184981985
C	-4.52592699951066	3.54904959541505	-2.90045323446316
C	-1.50770343460945	1.52541060525914	2.93048457800007
C	-3.66827542364515	2.61016825629219	3.64884481316643
C	5.15911155010490	-0.02774870917139	0.58751432655964
C	5.21927333849800	-2.43809578818433	1.16988834868468
C	5.61676722435051	2.27658622979503	-0.19377194443228
C	2.97105354914195	2.81139203647674	-1.98734191918462
C	2.43581952340509	3.65674392342164	0.26593100108071
C	2.50826753699859	4.01491470059652	-2.51795640034865
C	3.45403775218558	1.70108187070703	-2.90411465877463
C	1.98850917053257	4.84405199183990	-0.31817707484829
C	2.37010083161715	3.44196982879366	1.77100929728695
C	2.02129454349684	5.02086503567596	-1.69502227313461
C	2.26994925807270	0.87390130659620	-3.42710239293685
C	4.30322687216173	2.22604153500068	-4.06814202732867
C	1.26448347308998	4.26114001967685	2.43905712613587
C	3.71802054458067	3.73270701476831	2.45106159014612
H	-2.75937631837884	-3.40395958296195	-0.93503693819739
H	-2.95428397630443	-3.30604845141441	0.81894352308366
H	-0.02464548070060	-4.44365222094743	0.55972106294576
H	-6.29552116381432	-0.24341787676566	-0.35952200441103
H	-5.01214830712776	-3.30079475216944	-1.21201765135151
H	-6.38510919643693	-2.44588329647417	-0.48808406819550
H	-5.19676645493227	-3.26121707136523	0.54193643939342
H	-5.71459181520716	2.23813506677388	1.24463120660061

H	-6.61789429452611	1.96869773487553	-0.24875080655308
H	-5.31293848028926	3.18147334580149	-0.18571700675176
H	2.69452653818311	-3.06574878946278	1.65455102736178
H	2.96155781332030	-3.34881692859482	-0.07129189400253
H	-2.27095237235959	5.66473360906152	-1.49882593737064
H	-2.89670650954128	2.19017871315005	-2.60175326455658
H	-2.15244470300773	4.65227420613323	2.65164542637682
H	-3.43256819064953	1.18349106336705	2.07395045133167
H	-1.86439642827839	6.30773587978028	0.84465139783061
H	-2.39196616115950	5.04219194864521	-3.59080047665253
H	-2.20343952715451	3.52358672499840	-4.46695019701085
H	-1.08495519337256	3.91600618566801	-3.14931319932454
H	-4.79549119247765	4.59062126413093	-2.68644371685270
H	-5.22905526383561	2.89645794279075	-2.37767223477882
H	-4.65559820242573	3.38073350420919	-3.97575252346504
H	-0.95021637175823	1.08254799060050	2.09959640747900
H	-1.62940423434021	0.76574532191956	3.71142326499391
H	-0.90754784549390	2.34579572312564	3.34085098247038
H	-3.10209920275017	3.37951838751663	4.18555675919326
H	-3.89290330680257	1.81311322805058	4.36615589925930
H	-4.61397437180900	3.05724155974678	3.32453575733712
H	6.22698364299337	-0.06701738253074	0.75919009518745
H	4.84636155314767	-2.83878120948547	2.11945764429259
H	6.27364889859985	-2.19052286524579	1.29581651310999
H	5.14165546083122	-3.23951012910024	0.42669915780885
H	5.83488013782304	2.24828675086681	-1.26871153076002
H	6.55539146448755	2.11708228834835	0.34144913063567
H	5.23840884850429	3.27588205530868	0.02834429769623
H	2.51392869819093	4.16284806143602	-3.59355566517340
H	4.08165433314553	1.02305837017613	-2.31534365968922
H	1.58543177573889	5.63259811578677	0.30819892121420
H	2.14241671307823	2.37940677135498	1.93232024155099
H	1.65066803335306	5.94468624302487	-2.12969212064319
H	1.72410378164720	0.40000955578732	-2.60446006594168
H	2.62784252856456	0.08685243085185	-4.10053688248117

H	1.55997558619461	1.50706582213418	-3.96944004282447
H	3.69864144696961	2.77452793403023	-4.79831338210413
H	4.76554422664479	1.38667651358016	-4.59794977353986
H	5.09891269530021	2.89348956371227	-3.71970532534039
H	1.50690356378996	5.33030267989365	2.45972854564601
H	1.14661849178476	3.93779121795527	3.47860950492616
H	0.30529928720079	4.13419566133675	1.92887393177056
H	4.49488093764200	3.02953602073422	2.14237458186305
H	3.61078652714314	3.65182433317786	3.53838131896142
H	4.05582043490730	4.74966400084862	2.21840200076176
O	0.51794622746001	1.39639187930826	-0.01779080052803
H	0.91777645924574	2.22329409654060	-0.36138727722948
C	-0.66651225330995	1.19115606157676	-0.95061421168485
O	-0.55036742499152	1.77355583334128	-1.98775546717845

^{3}Int(B)

104

Ni	-2.02520513739468	0.02454282323546	-0.39029091183198
Ni	1.95576334177746	0.16951211743749	0.19747304038207
N	-0.71664804806069	-1.27060423798698	-0.10237240426196
N	-3.22867100184941	-1.46227602586603	-0.22943384233224
N	-3.39186579617677	1.33929026523587	-0.29829054610050
N	0.61859270006613	-1.21066747918395	0.12735572844367
N	3.16145878872440	-1.32910818678068	0.63963857995638
N	3.38045136360922	1.42303001452336	-0.05404370072382
C	-1.12527942125494	-2.53374467295392	0.07760542881248
C	-2.58086682807768	-2.76923412597146	-0.09061163621440
C	-4.54816467910005	-1.41463134218024	-0.32383106045881
C	-4.69542866364804	1.07528797968696	-0.20322952828524
C	-2.97571892615888	2.67872835888075	0.00540674009323
C	1.04198673083684	-2.45117949635845	0.45148203927879
C	-0.03134115617986	-3.33446554021675	0.43201141199303
C	-5.24957313498068	-0.20423752776206	-0.33688455765433
C	-5.35281443552434	-2.69093904158813	-0.37115366473349
C	-5.66943228344598	2.17471631110554	0.16428086377144

C	-2.79126755732146	3.61088750403271	-1.03567023743375
C	-2.75852483638525	3.02904319372273	1.35204662988417
C	2.48957918042858	-2.62153972471636	0.78775110246050
C	-2.36560132952864	4.89590501034242	-0.69454187502351
C	-3.06420537381767	3.25209157967502	-2.48794027932500
C	-2.33960668713030	4.32798438429661	1.63996400369873
C	-2.94204924443987	2.02599128495383	2.47868458558226
C	4.46757470623085	-1.25566186428110	0.78025549658476
C	4.68820855369762	1.16706932208388	0.11296270873143
C	2.99764356711448	2.68014363853908	-0.61089433344407
C	-2.14010506609507	5.25610882759169	0.62763600200682
C	-2.11069681170039	3.95875658182320	-3.45899358624537
C	-4.51868311672461	3.55136528989174	-2.88768689777384
C	-1.58291609067853	1.49270785331657	2.95472723709767
C	-3.73557425698637	2.60505056523681	3.65666028375365
C	5.18399879726490	-0.05735195555032	0.55904016721787
C	5.26687721408682	-2.48010922918695	1.15686757945962
C	5.70572958997499	2.22791432605789	-0.24133026914541
C	3.03048903634341	2.85363336662083	-2.00902621838876
C	2.47544735149586	3.67688152888660	0.24500013703694
C	2.53606108429911	4.04643698110458	-2.53618787403017
C	3.51559752587923	1.74863108084661	-2.93181894119526
C	1.99603088717007	4.85335230940876	-0.33490266275332
C	2.40822744965212	3.45211052671514	1.74818781148398
C	2.02483303869961	5.03839105941563	-1.71132306698827
C	2.33173004235510	0.91257286376169	-3.44060029231036
C	4.34811141032783	2.27996204592511	-4.10470399337721
C	1.30051083314510	4.25876143054837	2.42743220446324
C	3.75721421536405	3.73480594064113	2.42839620975781
H	-2.75924856562831	-3.40504939751155	-0.97428881267093
H	-2.96966873287493	-3.32490800096391	0.77781544870693
H	-0.02911649830647	-4.39163869426896	0.65540456964832
H	-6.33113872806864	-0.26540643187430	-0.34404672727865
H	-5.03931244377610	-3.32668107228452	-1.20746854618125
H	-6.41531440679134	-2.47293027549773	-0.48698916505173

H	-5.22270657110727	-3.27682613544869	0.54646666167770
H	-5.76911151128121	2.21127808374940	1.25664437592378
H	-6.65764733335320	1.95536692441649	-0.24800046835800
H	-5.34993515801114	3.16414546240748	-0.16300811964231
H	2.58545593281421	-3.00685074653731	1.81682365507585
H	2.93760312884339	-3.38261862411660	0.12866138143689
H	-2.20807901132555	5.62992298483223	-1.47884387369187
H	-2.90376472079353	2.17443587532263	-2.58898967548032
H	-2.16415585006254	4.61721212120494	2.67289586181895
H	-3.50542818111838	1.17330305758729	2.08487672165984
H	-1.81081758305459	6.26332397325705	0.86903361545441
H	-2.35880038453033	5.02143917505904	-3.56950780760655
H	-2.19545931967099	3.50216341069003	-4.45082359055255
H	-1.07127325567074	3.86876031893544	-3.13235383619036
H	-4.77418469581172	4.59776250437193	-2.68008852214636
H	-5.22956640860897	2.91168929557409	-2.35909603815306
H	-4.65201561058969	3.37726406335378	-3.96159083603845
H	-1.03092876856912	1.03744045573663	2.12730584938509
H	-1.71864760286981	0.74160694203419	3.74166928279003
H	-0.97157975842651	2.30829005279167	3.35903643684045
H	-3.16375605874737	3.36863104576054	4.19554281769481
H	-3.97486836308294	1.81219360837534	4.37390428417637
H	-4.67328366046627	3.06322809172829	3.32474052055503
H	6.25827689479547	-0.11533665696039	0.68879700926751
H	4.87849883508554	-2.93919585972757	2.07284084644291
H	6.31710734448518	-2.23080414743061	1.31386789750148
H	5.21122819108850	-3.23926853091519	0.36741846392207
H	5.89332622978077	2.21891928682377	-1.32215703473321
H	6.65394957793523	2.03552493417566	0.26499439515793
H	5.35859117824511	3.23282223610631	0.00897772177811
H	2.53238565544175	4.19435713318885	-3.61183957152787
H	4.15573198215398	1.07444323706307	-2.35255543412062
H	1.57017303332241	5.62728905225632	0.29472844182543
H	2.18110491144032	2.38732810764014	1.90179863277595
H	1.63003249528093	5.95365396235047	-2.14249554355317

H	1.78483904171423	0.44842017725552	-2.61316905329061
H	2.68720830060222	0.11835875669975	-4.10701919357141
H	1.61693170551267	1.53780478415816	-3.98543215153967
H	3.73211245478200	2.82852455122043	-4.82513627290046
H	4.80730580856696	1.44456964372547	-4.64362244389624
H	5.14493494323311	2.94996976894845	-3.76368268868718
H	1.53285276190257	5.33009716214358	2.45024139718607
H	1.19400901890789	3.93023074508798	3.46670902466710
H	0.33818089303880	4.12303841787079	1.92527807455017
H	4.53622903125619	3.04334929362977	2.09831907471780
H	3.65886929799722	3.62994482589989	3.51459739629603
H	4.08796683993823	4.75796903273108	2.21408481987482
O	0.28195742646275	1.63135800958160	0.00496075085376
H	0.73535016861151	2.40103948003449	-0.39906530436582
C	-0.71020131009853	1.23434164453207	-0.95441099233542
O	-0.56842599242651	1.65362014826399	-2.07632828601694

^{1}TS$_{rot}$(B,C)

104

Ni	-1.9793843284	-0.0863452281	-0.0230480942
Ni	1.9793843284	0.0863452281	0.0230480942
N	-0.7117909167	0.6550805030	-1.1612909257
N	-3.2296967877	0.6212078666	-1.2858075919
N	-3.2670388946	-0.9760627016	1.0237969475
N	0.6345757148	0.6896423738	-1.1565770755
N	3.1416136838	0.8282875510	-1.2266249265
N	3.3248865434	-0.4798752333	1.2022926824
C	-1.1526367283	1.2761528828	-2.2693205030
C	-2.6315377207	1.3265157719	-2.4253105876
C	-4.5483154026	0.5717230045	-1.1844099595
C	-4.5843312809	-0.9184511541	0.8240071781
C	-2.7642242807	-1.9268361018	1.9683359651
C	1.0427750444	1.3313662197	-2.2626960522
C	-0.0614860381	1.7303527636	-3.0138865110
C	-5.1949164804	-0.1367729935	-0.1635576697

C	-5.4128302402	1.2800305070	-2.1995825932
C	-5.5095623274	-1.7849525216	1.6511075606
C	-2.3818168123	-1.4868162215	3.2529516002
C	-2.6067379377	-3.2698777607	1.5776961768
C	2.5100825587	1.4495496530	-2.4059255866
C	-1.8369855218	-2.4212966989	4.1332022096
C	-2.5648033298	-0.0366460302	3.6711957563
C	-2.0468013264	-4.1640543643	2.4920096966
C	-3.0022409211	-3.7592772749	0.1943912775
C	4.4678872217	0.7925709179	-1.1759675461
C	4.6339492137	-0.3201010115	1.0348010815
C	2.8497136760	-1.0389255854	2.4298196437
C	-1.6654294031	-3.7488077952	3.7596409262
C	-1.5240907803	0.4232413178	4.6956635868
C	-3.9808199357	0.2246037161	4.2091676718
C	-1.7676248670	-4.0358432703	-0.6757838318
C	-3.8934296398	-5.0079113873	0.2596461844
C	5.1874639273	0.2507173127	-0.1120048832
C	5.2644882270	1.3718933545	-2.3216268576
C	5.6048808223	-0.7089886230	2.1251084173
C	2.7000301424	-0.2194411847	3.5656186564
C	2.5228166809	-2.4164073073	2.4627807334
C	2.1775750102	-0.7954754870	4.7237572218
C	3.1046315359	1.2436379249	3.5815890795
C	2.0020447261	-2.9386630897	3.6477341755
C	2.7430754541	-3.3140255930	1.2499409368
C	1.8211801455	-2.1342973272	4.7655968228
C	1.8957502156	2.1799337159	3.7236757882
C	4.1124917600	1.5228348482	4.7099031103
C	1.7644508803	-4.4923207396	1.1867613646
C	4.1874766056	-3.8374813786	1.2004067907
H	-2.9645862813	2.3761458588	-2.4719883860
H	-2.9222795348	0.8618482129	-3.3819516540
H	-0.0711796908	2.2666592201	-3.9512053583
H	-6.2781313862	-0.1362687249	-0.1779650650

H	-5.2131263890	2.3581914289	-2.2048157489
H	-6.4704298420	1.1306651734	-1.9779958850
H	-5.2195192344	0.9112363359	-3.2137868307
H	-5.6580200196	-2.7490049922	1.1502559907
H	-6.4885153518	-1.3075196193	1.7424352682
H	-5.1153650350	-1.9945999696	2.6467837673
H	2.8441814527	0.9535980102	-3.3301281809
H	2.8024296825	2.5083083342	-2.4821804111
H	-1.5310115094	-2.1043855005	5.1251264686
H	-2.4358292689	0.5751316812	2.7705074385
H	-1.9074234283	-5.2035608955	2.2053089327
H	-3.5715179087	-2.9633747905	-0.2963986941
H	-1.2343889741	-4.4599296988	4.4595604641
H	-1.7114055356	-0.0053786250	5.6880501282
H	-1.5682440496	1.5128018577	4.7989268906
H	-0.5130595062	0.1529469271	4.3789875051
H	-4.2088204164	-0.4484017252	5.0447281029
H	-4.7413559229	0.0859616244	3.4369758980
H	-4.0591196148	1.2559332195	4.5717720188
H	-1.1374933520	-3.1468929655	-0.7644962407
H	-2.0760147233	-4.3513425850	-1.6793882491
H	-1.1608964921	-4.8396570765	-0.2414513648
H	-3.3323462282	-5.8831045309	0.6057756416
H	-4.2853662545	-5.2440119508	-0.7359175909
H	-4.7400302897	-4.8677551020	0.9394339686
H	6.2674151392	0.3020747190	-0.1648733621
H	5.0539828449	0.8437043672	-3.2584313692
H	6.3319682623	1.2881749394	-2.1162448537
H	5.0274267373	2.4292204058	-2.4828899680
H	5.8419424025	0.1737035000	2.7299544448
H	6.5403892442	-1.0621145047	1.6836337238
H	5.2093821605	-1.4744537981	2.7940606210
H	2.0380946492	-0.1788881834	5.6076359095
H	3.5890796413	1.4746273179	2.6264794183
H	1.7178470788	-3.9844522298	3.6936730933

H	2.5869443682	-2.7002757756	0.3524682166
H	1.3992174303	-2.5565304788	5.6730810630
H	1.1855606907	2.0447947002	2.9066445848
H	2.2348668302	3.2220618583	3.7352352870
H	1.3660321793	1.9898644645	4.6641451873
H	3.6325596041	1.4541387192	5.6922756526
H	4.5151626934	2.5369038248	4.6121411554
H	4.9477361620	0.8157908780	4.7010717479
H	2.0055116873	-5.2561356882	1.9352671368
H	1.8321947490	-4.9694594587	0.2037944462
H	0.7274336971	-4.1774622655	1.3380087085
H	4.9100262236	-3.0297724147	1.0645978427
H	4.3043434561	-4.5344286038	0.3631388035
H	4.4343859188	-4.3715589849	2.1255507551
O	0.1993885050	-1.5977356712	0.7426488471
H	0.7579653998	-1.7956058169	1.5251052625
C	-0.5175659520	-0.4378714333	1.0661270369
O	0.0137723854	0.3383777037	1.8442561639

$^{1}TS_{13H}(B,C)$

104

Ni	-1.9895482874	-0.0867886030	-0.0231664440
Ni	1.9895482874	0.0867886030	0.0231664440
N	-0.6785492797	0.6664843028	-1.1383716554
N	-3.1967296920	0.8257213633	-1.2057355224
N	-3.4017110103	-0.7399067959	1.1020202204
N	0.6700871531	0.6485458477	-1.1660322493
N	3.1806799603	0.7415322924	-1.2575624277
N	3.3468094673	-0.5349869178	1.1953329021
C	-1.1054021502	1.3116296039	-2.2403135868
C	-2.5712627516	1.4404852253	-2.3820784601
C	-4.5084432891	0.9606520484	-1.0791010706
C	-4.6940763810	-0.4193818311	0.9805995401
C	-3.1079799923	-1.6785021163	2.1463359985
C	1.0849386226	1.2805042480	-2.2746991973

C	-0.0154140527	1.7263289748	-3.0028597059
C	-5.2209368706	0.3964423410	-0.0215807174
C	-5.2957384139	1.7482220939	-2.0992297099
C	-5.7059404486	-0.9893681810	1.9550930387
C	-2.5710074630	-1.2091682311	3.3589656951
C	-3.3387581793	-3.0462428975	1.9201214471
C	2.5535284831	1.3524357517	-2.4421165366
C	-2.2661641335	-2.1447717609	4.3468101914
C	-2.2998446602	0.2721396794	3.5688105159
C	-3.0130061051	-3.9439961455	2.9361843134
C	-3.8644089404	-3.5558077878	0.5880867155
C	4.5057706393	0.6849090335	-1.2218574446
C	4.6591452862	-0.4106183027	1.0015677395
C	2.9292206866	-1.0675453205	2.4558538617
C	-2.4819800208	-3.5010941633	4.1390145089
C	-1.2089993173	0.5409556334	4.6057573966
C	-3.5725246017	1.0507915432	3.9394606476
C	-2.7067755142	-3.9333514697	-0.3485817999
C	-4.8332333183	-4.7345081035	0.7406475067
C	5.2180092872	0.1397408413	-0.1526199361
C	5.3078971010	1.2371207592	-2.3759031526
C	5.6245051864	-0.8398570604	2.0828649484
C	2.8598217553	-0.2135208083	3.5703905836
C	2.5552641415	-2.4271606757	2.5389079076
C	2.4175745185	-0.7449073375	4.7827140286
C	3.2111577676	1.2605070491	3.4650003931
C	2.1191805919	-2.9089378707	3.7741653041
C	2.6000761692	-3.3331884991	1.3135047299
C	2.0502637028	-2.0783904110	4.8863291797
C	1.9394562377	2.1140852117	3.3494924387
C	4.0749550930	1.7486330869	4.6349830681
C	1.6946966755	-4.5635704172	1.4320180902
C	4.0310297209	-3.7885699873	0.9791014908
H	-2.8401581792	2.5039918870	-2.4764194847
H	-2.9076825770	0.9465884667	-3.3087398700

H	-0.0248580543	2.2709159874	-3.9352528217
H	-6.2876239349	0.5777947042	0.0068190669
H	-4.9889201754	2.8007301516	-2.1146794157
H	-6.3612753833	1.7097347933	-1.8698463951
H	-5.1483358905	1.3531141169	-3.1106079077
H	-5.9597786388	-2.0204890535	1.6830833117
H	-6.6229856016	-0.3971576029	1.9265632892
H	-5.3254992662	-1.0188992314	2.9777367043
H	2.8538899119	0.8278571847	-3.3627079852
H	2.8713431370	2.4007684234	-2.5532263378
H	-1.8383894692	-1.8158221182	5.2881233961
H	-1.9460128874	0.6705058496	2.6062992917
H	-3.1686207998	-5.0080988405	2.7837078152
H	-4.4116596143	-2.7405235587	0.1024237986
H	-2.2314816859	-4.2157389813	4.9183134584
H	-1.5444168472	0.2999823741	5.6216551493
H	-0.9520587277	1.6047991753	4.5945143654
H	-0.3010988321	-0.0335711142	4.3948208616
H	-4.0358172482	0.6253389041	4.8378761203
H	-4.3070855686	1.0400385695	3.1308839024
H	-3.3241914971	2.0975913398	4.1484675456
H	-2.0784715971	-3.0659447937	-0.5678035552
H	-3.0980573910	-4.3301681459	-1.2928167050
H	-2.0708034111	-4.6972868464	0.1134345822
H	-4.3168235182	-5.6490839430	1.0520257713
H	-5.3137861108	-4.9474332797	-0.2202925842
H	-5.6159429126	-4.5238386624	1.4775522001
H	6.2982012019	0.1629962376	-0.2148857302
H	5.0791948221	0.7097695775	-3.3091407186
H	6.3751091083	1.1313708835	-2.1788712118
H	5.0938910076	2.2988753375	-2.5422387967
H	5.6727833017	-0.0727113343	2.8645070233
H	6.6266220352	-0.9629234629	1.6670751371
H	5.3177745327	-1.7685528991	2.5686551771
H	2.3466836401	-0.1017529665	5.6557197508

H	3.7831400719	1.4083200032	2.5429606588
H	1.8060959869	-3.9430619285	3.8682279169
H	2.2459929837	-2.7359853123	0.4589579808
H	1.6966774809	-2.4732433361	5.8346008756
H	1.3078682013	1.7772859318	2.5212256818
H	2.2009448384	3.1659375468	3.1868770122
H	1.3520551104	2.0466165000	4.2710848504
H	3.5108565405	1.7709468078	5.5737711150
H	4.4229895480	2.7690334919	4.4419264746
H	4.9515283978	1.1102922085	4.7888390107
H	2.1004411849	-5.2909047797	2.1452985372
H	1.6375782342	-5.0637709140	0.4597771039
H	0.6782529553	-4.3007640546	1.7365649759
H	4.6697819753	-2.9558478153	0.6769727974
H	4.0072484056	-4.5052574984	0.1507427041
H	4.4878790122	-4.2858734343	1.8430264159
O	-0.5460650934	-2.1684155268	1.3222822325
H	0.5824207231	-1.6455599610	1.6625598922
C	-0.6205286552	-0.9802010435	0.8802032335
O	0.5970697780	-0.4552442068	1.2302805659

^{1}Int(C)

104

Ni	-1.95474705482092	-0.00407996313866	0.14948504413126
Ni	1.96582564040464	0.05368495949197	-0.09654802473116
N	-0.66145070148214	-1.32651617297341	-0.09886650689181
N	-3.16913624451553	-1.44724904894116	-0.19238761751476
N	-3.28073414594873	1.33202429937100	0.29886342248049
N	0.68312592086215	-1.31849979717776	-0.05272824104899
N	3.18024388350173	-1.35919213243639	-0.07230007059478
N	3.26780425263526	1.39796978221385	-0.25883993948828
C	-1.07443808727081	-2.59206071041038	-0.28859204452267
C	-2.54862299092747	-2.76767689473056	-0.36695083339708
C	-4.48864681172029	-1.35896791014635	-0.22694611291048
C	-4.58926565263191	1.12028905955612	0.11652495145368

C	-2.85766925964245	2.68767330043184	0.49700443713101
C	1.12343980211404	-2.57390189046867	-0.21849232007115
C	0.03567425880672	-3.43571687572683	-0.37455005601024
C	-5.16136202116296	-0.13968707817636	-0.08397700435621
C	-5.32706707568800	-2.59606382140845	-0.44215759371651
C	-5.56424787536562	2.28234969339756	0.05365169516207
C	-2.35456692241910	3.41614546494694	-0.59843229159421
C	-2.94474041057120	3.26568563111751	1.77951147974833
C	2.60041010190739	-2.70992913214400	-0.19612534666501
C	-2.05160606759014	4.76499234679833	-0.40826277883517
C	-2.14160658619409	2.75815212949908	-1.95022542866564
C	-2.64092268907972	4.62079414338282	1.91610965988706
C	-3.33788817834631	2.45252036660842	3.00455636447114
C	4.48688504945517	-1.25085260035404	0.13775009484137
C	4.57977753827891	1.21268982741446	-0.14179624088195
C	2.84005635764639	2.64722764324191	-0.81584887982047
C	-2.22291640126146	5.37361187485710	0.82785876687005
C	-0.89894723292979	3.28844307898674	-2.66603896045081
C	-3.37801535671362	2.88045554326715	-2.85204893414490
C	-2.40670432636197	2.73151467256163	4.19680148176968
C	-4.79331458672187	2.69036396978674	3.43602216896009
C	5.15570784178957	-0.02641456498140	0.15655702500893
C	5.31556743553369	-2.49740081858529	0.33803545779302
C	5.53541766458813	2.34926372384684	-0.42417055026507
C	2.91544730382737	2.82420635348591	-2.21073006972969
C	2.38961039645895	3.67141276914265	0.03527158134950
C	2.58599532403081	4.07374188610801	-2.73574418069666
C	3.28759978810590	1.67229992838445	-3.12839280096818
C	2.07024123378582	4.90357183934100	-0.53724957505048
C	2.27669727605086	3.45557141146734	1.53331304031454
C	2.18016101554860	5.11028146882865	-1.90608301287194
C	2.01356894634596	0.96349966641249	-3.61590835063793
C	4.15462273291353	2.09749254013897	-4.31779059272223
C	1.08133573388090	4.19521732881601	2.13910660279366
C	3.56952664339860	3.84612244085251	2.26695535736004

H	-2.81971682730443	-3.21177996737558	-1.33843422165212
H	-2.88668316300213	-3.47514092827141	0.40751565832485
H	0.04815123686196	-4.50424511224614	-0.53109792325937
H	-6.24055668590105	-0.16538057571935	-0.17663378318042
H	-5.09010421825485	-3.07501358768356	-1.39947119742297
H	-6.38906251205486	-2.34736795942062	-0.43918339043009
H	-5.14752079830457	-3.33931657682838	0.34343260921802
H	-6.51221861564523	1.99731483970522	0.51782834622860
H	-5.77970841760504	2.52217462836216	-0.99349273041415
H	-5.19322683810910	3.19120768677336	0.52571842296092
H	2.90423791194751	-3.35330920913350	0.64342653665352
H	2.95698582377406	-3.19601729384221	-1.11838930653720
H	-1.66834942621295	5.34830965274109	-1.23978544931810
H	-1.97448436738574	1.68788540629534	-1.76420944263438
H	-2.71931417594231	5.09246610924017	2.89200698360248
H	-3.25658066036996	1.38955898643172	2.73311232698812
H	-1.99273406592144	6.42805459443043	0.95229774033697
H	-1.02520182247186	4.32288000736528	-3.00640377414496
H	-0.70218701717649	2.67882117211620	-3.55397696586633
H	-0.02003729264471	3.24241587666251	-2.01846074110482
H	-3.66279916012009	3.93150830015447	-2.98177414477311
H	-4.23190508463965	2.33913679204589	-2.43790961058372
H	-3.16505246781566	2.45998806333875	-3.84141407748958
H	-1.35032683646335	2.71745121658265	3.91101071138289
H	-2.56942351558138	1.98762329586671	4.98462070639660
H	-2.61069479363286	3.71448281838323	4.63442960519204
H	-4.96544626500032	3.75711536270804	3.62155330363304
H	-5.00790742077460	2.14601612865997	4.36263842886846
H	-5.50411196248519	2.35675618791351	2.67784779073622
H	6.22672998930560	-0.04835234647758	0.31216278346196
H	5.00333762671177	-3.04056558185076	1.23772910891577
H	6.36867854841350	-2.23636134882682	0.44766334854661
H	5.21990060894080	-3.18640313726471	-0.50859036209159
H	5.73889053439513	2.39543991605834	-1.50132984408884
H	6.48472181151791	2.17713178842760	0.08857621702558

H	5.13260076947584	3.32119188373067	-0.13535558478439
H	2.64040156798422	4.23766917108678	-3.80795368439332
H	3.86092650694668	0.94316619488990	-2.54483368781742
H	1.72479300534655	5.71436594303051	0.09693502380768
H	2.11546964540667	2.38360482279805	1.69009602949094
H	1.92933093687848	6.07860360589850	-2.33064632107968
H	1.38465687181155	0.65264425650814	-2.77360064038110
H	2.26740713305150	0.07522267959358	-4.20599320803143
H	1.42281449139011	1.63829362900600	-4.24564153842962
H	3.59556562925292	2.71291841364463	-5.03083971381744
H	4.50437026836088	1.21227145706853	-4.85981882106971
H	5.03088553881784	2.66940211507083	-3.99419531442557
H	1.24725002935264	5.27856021001460	2.17835402759043
H	0.92017707058332	3.85425744309988	3.16736530241084
H	0.16606046233368	4.00841121903730	1.57183505338659
H	4.41026871844654	3.20939844464122	1.97782633222390
H	3.43227744839052	3.74379397368758	3.34952536011633
H	3.83774547170168	4.88854930706070	2.05592627050462
O	-0.32637306188888	1.31682026639858	1.95996205061526
H	-1.14606084410426	1.06308890562927	2.41258856027543
C	-0.50039624034395	1.04977336271898	0.64521513126081
O	0.47247371625190	1.32021358110575	-0.08851317920522

^{3}Int(C)

104

Ni	-2.02250137802546	0.03892367484317	0.14769362920201
Ni	1.98868124546843	0.11634901222909	-0.07933478249151
N	-0.69185478071097	-1.27386192925657	0.28618579797926
N	-3.18174837620289	-1.48910620391125	-0.02462903114277
N	-3.43374479736013	1.29461916955420	0.02443347523950
N	0.65705861179132	-1.22373063710616	0.38040447660684
N	3.23622673915199	-1.38421932138833	0.25742639484884
N	3.35011275854510	1.35560869287444	-0.58303808625811
C	-1.07942594008979	-2.55356557367595	0.35203190658211
C	-2.53802041967273	-2.78770274492092	0.20857725338900

C	-4.46701009498818	-1.47324077772018	-0.33863194822636
C	-4.70121095612262	1.01086804630080	-0.28450722955265
C	-3.12914074156373	2.63592268874387	0.40974167327899
C	1.11465415977572	-2.48570366300790	0.50655650478426
C	0.04685260561237	-3.37710082383991	0.49755486477467
C	-5.18165865886015	-0.28140248213163	-0.51213499066699
C	-5.22275480747122	-2.76825120242778	-0.52028744744549
C	-5.72881434397105	2.12274112238889	-0.34764331601565
C	-2.46188319120935	3.48212192184207	-0.49875098156323
C	-3.42899609550454	3.04990033772883	1.72292834673407
C	2.59726225218358	-2.65669788131319	0.60940492427428
C	-2.07389121918866	4.74474290561492	-0.05243669774046
C	-2.17187090551636	3.02831826722154	-1.92018156311327
C	-3.01262507033278	4.32224554196236	2.12263002889705
C	-4.13815107019564	2.12568402887613	2.70552608017956
C	4.54813091659458	-1.31071471173029	0.20966399470666
C	4.67019769141490	1.09210569464588	-0.56624607053978
C	2.95916095149481	2.62735100377264	-1.11397524690140
C	-2.33739959555177	5.16088341516963	1.24662006321787
C	-0.90918754387860	3.66097812652831	-2.50418360643266
C	-3.37213534423932	3.28582341513681	-2.84566682616323
C	-3.13722118394880	1.27332355130558	3.50393771144941
C	-5.07422210754857	2.87688782796987	3.65796940398210
C	5.21999367772236	-0.12500155320263	-0.16347260814789
C	5.39791123047001	-2.51367720807652	0.54268821966068
C	5.64618220770673	2.14844970905282	-1.03455507104096
C	2.92155614759297	2.79887468469341	-2.51085720802670
C	2.61444071346376	3.67165177759363	-0.23538570859603
C	2.60746179740371	4.06085764299673	-3.01434465100253
C	3.14780907114668	1.62448595926816	-3.44733820532517
C	2.31075532773690	4.91708622054597	-0.78807375429569
C	2.58246527208294	3.45315688632420	1.26699438844261
C	2.32525647189954	5.11903161100360	-2.16182584608268
C	1.80528125541842	0.93815610089991	-3.74899917835647
C	3.86195408394076	2.00539471750223	-4.74781286532587

C	1.62194441842381	4.40190966007085	1.98813620109967
C	3.98212658094913	3.54937838480415	1.89490886345909
H	-2.71668856350569	-3.48868022976736	-0.62175233538116
H	-2.93745126661916	-3.27062993977157	1.11651449993037
H	0.07555423655254	-4.45521486559779	0.56384682667014
H	-6.22562319661872	-0.36888996343347	-0.78762869805534
H	-4.81042456330375	-3.35286669957827	-1.35138832609739
H	-6.27486317271252	-2.57311848490707	-0.73200734535582
H	-5.16425905799371	-3.39540434293324	0.37669447652233
H	-6.20447885934707	2.25912466337792	0.63160112961536
H	-6.51497644218002	1.86076030020787	-1.06015587684128
H	-5.29346707165224	3.08259937055618	-0.62920528867070
H	2.86714954560823	-2.96423095559501	1.63392256831590
H	2.92446919016207	-3.47110787582053	-0.05620851122736
H	-1.53794045537125	5.40771382532119	-0.72429294675352
H	-2.01157618678542	1.94299289025348	-1.88497889091670
H	-3.21812345245761	4.66406707472837	3.13276150595299
H	-4.74831629564662	1.42103707418907	2.13177251344873
H	-2.01312833772228	6.14335356718852	1.57805480965131
H	-1.03751982552423	4.73175691689065	-2.70329481398121
H	-0.67040750846011	3.18343637579911	-3.46001927378433
H	-0.05078442445133	3.53030529480974	-1.84082043291990
H	-3.64767262506242	4.34733855082022	-2.83634252449662
H	-4.24708923144253	2.69968866092564	-2.55343054366156
H	-3.11569474032681	3.01032205156435	-3.87482262118956
H	-2.58626075713924	0.58941037019694	2.84360211524278
H	-3.66175451936545	0.65220121322469	4.23884818538122
H	-2.42681662318838	1.90725660406843	4.05067521577851
H	-4.51960462717769	3.47338288666274	4.39058984568348
H	-5.68615935647345	2.16294992853899	4.21933043856479
H	-5.74347158545394	3.55103988536216	3.11302123270047
H	6.30200290104217	-0.17997891232007	-0.17435520826011
H	5.16759393642386	-2.89197986528207	1.54517361998857
H	6.45979395571558	-2.26745004913153	0.50355344120670
H	5.21096930254906	-3.33303285301150	-0.16154649940403

H	5.63112217062704	2.22010982840867	-2.12852601062229
H	6.66196123229983	1.89782640935558	-0.72289219037656
H	5.38334548805259	3.13909469817800	-0.65506449253676
H	2.57830899619142	4.21872005973480	-4.08830089549731
H	3.77622575866546	0.88970683342345	-2.93120613243883
H	2.04826413767368	5.74236584681999	-0.13394097145394
H	2.21346426465518	2.43193338080412	1.43066762709176
H	2.08892131554817	6.09784296598663	-2.56989702689752
H	1.28569454052046	0.65593995876030	-2.82723060225863
H	1.95857343351314	0.03898965757829	-4.35686176670736
H	1.14801917423718	1.61906913059156	-4.30161068346228
H	3.22606910616475	2.61520654893483	-5.39854519089017
H	4.12320392912352	1.10125277841294	-5.30799453198999
H	4.78234756326582	2.56719912653752	-4.55450583790237
H	2.01577495031307	5.42482428744360	2.02605221028658
H	1.48553834413205	4.06652820485922	3.02158654200230
H	0.63909065239655	4.41664178934383	1.50883150327023
H	4.65196742864493	2.76645469141263	1.52908259755249
H	3.91278409486195	3.44419186163192	2.98368975789878
H	4.43643712719898	4.52368248978609	1.67744776140216
O	-0.42963329823675	1.93803977547943	1.54408419719647
H	-1.25142965520897	1.88829982115069	2.05547458700381
C	-0.60971571962442	1.27864876471809	0.37099140042567
O	0.33480628599053	1.37481979936106	-0.42589653794156

$^{1}CO_2$

3

O	0.00000000185588	0.00000000185490	-1.16342529600561
C	-0.00000000371176	-0.00000000370981	0.00000000000495
O	0.00000000185588	0.00000000185490	1.16342529600066

$^{1}Ni(CO)_4$

9

Ni	-0.00072651415233	-0.00039401733605	-0.00000000129051
C	1.81352614388479	0.00018675306816	0.00000000098717
C	-0.60494105921858	1.71092135917950	-0.00000000051861

C -0.60517673175843 -0.85564100650516 -1.48171159314665
C -0.60517673063673 -0.85564100607869 1.48171159120819
O 2.95585055704630 0.00073122129426 0.00000000297109
O -0.98384875243183 2.78853237120098 0.00000000029015
O -0.98473995855160 -1.39434783775347 -2.41478035566405
O -0.98473995418158 -1.39434783706953 2.41478035516321

[1]2^CO^

102

Ni 1.50137297998378 -0.11318603322297 0.13285010144391
N -1.07160020069444 -1.42747172705853 -0.25399610554134
N -3.44589558557162 -3.28326094447703 1.13083553877589
N -2.69845105674733 -3.51973802467497 3.67635257642787
N 0.24202464335278 -1.46936938367754 0.03362105737338
N 2.65016970312158 -1.48394977713780 0.73673207353949
N 2.78931503348074 1.25760614748820 0.33654277228822
C -1.50075707172771 -2.69092866006224 -0.12101096260515
C -2.95880136588196 -3.00822494525127 -0.22031468563039
C -4.68067510653012 -3.06038969148555 1.60899184635781
C -3.94202502404673 -3.27215318536372 3.98594844815883
C -1.67975707531952 -3.69161286577566 4.62093311072222
C 0.64488235284785 -2.71864104839243 0.35514719290632
C -0.45515560103909 -3.55423842192606 0.26451828925944
C -4.93528739270028 -3.10061760646622 2.96803509317873
C -5.77315737557313 -2.74794034005842 0.62933744156139
C -4.41984313111558 -3.09725587477020 5.41304250438688
C -0.66890539813882 -2.70705154129023 4.70795359032835
C -1.58642190806769 -4.88518002788166 5.37418118310461
C 2.06888347050189 -2.84405271888583 0.73104146275838
C 0.37556756913581 -2.89982014038251 5.61112937160875
C -0.74769519869615 -1.47277930773338 3.82823098254797
C -0.51822290219182 -5.03480083730765 6.25811290972505
C -2.57659420692070 -6.01211304031527 5.13519885846892
C 3.88634190094513 -1.33747054548897 1.19281499715434
C 4.02036703199014 1.11560488873650 0.82561000457936

C	2.39169551458511	2.56271584210223	-0.11485786970368
C	0.44889723300939	-4.04639808787616	6.39290522078380
C	0.60124373366243	-0.78058258702408	3.62565963110756
C	-1.77508045561012	-0.46410326887736	4.36460327146882
C	-2.20802076221484	-6.75081174793744	3.83736027461006
C	-2.69368376062708	-6.99815860527668	6.29857935881829
C	4.54364620902494	-0.10482492238528	1.24607903298532
C	4.64281856378534	-2.54245014858542	1.69549287122776
C	4.91230544369925	2.32899780955210	0.92001615236175
C	2.66735561236914	2.93873939216923	-1.44354238526792
C	1.67981854937868	3.40710364350516	0.75734430884245
C	2.17790629195111	4.16385862544667	-1.89481769807546
C	3.43757281556795	2.03210963347043	-2.38820232206238
C	1.21655693115195	4.62533790121763	0.25926714138221
C	1.39463827269684	3.02619612488855	2.19926615161711
C	1.45281903461259	4.99968778457340	-1.05567207190315
C	2.48542460842084	1.31382052221676	-3.35614271718037
C	4.52167504825296	2.78927212032208	-3.16654950960292
C	-0.11006439151905	2.84368279658831	2.44549502366915
C	1.96829324859444	4.06012377385374	3.17913593241435
H	-3.47371844686333	-2.14752310654659	-0.66203677538938
H	-3.12480771309754	-3.87730704602543	-0.87649517801153
H	-0.50694627994628	-4.61431694813239	0.47000300149803
H	-5.95575555691540	-2.92816838658964	3.29138611584870
H	-5.61231878280285	-1.76641537666197	0.16719927658536
H	-6.74237900343777	-2.73176759586877	1.12995072034525
H	-5.80510414124897	-3.48732092840028	-0.17895462954829
H	-5.19015512781692	-3.84032550054609	5.64993962889022
H	-4.88058772365729	-2.11020527469765	5.53178016774406
H	-3.60215109060044	-3.19605865739412	6.12993340200145
H	2.16108522976184	-3.31382011607825	1.72145016894012
H	2.61274598850525	-3.48395195903722	0.01800857778972
H	1.15017949928368	-2.14442008000732	5.70498765516067
H	-1.10028624594894	-1.80849829899431	2.84245520185955
H	-0.43708030473733	-5.93832210067873	6.85465226081559

H	-3.56840406602755	-5.57153978620845	4.98300357978894
H	1.26668804835918	-4.17618610168862	7.09611153802295
H	0.93626934663713	-0.26342587696389	4.53348933333420
H	0.51763052854460	-0.03095703804305	2.83087663556345
H	1.38215236173071	-1.48904937760920	3.33065827393726
H	-1.47183486105808	-0.10119532001770	5.35433157851083
H	-2.76660773279819	-0.91476017919819	4.45356181306466
H	-1.85541175968285	0.39639447621834	3.69008950489833
H	-2.18790128576873	-6.05765523945912	2.99088152914658
H	-2.93533245687267	-7.54176653615612	3.62028552418135
H	-1.21697129795728	-7.20958931563468	3.93355508332416
H	-1.78022619302466	-7.59042259424241	6.42380247020797
H	-3.51054349015785	-7.70191351369202	6.10670678970892
H	-2.89965631403234	-6.48447062761020	7.24390102719009
H	5.55140111459718	-0.10062227218729	1.63970960655460
H	4.15813102609059	-2.96639503193726	2.58290541905383
H	5.66296138451422	-2.26658537338824	1.96386175209658
H	4.68507437493604	-3.33424176694816	0.94018608437552
H	5.15143385340248	2.71182005436830	-0.07827514314205
H	5.84379091279541	2.07870276641900	1.42980305833180
H	4.41823836342553	3.14395892554809	1.45682105590466
H	2.36329405966179	4.46868636939356	-2.92088956695519
H	3.93474215058772	1.26166150776712	-1.78845571175067
H	0.65591336832474	5.28958608597716	0.91150073254104
H	1.88397951172657	2.06645102480544	2.40091282070904
H	1.07467571749562	5.94753652429256	-1.42750438593937
H	1.77843453436216	0.67265616184889	-2.81959177781827
H	3.04998204520596	0.68144272104718	-4.05031492062547
H	1.90796699314639	2.03771719361158	-3.94229132779276
H	4.08890868518262	3.48138122258143	-3.89668050759701
H	5.14791734559490	2.08167642662977	-3.72003683734626
H	5.16712023511539	3.36885728499409	-2.49828848579456
H	-0.65529284548162	3.77735020681803	2.26724480431596
H	-0.28957841150368	2.53913671074482	3.48230420156418
H	-0.53889198931047	2.07682157926102	1.79284665712501

H	3.03857305285579	4.22192072668472	3.01637029679425
H	1.82779269100507	3.72004469911862	4.21075748448113
H	1.46569818632820	5.02804729420116	3.07640505595363
H	-2.74737296404922	-3.43317288138703	1.87416801773001
C	0.27655481096797	0.97436590398765	-0.59033126209864
O	-0.52577014653545	1.60361568563023	-1.09574112045347

$^{3}2^{CO}$

102

Ni	1.38187062492405	-0.18508916058291	-0.34693729684631
N	-1.53818664161882	-0.97728423411124	-0.15107435649707
N	-3.84813941550496	-2.77997077557697	1.65916087311456
N	-2.60794324578914	-3.41931719885448	3.87814336046190
N	-0.20792792977227	-1.19563060212602	-0.02654094290143
N	2.24849037923768	-1.63680430645783	0.67336213339618
N	2.66875106692812	1.13024534521672	0.24018558643662
C	-2.12732672038465	-2.12866733977478	0.17778223229019
C	-3.61407098530387	-2.24276817878702	0.32280690794644
C	-4.98486715700187	-2.78608602831341	2.37137407744702
C	-3.76968390618636	-3.37692822311822	4.47053088205846
C	-1.37369394665069	-3.63900582554427	4.49532345405512
C	0.03214567899533	-2.47397203596451	0.39519323707619
C	-1.17255736829322	-3.12251531099554	0.52216469970840
C	-4.96150450238788	-3.11463562071838	3.71737303534235
C	-6.25956390569821	-2.41736614288747	1.67440626548356
C	-3.93711924019071	-3.55424716940917	5.96436631508037
C	-0.45854817286220	-2.56126265344255	4.55428341121220
C	-0.97993097225951	-4.93699464936020	4.89588313323210
C	1.45062965563403	-2.87275290781968	0.64948891505999
C	0.81821532027649	-2.79252967892206	5.06246580149542
C	-0.89352091515896	-1.18557792328525	4.08815003370374
C	0.31239065275500	-5.11927280150179	5.38967005984106
C	-1.91989910563034	-6.11304791758812	4.69490647478541
C	3.26349598082172	-1.52547500588686	1.50214319232219
C	3.64287596884743	0.94167525080676	1.13906039264703

C	2.48931585791045	2.42762642818765	-0.33096212849583
C	1.20614648871093	-4.05952590444545	5.48397428899423
C	0.26273327226378	-0.28590623278612	3.65028910772274
C	-1.73028340848731	-0.48224461619620	5.16809893533727
C	-1.91228410610350	-6.54040034524207	3.21783926300258
C	-1.62022504499065	-7.30995269523068	5.59909183784172
C	3.93669611860473	-0.29762346080663	1.70533756306042
C	3.73401968547137	-2.70660453354239	2.31762263920215
C	4.48024672821120	2.12318952163772	1.56245208831705
C	3.21130660646715	2.76669853168434	-1.49419589090232
C	1.51776486212904	3.29438353909118	0.20495171730860
C	2.91543385386546	3.97405278992572	-2.12609359569422
C	4.27427606495986	1.84884131243974	-2.07503310925778
C	1.25783627684062	4.48989016967130	-0.46534286934893
C	0.74670966265845	2.94815155338595	1.46540203003630
C	1.94189065680139	4.82778909734308	-1.62474300473868
C	3.77394877201779	1.16315726261582	-3.35465161075847
C	5.58962955856115	2.59136741379375	-2.34890385611895
C	-0.71498791775575	2.60246167947507	1.14367667567343
C	0.81778078108226	4.07514593844402	2.50506370627980
H	-4.05817956848568	-1.24723955128414	0.18826082263202
H	-4.04084922344256	-2.90766843832738	-0.44594615600323
H	-1.35364246804470	-4.13819121822963	0.84510562450419
H	-5.90450556617921	-3.11651583690521	4.25218031416068
H	-6.22926463430908	-1.37481692475130	1.33538628222676
H	-7.11455236326415	-2.53661403389342	2.34146291293766
H	-6.41463125342010	-3.04185135904736	0.78686847051833
H	-4.55459967326263	-4.43518199585449	6.17528110235273
H	-4.45812994576160	-2.68982187270216	6.39028955073122
H	-2.97255341464070	-3.67161600442790	6.46382898481281
H	1.50272608393737	-3.42534185710471	1.59638582741910
H	1.82050010060627	-3.54539433547125	-0.14206055104320
H	1.52338729303683	-1.96875800310983	5.12937591764086
H	-1.54089796889272	-1.33771050466380	3.21374903008180
H	0.62939115456631	-6.10681571008420	5.71027813450304

H	-2.93745425635214	-5.77752180917163	4.92640074411210
H	2.20340328384057	-4.22193691858273	5.88374864562144
H	0.88538160388113	0.02950890421254	4.49621491489346
H	-0.13986131877002	0.61874219075012	3.18463787978711
H	0.90244724985775	-0.78009746384236	2.91203279069399
H	-1.12715304121783	-0.31886368254839	6.06930910756777
H	-2.60401075194420	-1.07746973363263	5.44771769477178
H	-2.08339770014438	0.49158301807632	4.80898685811998
H	-2.22487763924278	-5.71224167384480	2.57501268838993
H	-2.59697545872491	-7.38032866196187	3.05265010015823
H	-0.90464251414760	-6.85316285848302	2.91981619457292
H	-0.67904819337973	-7.80007495918429	5.32584982092598
H	-2.41371523703605	-8.05811592405944	5.49954347624386
H	-1.55836281471449	-7.01490590261807	6.65206351719769
H	4.75829105313710	-0.31762546919013	2.41105837701307
H	2.99986531903945	-2.95356159413874	3.09560014853811
H	4.68326700280697	-2.48355183967189	2.80726695097999
H	3.86324180282629	-3.59652762749166	1.69354442271330
H	5.01342614971820	2.55097629912666	0.70677997596747
H	5.20815695639472	1.83552433940075	2.32252438982935
H	3.84319202669039	2.91965719039881	1.96238287363788
H	3.45226164784134	4.24967669040756	-3.02977426940971
H	4.48185509038640	1.06285104034447	-1.34101060290632
H	0.50269587165156	5.16656354690916	-0.07471276911696
H	1.20958034901591	2.05958003033328	1.91033820679188
H	1.71916565192198	5.75960057417763	-2.13629020350250
H	2.88752257380092	0.55191595759628	-3.15745020897361
H	4.55022069693132	0.51103445319324	-3.77034391395858
H	3.50796132881463	1.90621468984333	-4.11504036656435
H	5.48472129475559	3.31117356722107	-3.16777768120921
H	6.37065844177987	1.87933775338589	-2.63647996379889
H	5.93474204107163	3.14020634076066	-1.46649406022700
H	-1.19888629773985	3.42635235802000	0.60672990323759
H	-1.27961321560981	2.42522137762957	2.06617155063915
H	-0.79865317749311	1.69951519950983	0.53042438101373

H	1.85239661488580	4.37273434667419	2.70603609251892
H	0.36670330677858	3.74354738308458	3.44670292082868
H	0.27174546433795	4.96498555798472	2.17368910342576
H	-3.02077804677242	-2.99259383758092	2.24375023665428
C	0.59778509345521	0.82214499777020	-1.76362793587614
O	-0.10604675772034	1.37099573360917	-2.46980419819165

^{1}2a^{CO}

102

Ni	2.00592636710216	0.16175707099822	-0.00129094551271
N	-0.62566244440005	-1.05756450856416	-0.22471790971822
N	-3.36370721639211	-1.39097148858524	-0.20339906277746
N	-3.62914046523687	1.35035239995005	-0.13347353692579
N	0.68002139210890	-1.12362607218271	0.09116088398531
N	3.13475377905935	-1.23308206684620	0.56964126869672
N	3.39564921231822	1.43477870204996	-0.16593594088183
C	-1.11304324787908	-2.27934382449326	0.01676201535421
C	-2.55119503400962	-2.59266357701999	-0.26467438421390
C	-4.70862854813021	-1.37438168818527	-0.17758169620907
C	-4.90647130763284	1.12026170770776	-0.01687784381308
C	-3.11856245603569	2.64795800707984	0.03596802509112
C	1.02786132235671	-2.35645586588102	0.51547929584332
C	-0.10990588479615	-3.14966534269134	0.48856198057929
C	-5.43990870131415	-0.20644799205258	-0.10659538433307
C	-5.41860460099681	-2.69835333876321	-0.20111424338287
C	-5.91213457313311	2.21968581370246	0.26366360755305
C	-2.76384283373051	3.40624824948617	-1.09862796212705
C	-2.86259059035651	3.13847634287719	1.33613150903141
C	2.45610776180717	-2.51218055493519	0.87390829764929
C	-2.24334622574805	4.68646843127985	-0.91375480154607
C	-2.92366640442840	2.80602334161766	-2.48274917681962
C	-2.34169612247608	4.42449609743283	1.47291266511823
C	-3.06064689663000	2.22890409581411	2.53545746878521
C	4.45058498527413	-1.16498023287085	0.73190594740225
C	4.69186782234461	1.20774814208605	0.02397922004393

C	2.98275036748594	2.73727063303506	-0.61007687927162
C	-2.04916221318172	5.20502810744269	0.35971617414077
C	-1.89165939752219	3.32864964121154	-3.48759411173239
C	-4.34422638497559	3.01304798505272	-3.03017848863954
C	-1.86644987658904	1.26349550665421	2.64324465270826
C	-3.26721868179483	2.97366702744767	3.85519164672923
C	5.19843327850648	-0.01493088091538	0.46848105081858
C	5.20445818320015	-2.38179639396911	1.21070952336588
C	5.69202395766779	2.29404351962532	-0.29582998685527
C	2.99038808048004	3.03345194450122	-1.98441307723247
C	2.47978217743072	3.64684264237006	0.34268407275568
C	2.48729514736756	4.26997742506795	-2.39189413526531
C	3.45288276535964	2.02662548494916	-3.02276154753052
C	1.99920119289110	4.87239767736824	-0.11549923682296
C	2.41847833418095	3.28590855801957	1.81784290578921
C	2.00010220843724	5.18341852961481	-1.46913231194946
C	2.24553536196657	1.37859888796055	-3.71848918171371
C	4.39762017012845	2.64935459349504	-4.05903475688166
C	1.26298220090763	3.97340680162768	2.55045205058443
C	3.74787663272766	3.57960183145804	2.53138393493348
H	-2.63310334872215	-3.06901180161422	-1.25808897849308
H	-2.89644515267665	-3.33649582181216	0.46682080576377
H	-0.20538413367163	-4.18979518010714	0.76690162709792
H	-6.51835882694594	-0.30724690037289	-0.06720061946382
H	-5.11410215478093	-3.30027258835082	-1.06536927931705
H	-6.49792561108282	-2.54903388160664	-0.24675874238704
H	-5.19313886182621	-3.28409875633137	0.69826671785248
H	-6.09760446731153	2.28423644382291	1.34350212015647
H	-6.86947629894862	2.00247890920771	-0.21917106696177
H	-5.54832980564592	3.19526584577884	-0.06651091895092
H	2.56758580337763	-2.75812904746219	1.94171100949975
H	2.91104445959960	-3.33639577704032	0.30460885398685
H	-1.97190076725654	5.28462305986878	-1.77870219514707
H	-2.76811871102891	1.72439917161485	-2.36789265806769
H	-2.15961060185168	4.82778131898071	2.46493651655416

H	-3.95387306069622	1.61811789162054	2.35556306182495
H	-1.65335785111523	6.20903783313298	0.48675164144656
H	-2.09333878707026	4.36741105758066	-3.77464490062590
H	-1.92747742779368	2.72853899396397	-4.40357837300240
H	-0.87572137077172	3.27920365659625	-3.08159166399274
H	-4.57613922850770	4.08302234911535	-3.09484847755927
H	-5.09291500153376	2.53973584085981	-2.38915305825400
H	-4.43810615477678	2.58158564556162	-4.03381005371426
H	-1.73523540425871	0.68947583618761	1.72099900436846
H	-2.00908269522496	0.55797637789394	3.47045461539384
H	-0.94420561051733	1.82756079399176	2.82963958274024
H	-2.35617279308172	3.49352309502273	4.17393291990330
H	-3.52573038893584	2.26242294955527	4.64696154403028
H	-4.07242214807642	3.71289122600086	3.78175210963489
H	6.26947460605887	-0.07976139605396	0.61006103305306
H	4.81359227395804	-2.74246752537294	2.16834285690032
H	6.26211877124921	-2.14909325284992	1.33710419842740
H	5.11794884524653	-3.20658135909462	0.49425106963904
H	5.88823575191506	2.30592983088400	-1.37480632553929
H	6.63658419634501	2.10229622394517	0.21707050906403
H	5.32757064230259	3.28784130465037	-0.02773362235099
H	2.46338941664230	4.51493424097384	-3.45007669991550
H	3.99901342870206	1.22827555151361	-2.50783312676772
H	1.58653593276490	5.58443520002076	0.59109438647354
H	2.24941876759748	2.20130213045301	1.87924403684146
H	1.60150448413424	6.13610776031459	-1.80547335595734
H	1.61137773048644	0.84269381319457	-3.00457934306921
H	2.57878284381104	0.66065389852523	-4.47621764348978
H	1.62822096401249	2.13769582850893	-4.21229976354333
H	3.87174372714674	3.35401843420290	-4.71198110165814
H	4.82234490760562	1.86796451501839	-4.69818608081705
H	5.22162397550410	3.19000911439838	-3.58145158051033
H	1.45388159164666	5.04300209692605	2.69635765256556
H	1.14019570689917	3.52957068332432	3.54411390494807
H	0.31925783824104	3.86759542401572	2.00615042458604

H	4.56764796248232	2.97130494375226	2.13970798210101
H	3.65611627814160	3.36482916153229	3.60186440294746
H	4.01743258268664	4.63629053869233	2.41775007727123
H	-2.90041954592414	-0.47841030371491	-0.22889623048485
C	0.73229673103156	1.32755066402271	-0.47846221147177
O	-0.11770134827449	2.01944044752878	-0.77696568836296

5. References

(1) *ORCA version 5.0.4, an ab initio, DFT and semiempirical SCF-MO package*; F. Neese, Technical Directorship F. Wennmohs, with contributions from D. Aravena, M. Atanasov, A. A. Auer, U. Becker, G. Bistoni, D. Bykov, V. G. Chilkuri, D. Datta, A. K. Dutta, S. Ehlert, D. Ganyushin, M. Garcia, Y. Guo, A. Hansen, B. Helmich-Paris, L. Huntington, R. Izsák, M. Kettner, C. Kollmar, S. Kossmann, M. Krupiˇcka, L. Lang, M. Lechner, D. Lenk, D. G. Liakos, D. Manganas, D. A. Pantazis, A. Papadopoulos, T. Petrenko, P. Pinski, P. Pracht, C. Reimann, M. Retegan, C. Riplinger, T. Risthaus, M. Roemelt, M. Saitow, B. Sandhöfer, I. Schapiro, A. Sen, K. Sivalingam, B. de Souza, G. Stoychev, W. Van den Heuvel, B. Wezisla, and with contributions from collaborators M. Kállay, S. Grimme, E. Valeev, G. Chan, J. Pittner, M. Brehm, L. Goerigk, V. Åsgerisson, L. Ungur, 2023. https://orcaforum.kofo.mpg.de/.

(2) Neese, F. Software update: The ORCA program system—Version 5.0. *WIREs Comput Mol Sci* **2022**, *12* (5). DOI: 10.1002/wcms.1606.

(3) Grimme, S.; Hansen, A.; Ehlert, S.; Mewes, J.-M. r^2SCAN-3c: A "Swiss army knife" composite electronic-structure method. *J. Chem. Phys.* **2021**, *154* (6), 64103. DOI: 10.1063/5.0040021.

(4) Caldeweyher, E.; Ehlert, S.; Hansen, A.; Neugebauer, H.; Spicher, S.; Bannwarth, C.; Grimme, S. A generally applicable atomic-charge dependent London dispersion correction. *J. Chem. Phys.* **2019**, *150* (15), 154122. DOI: 10.1063/1.5090222.

(5) Kruse, H.; Grimme, S. A geometrical correction for the inter- and intra-molecular basis set superposition error in Hartree-Fock and density functional theory calculations for large systems. *J. Chem. Phys.* **2012**, *136* (15), 154101. DOI: 10.1063/1.3700154.

(6) Fukui, K. The path of chemical reactions - the IRC approach. *Acc. Chem. Res.* **1981**, *14* (12), 363–368. DOI: 10.1021/ar00072a001.

(7) Riplinger, C.; Pinski, P.; Becker, U.; Valeev, E. F.; Neese, F. Sparse maps--A systematic infrastructure for reduced-scaling electronic structure methods. II. Linear scaling domain based pair natural orbital coupled cluster theory. *J. Chem. Phys.* **2016**, *144* (2), 24109. DOI: 10.1063/1.4939030.

(8) Guo, Y.; Riplinger, C.; Becker, U.; Liakos, D. G.; Minenkov, Y.; Cavallo, L.; Neese, F. Communication: An improved linear scaling perturbative triples correction for the domain based local pair-natural orbital based singles and doubles coupled cluster method DLPNO-CCSD(T). *J. Chem. Phys.* **2018**, *148* (1), 11101. DOI: 10.1063/1.5011798.

(9) Perdew, J. P.; Ernzerhof, M.; Burke, K. Rationale for mixing exact exchange with density functional approximations. *J. Chem. Phys.* **1996**, *105* (22), 9982–9985. DOI: 10.1063/1.472933.

(10) Adamo, C.; Barone, V. Toward reliable density functional methods without adjustable parameters: The PBE0 model. *J. Chem. Phys.* **1999**, *110* (13), 6158–6170. DOI: 10.1063/1.478522.

(11) Drosou, M.; Mitsopoulou, C. A.; Pantazis, D. A. Reconciling Local Coupled Cluster with Multireference Approaches for Transition Metal Spin-State Energetics. *J. Chem. Theory Comput.* **2022**, *18* (6), 3538–3548. DOI: 10.1021/acs.jctc.2c00265.

(12) Neese, F.; Valeev, E. F. Revisiting the Atomic Natural Orbital Approach for Basis Sets: Robust Systematic Basis Sets for Explicitly Correlated and Conventional Correlated ab initio Methods? *J. Chem. Theory Comput.* **2011**, *7* (1), 33–43. DOI: 10.1021/ct100396y.

(13) Altun, A.; Neese, F.; Bistoni, G. Extrapolation to the Limit of a Complete Pair Natural Orbital Space in Local Coupled-Cluster Calculations. *J. Chem. Theory Comput.* **2020**, *16* (10), 6142–6149. DOI: 10.1021/acs.jctc.0c00344.
